# Author Correction: Seismic detection of a 600-km solid inner core in Mars

**DOI:** 10.1038/s41586-025-09981-1

**Published:** 2025-12-03

**Authors:** Huixing Bi, Daoyuan Sun, Ningyu Sun, Zhu Mao, Mingwei Dai, Douglas Hemingway

**Affiliations:** 1 National Key Laboratory of Deep Space Exploration/School of Earth and Space Sciences, University of Science and Technology of China, Hefei, China; 2 CAS Center for Excellence in Comparative Planetology, Hefei, China; 3 State Key Laboratory of Precision Geodesy, University of Science and Technology of China, Hefei, China; 4 Institute for Geophysics, Jackson School of Geosciences, University of Texas at Austin, Austin, TX USA

**Keywords:** Planetary science, Astronomy and planetary science, Seismology

Correction to: *Nature* 10.1038/s41586-025-09361-9 Published online 3 September 2025

In the version of the article initially published, in the “Composition of the Martian core” section of the Methods, the text “The influence of O, C and S on the OC velocity and density are calculated as follows” incorrectly included a citation to ref. 54 which has now been replaced with ref. 70 (Huang, D. et al. Thermoelastic properties of liquid Fe-rich alloys under martian core conditions. *Geophys. Res. Lett.* **50**, e2022GL102271 (2023)) in the HTML and PDF versions of the article.

