# Supplementary material for: Seismic detection of a 600-km solid inner core in Mars
Source: Nature. 2025 Sep 3;645(8079):67–72. doi: 10.1038/s41586-025-09361-9 (PMC12408336; doi:10.1038/s41586-025-09361-9)
Supplement: Supplementary file 1 — This file contains additional information on generating synthetics (section 2), core phases detection (section 3), inversion (section 4), validation of the inverted model (section 5), analysis of the density (section 6) and composition and dynamics of the Martian inner core (section 7). [file 41586_2025_9361_MOESM1_ESM.pdf]

nature portfolio

https://doi.org/10.1038/s41586-025-09361-9

Supplementary information

Seismic detection of a 600-km solid inner core in Mars

In the format provided by the authors and unedited

Supplementary Information A for

Seismic Detection of a 600-km Solid Inner Core in Mars

Huixing Bi, Daoyuan Sun*, Ningyu Sun, Zhu Mao, Mingwei Dai, Douglas Hemingway

*Corresponding author: sdy2014@ustc.edu.cn

Contents

The PDF file includes:

1 Overview....2
2 Generating synthetics....2
2.1 Martian core velocity models....2
2.2 Travel times of core phases....3
2.3 Synthetic waveforms....4
2.4 Vespagram of the synthetics....7
2.5 The effect of uncertainty in source depth....8
3 Data process and core phases detection....8
3.1 Data....8
3.2 Phase detection process....9
3.2.1 Summary of phase detection process....9
3.2.2 Stage I: Vespagram analysis....9
3.2.2.1 Feasibility of vespagram analysis of polarization filtered waveforms..9
3.2.2.1.1 Mantle phases P, S, ScS, PPPP and PPPPP....10
3.2.2.1.2 P′P′r_ab and P′P′n....11
3.2.2.1.3 PKKP....12
3.2.2.2 Assessment of vespagram result robustness....15
3.2.2.3 Uncertainties from vespagram analysis....16
3.2.3 Stage II: Analysis for individual events....18
3.3 Identification of PKiKP phase....20
3.4 Amplitude and polarity of PKKP and PKiKP....26
3.5 Summary of core phase picking....28
4 Inversions....30
4.1 Seismic model parameterization....30
4.2 Inversion results....32
5 Further supports for the core with an IC....41
5.1 PKiKP for large distance events....41
5.2 P′P′r_df phase....44
5.3 PKIIKP phase....45
6 Density of the IC....45
6.1 The amplitude ratio between PKiKP and PKKP....45
6.2 Geodesy constraints....46
7 Composition and dynamics of the Martian core....47
7.1 Mineral composition....47
7.2 Implications for dynamics....48
References....49

1 Overview

This supplement contains additional information on generating synthetics (**Section 2**), data process and core phases detection (**Section 3**), seismic inversion (**Section 4**), validation of the inverted inner core model (**Section 5**), analysis of the Martian core density (**Section 6**), and composition of Martian inner core (**Section 7**).

2 Generating synthetics

2.1 Martian core velocity models

The currently available seismic velocity models of the Martian core are derived from inversions based on the differential travel times of core-reflected seismic phase ScS [1], core-transiting phase SKS [2], and P wave diffracted along a stratified core-mantle boundary (CMB) of Mars [3, 4]. Supplementary Fig. 1 illustrates the P-wave velocity and density profiles of different models.

Stähler et al. (2021) invert the Martian interior structure using three different strategies based on the ScS differential time and derive the AK_subset models with a "geophysical" inversion, using 6 different mantle compositions and fitting the observed degree-2 Love number (k2), mean normalized moment of inertia, and seismic data [1]. The AK_mean model is the average model derived from the inverted models. It is worth noting that the inversion of the AK_mean model does not include seismic phases directly sampling the core.

The SKS_GP and SKS_GD velocity models correspond to results from "geophysical" and "geodynamical" inversions in Irving et al. (2023) [2], respectively. In these inversions, the travel times of core-transiting seismic phase SKS, identified for two farside seismic events S1000a and S0976a, are included, which provides crucial constraints on the upper ~1,000 km structure of the Martian core. In addition, the mean density (3.935 ± 0.0012 g/cm^3), and the mean normalized moment of inertia (MOI) of Mars (0.3634 ± 0.00006) are also fitted [5]. The MSL_IPGP [4] and MSL_ETH [3] further include an anomalously slow P wave diffracted along the CMB of the impact event S1000a in the inversions. Both models feature a ~150-km-thick low velocity molten silicate layer (MSL) overlying the liquid core. Here, we select a mean model from the 1,000 velocity models within MSL_ETH for further analysis. For MSL_IPGP, we adopt the best inversion model provided by them for subsequent analysis.

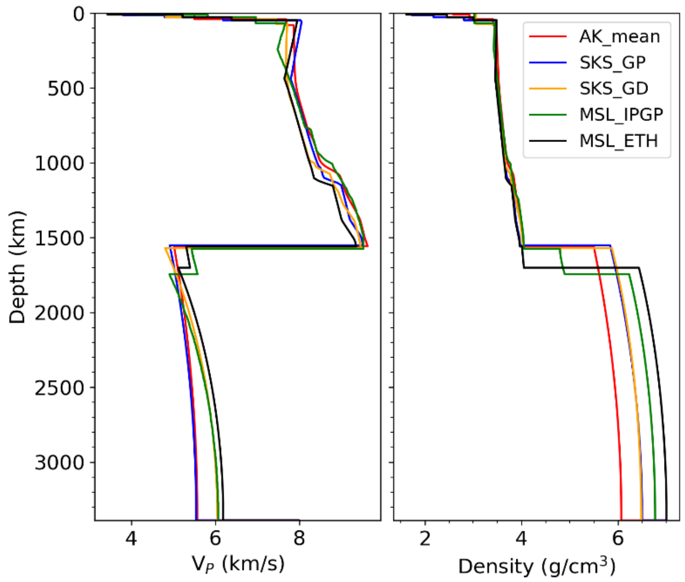

Supplementary Fig. 1. P wave velocity (left) and density (right) profiles of Mars models from previous studies. Note that here we only plot the best (SKS_GP, SKS_GD and MSL_IPGP) or mean (AK_mean and MSL_ETH) models in each group of models for a better illustration.

2.2 Travel times of core phases

We calculate ray paths and travel times for mantle phases PPPP and PPPPP along the major arc, as well as core-transiting phases PKKP and PKPPKP(P′P′) with the TauP Toolkit [6]. An example for the SKS_GD model is shown in Supplementary Fig. 2. In particular, the P′P′ phase has three arrivals (Supplementary Fig. 2b). Among these, one P′P′ arrival (herein referred to as P′P′n (n stands for normal)) represents a ray taking off in the same direction as the direct P phase. The other two arrivals have rays leaving from the event towards the station along the major arc (herein referred to as P′P′r (r stands for reversed)), sampling different depths of the core. The one sampling the shallower core is labeled as P′P′r_ab, while the other sampling the deeper core with a faster velocity is labeled as P′P′r_bc. Note that the travel times and distance ranges for having different branches of the P′P′ vary across different velocity models as shown in the synthetics in the next section.

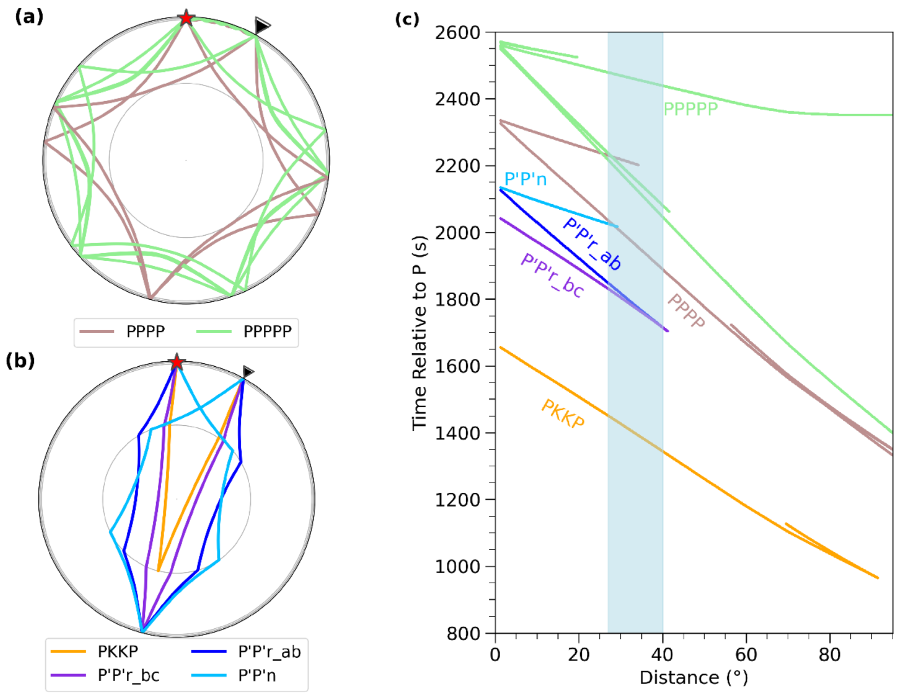

Supplementary Fig. 2. Ray paths and travel time curves for various mantle and core phases for the SKS_GD model in Supplementary Fig. 1. Ray paths of (a) mantle and (b) core phases are plotted for an epicentral distance of 29°. (c) Travel time curves of corresponding seismic phases in (a) and (b) within a distance range of 0–95°. The light blue shaded region highlights the distance range of 27°–40° where most marsquakes locate.

2.3 Synthetic waveforms

We use the spectral element code AxiSEM [7] to compute synthetic seismograms within the distance range of 3°–40° for models AK_mean, SKS_GD, and MSL_IPGP shown in Supplementary Fig. 1. Owing to a lack of accurate locations and mechanisms for marsquakes [8-21], we assume an explosion source at a depth of 33 km. The simulation is conducted with a maximum frequency of 0.5 Hz, and the length of the calculated seismogram is set to be 3,000 s. The attenuation is also considered with the $Q\kappa$ being a constant value of 100,000. Meanwhile, the $Q\mu$ value is 100 in the crust, 300 within a depth less than 200 km, and 600 in the deeper structure [1]. As shown in Supplementary Fig. 3, within the distance range of 27°–40°, where most detected marsquakes locate, the core phases introduced in Supplementary Fig. 1 are distinctly visible. Notably, the P′P′ phases have robust energies and appear as distinct and separated arrivals within a long-time window. In contrast, the core-reflected PcP intersects the strong S and S multiples at the distance range of 27°–40° (Supplementary Fig. 3), making the identification of PcP difficult. To reduce the effect of the Martian crustal reverberations for a stable array analysis later, we simply remove the crust and replace it with the topmost mantle, which generates synthetics with much cleaner core phases and their associated depth phases (Supplementary Fig. 4). Furthermore, we observe that the P′P′r for the models of SKS_GD and MSL_IPGP are considerably stronger than those for the model AK_mean within the distance range of 27°–40°.

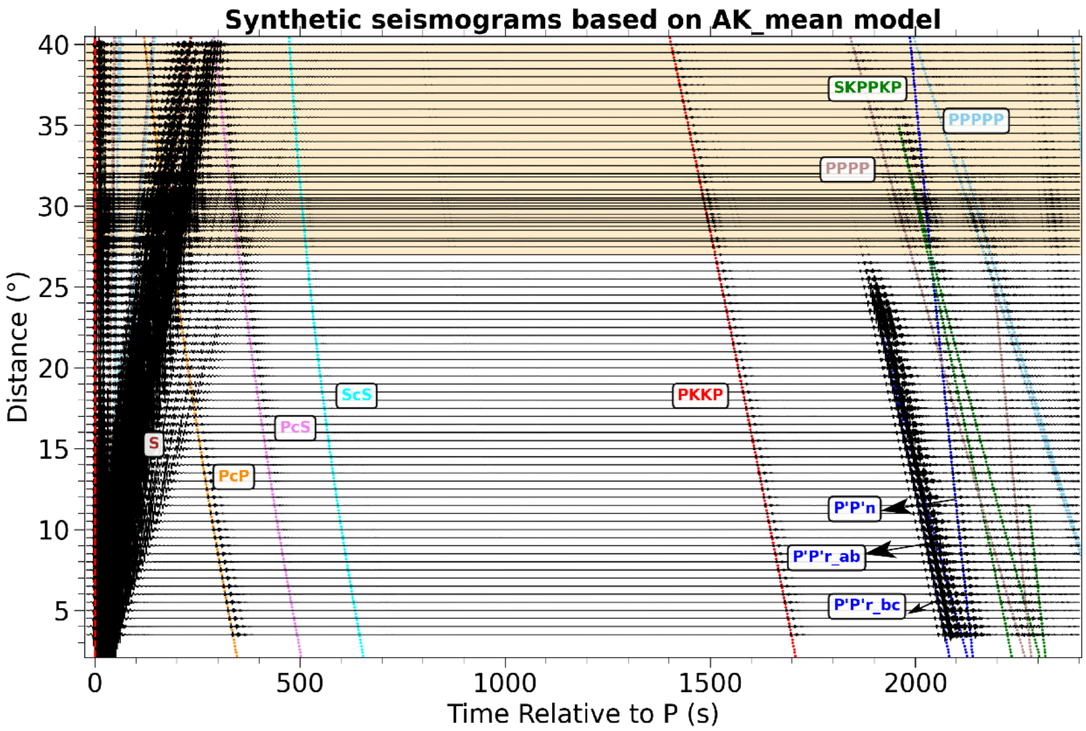

Supplementary Fig. 3. Synthetic seismograms generated with the AK_mean model. Colored dotted lines represent the theoretical travel-times of different seismic phases relative to the direct P as annotated with corresponding texts. The yellow shaded region outlines the epicentral distance range of 27°–40°.

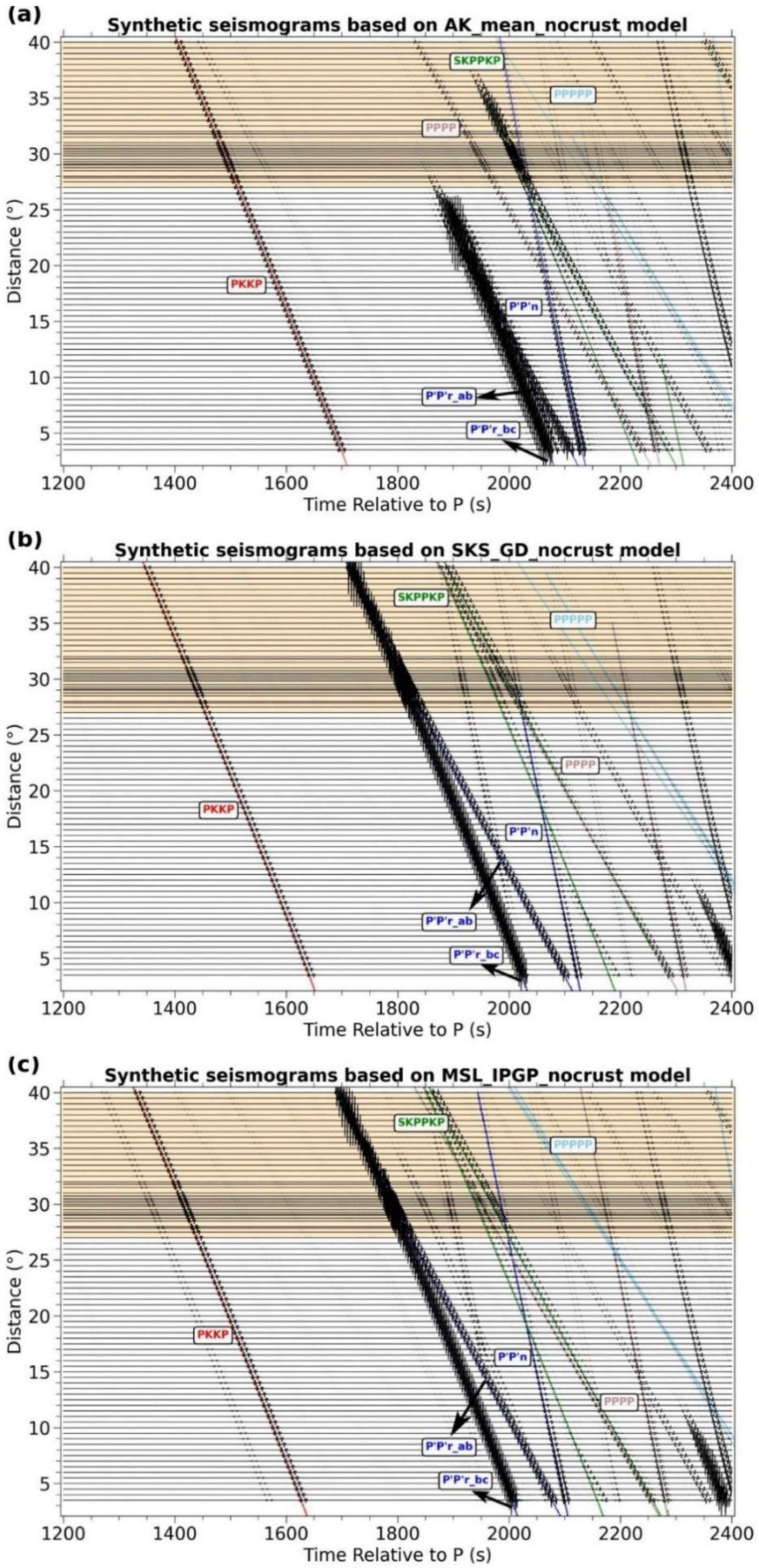

Supplementary Fig. 4. Synthetic seismograms using different velocity models by removing the crust. (a) AK_mean model, (b) SKS_GD model, and (c) MSL_IPGP model. Colored dotted lines represent the theoretical travel-times of different seismic phases relative to the direct P as annotated with corresponding texts.

2.4 Vespagram of the synthetics

A detailed description of the method of generating vespagram is provided in **Methods** (in the main text). In this section, we briefly present the vespagrams of the synthetics generated for the models of SKS_GD without crust in Supplementary Fig. 4 to highlight the robustness of the targeted core phases, i.e., PKKP and P′P′. It is important to emphasize that we exclusively utilize synthetic traces within the epicentral distance range of 27°–40°.

Supplementary Fig. 5 display the vespagrams for mantle and core phases. A strong energy on the vespagram indicates a coherent arrival with specified travel time and slowness, corresponding to a distinct seismic phase. In the vespagrams, the mantle phases, PPPP and PPPPP, and the core phases can be well separated by their distinct slownesses. We notice that, for the SKS_GD model, both P′P′r_ab and P′P′r_bc phases are well separated on the vespagram, although the energy of P′P′r_ab is weaker.

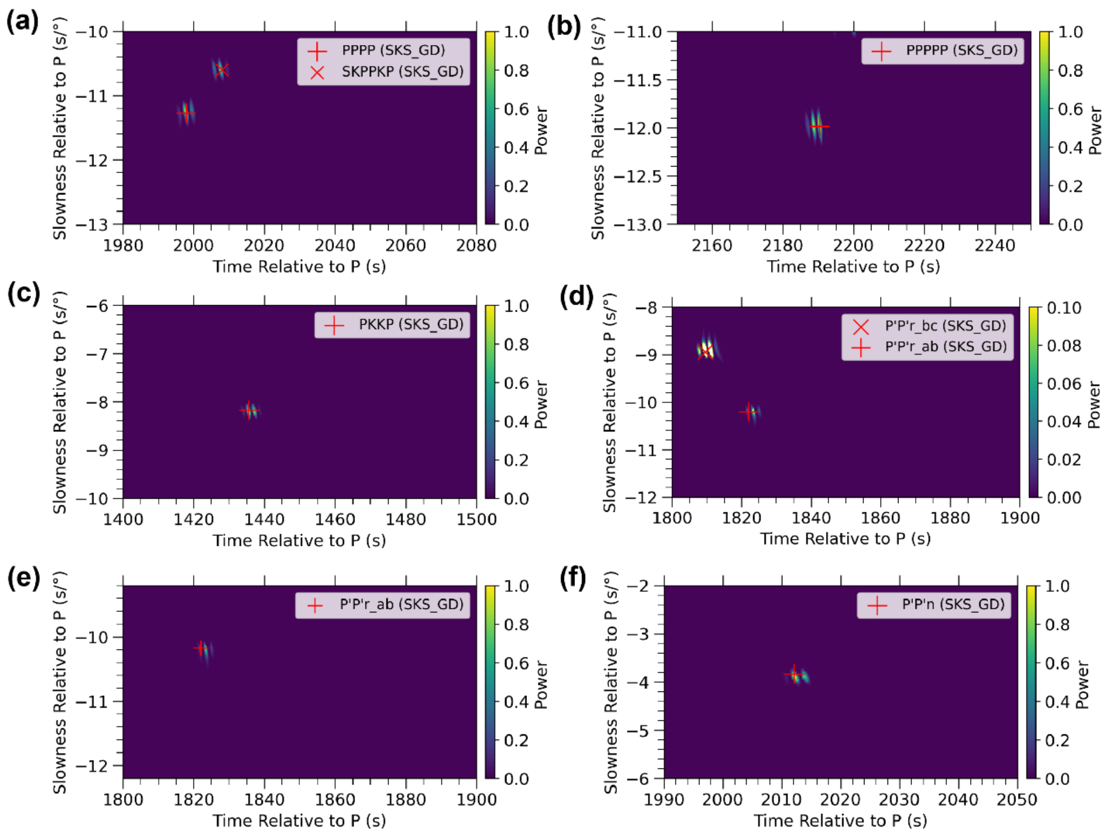

Supplementary Fig. 5. Vespagram analysis for mantle phases (PPPP and PPPPP) along the major arc (a-b), and for core phases PKKP (c), P′P′r (d-e), and P′P′n (f) for the SKS_GD model. Red cross and plus symbols highlight the slowness and travel-time measurements of these phases relative to the P at the reference distance of 29°, using the corresponding seismic velocity model. In each panel, the stacking energy is normalized on the corresponding maximum, respectively. In (d), we set a different color scale to highlight the P′P′r_ab due to its weaker amplitude.

2.5 The effect of uncertainty in source depth

Due to the limitations of a single seismometer and the uncertainty in picking depth phases, the depth of the marsquake is less well resolved [9-13, 15, 18, 19, 22-26]. To estimate the effect of uncertainty in source depth, we use the SKS_GD model to compute the travel times and slownesses of core phases at a distance of 29° for different source depths. As shown in Supplementary Fig. 6, varying the source depth from 0 to 60 km only produce small differences in both travel times (~2 s) and slownesses (< ~0.1 s/°) relative to the direct P. Thus, the influence of uncertainty in source depths can be ignored, especially when considering the much larger uncertainties associated with picking travel times. Therefore, here we assume that variations in the depths of marsquakes, provided they are shallower than 60 km, have negligible effects on our vespagram results when using the direct P as the reference phase.

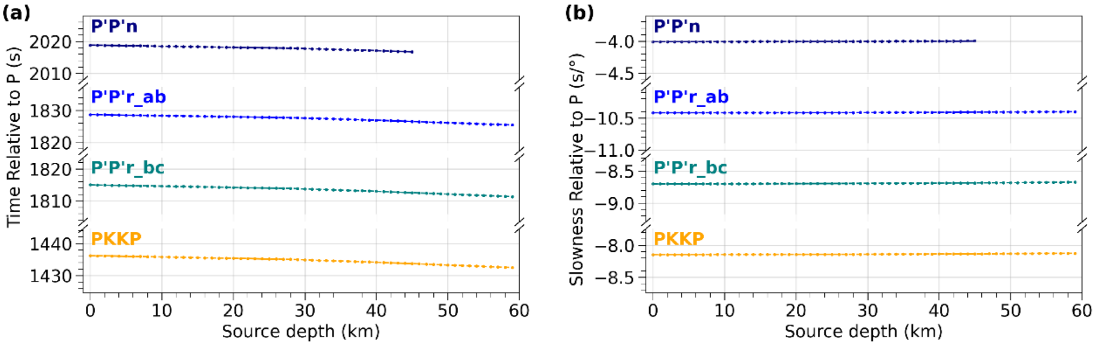

Supplementary Fig. 6. Effects of different source depths. (a) Travel times and (b) slownesses of core phases relative to the P. The travel times and slownesses of core phases are computed at a constant distance of 29° for the SKS_GD velocity model.

3 Data process and core phases detection

3.1 Data

The Marsquake Service (MQS; [27]) compiled a catalog of 1,323 seismic signals [28] within the dataset [14, 29]. Among these marsquakes, 98 events belong to the low-frequency (LF) event family, showing energy predominantly below 1 Hz, and 36 LF events are characterized as quality A or B by having clear P- and S-arrivals. For our analysis, we select 23 LF marsquakes with quality A or B within an epicentral distance range of 27°–40° (Extended Data Table 1), which allows us to perform array analysis and detect possible core phases. Note that most of these events are concentrated at an epicentral distance of 30° (Supplementary Fig. 7). An additional set of 3 LF events at greater distances are further used for the validation of our inverted core model.

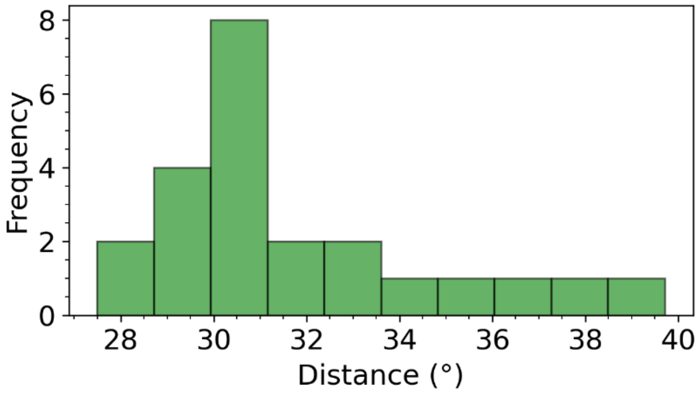

Supplementary Fig. 7. Histogram of epicentral distance distribution for the used 23 LF events.

3.2 Phase detection process

3.2.1 Summary of phase detection process

On the Earth, we can acquire extensive seismic data from earthquakes of considerable magnitude and develop diverse methodologies to investigate core-transiting phases, such as P′P′ and PKKP [30, 31]. This is made possible by the dense network of stations that spans almost the entire global land [32-34]. However, the challenge arises on Mars when dealing with a single station and a limited number of marsquakes, which are characterized by small magnitude and low signal-to-noise ratio (SNR). Detecting core phases on individual events with a single method becomes particularly demanding under these conditions. Consequently, we adopt a two-stage approach to identify potential core-transiting phases in the data collected by the InSight seismometer.

The first stage is to acquire slant stacking energy by employing vespagram analysis on all 23 selected LF events (**Methods**). To ensure the robustness of the coherent energy peak on the vespagram, different types of bootstrap resampling tests (detailed in **Methods**) are implemented. In the second stage, guided by the results from the vespagram analysis, we apply complementary approaches, including time-domain envelopes (filter bank) and polarized analysis (**Methods**), to identify core phases for individual events.

3.2.2 Stage I: Vespagram analysis

3.2.2.1 Feasibility of vespagram analysis of polarization filtered waveforms

Vespagram analysis of waveform data provides important information on the amplitude and polarity of targeted seismic phases. However, uncertainties in the marsquakes source mechanisms and possible three-dimensional structures make it challenging to identify seismic phases using source array vespagram analysis based solely on stacking waveforms. In Supplementary Fig. 8, we display an example of synthetic vespagrams for the direct P phase, generated with different focal mechanisms, source durations (ranging from 1 to 4 s), and depths. In compared to the vespagram using envelopes, which shows a strong energy peak (Supplementary Fig. 8b), the

vespagram based waveform produces scattered energy, even when applying the phase weight stack (PWS) method (Supplementary Fig. 8c-d). This suggests that, for weak core phases, stacking envelope is more effective. Nevertheless, we also perform array analysis on the waveform data to extract amplitude and polarity information for PKiKP and PPKP (**Methods and Section 3.4**), which cannot be obtained from envelope stacking alone.

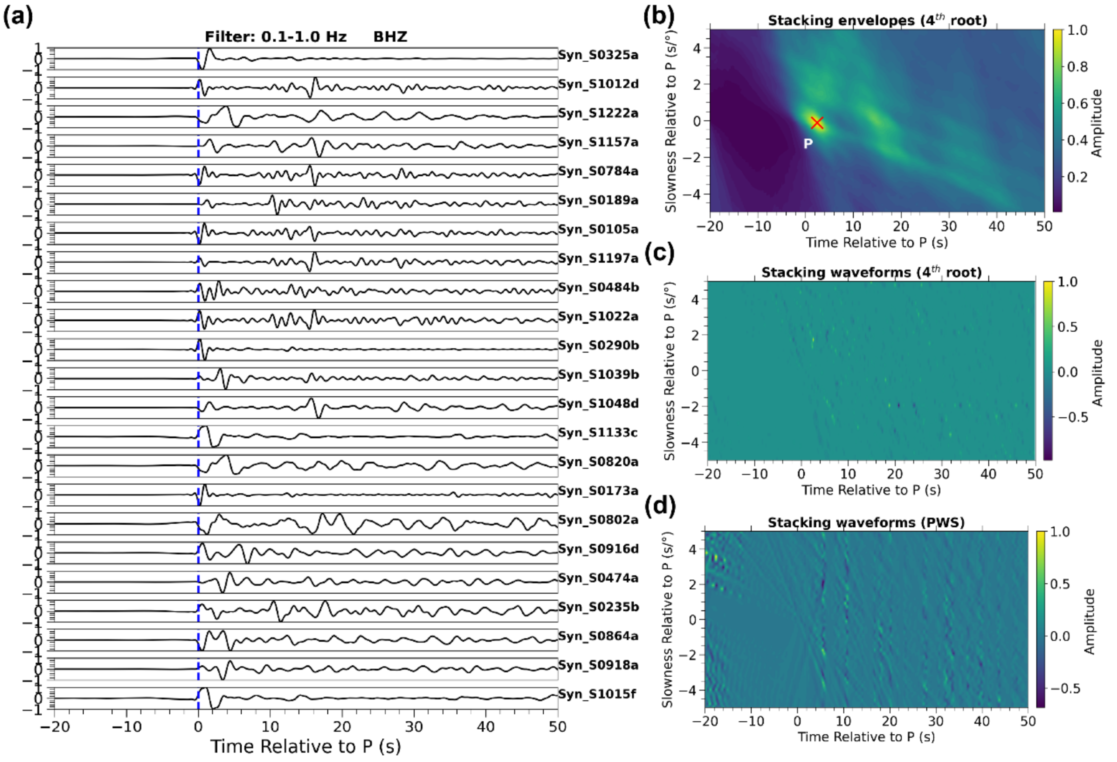

Supplementary Fig. 8. Comparison of array analysis using waveforms and envelopes. (a) Synthetic P-wave waveforms on the vertical component. Each trace is bandpass filtered into 0.1-1.0 Hz. The blue dashed lines mark the arrivals of P-wave. (b) Vespagram analysis using the envelopes, with the 4th- root stacking method applied. The red cross marks the vespagram measurements for the P. (c) Same as (b), but using waveforms. (d) Same as (c), but applying the phase-weighted stack (PWS) method.

3.2.2.1.1 Mantle phases P, S, ScS, PPPP and PPPPP

Compared to the Martian core, the mantle structure is better constrained owing to a greater number of travel time measurements for mantle seismic phases sampling different depths [1, 2, 35-37]. Thus, we first perform the vespagram analysis with mantle phases to ensure the reliability of slant stacking on Mars data characterized by low SNR.

Supplementary Fig. 9 displays the vespagrams for compression waves on Z component (P, PPPP and PPPPP), and shear waves on tangential (T) component (S and ScS), using 23 events (Extended Data Table 1). Coherent energies with strong amplitude emerge in the target time-slowness windows, indicating stable phase identifications. For PP and PPP, it is difficult to identify them on vespagram (Supplementary Fig. 9a), as they follow the P-wave and may be mixed with depth phase and multiple reflections within the crust. For major arc PPPP and PPPPP, there is time

difference up to ~60 s and slowness difference up to ~2.5 s/° between model predictions (Supplementary Fig. 9b-c) depending on models, which may be arose from the uncertainties of the mantle velocity models and a heterogeneous Martian mantle. The prominent arrival of ScS on the vespagrams in a large time-slowness window underscore that the vespagram can be diagnostic for detecting seismic phases with low amplitude here.

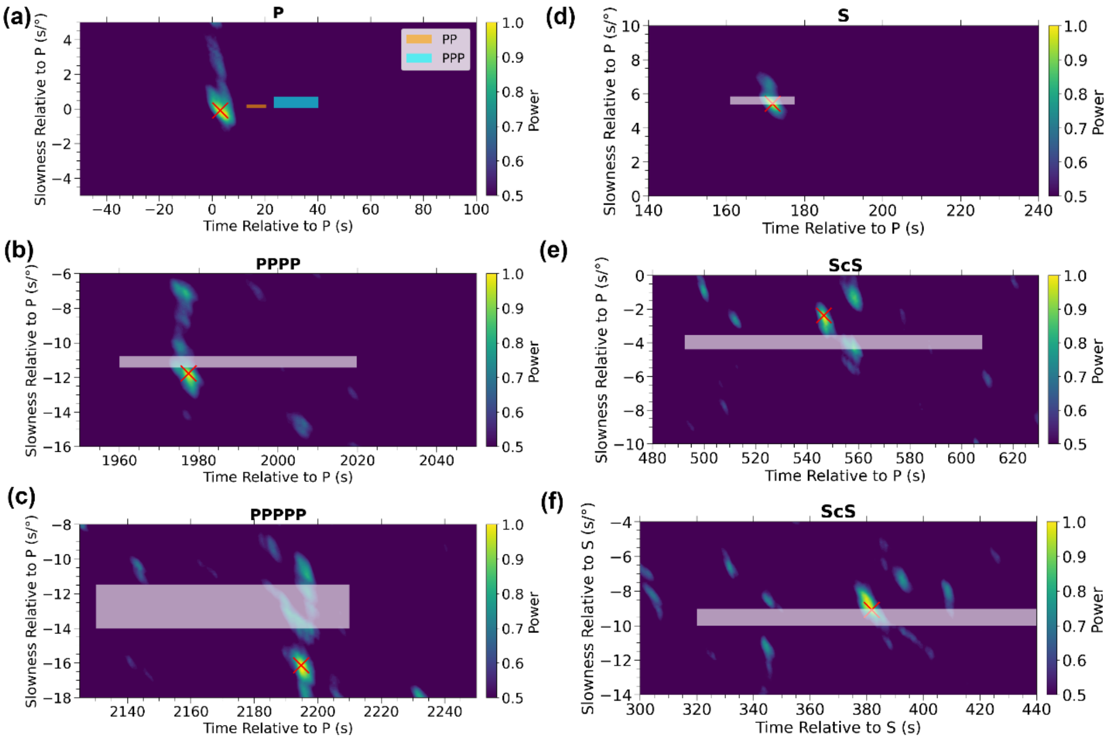

Supplementary Fig. 9. Vespagram analysis for Martian mantle phases. (a) P, (b) PPPP, (c) PPPPP, (d) S and (e-f) ScS. The red cross marks the slowness and travel time measurements with the highest coherent energy in the vespagram. The orange and cyan shaded rectangles in (a) denote the predicted slowness and travel time ranges for PP and PPP, respectively, calculated at the reference distance of 29° and a source depth of 33 km based on available seismic velocity models. In (b)-(e), white shaded region denotes the predicted range for PPPP, PPPPP, S, and ScS relative to P, respectively. The analysis for ScS is performed on the tangential component. (f) Same as (e), but showing the vespagram analysis for ScS relative to S.

3.2.2.1.2 P′P′r_ab and P′P′n

In the vespagram for P′P′r_ab (Supplementary Fig. 10a), two distinct coherent energy peaks are observed at (1,820 s, -10 s/°) and (1,835 s, -12 s/°), which are both close to the predicted values for the P′P′ phase. Another energy peak is at (1,835 s, -6 s/°) but with a much higher slowness than model predictions. Thus, we exclude this peak from further analysis. Similarly, the vespagram for the P'P'n phase also show multiple peaks (Supplementary Fig. 10b). We focus on the peak with the largest amplitude at (2010 s, -5 s/°), which has a slowness slightly lower than the model predictions. This difference may be caused by a heterogeneous Martian mantle.

Synthetic results reveal that the behavior of the P′P′ phase can vary significantly depending on the velocity structure. For instance, P′P′r_ab is visible on synthetic waveforms at distances up to 40° for the SKS_GD and MSL_IPGP models (Supplementary Fig. 4b-c), but is only visible at distances less than 32° for the AK_mean model (Supplementary Fig. 4a). Therefore, regardless of which model is

correct, using small distance data could enhance the stacking energy and make the phase detection more robust. Consequently, for the P′P′ phase, we generate an additional vespagram by excluding the furthest 5 events (S0784a, S1157a, S1222a, S1012d and S0325a). As shown in Supplementary Fig. 10c-d, the vespagrams of P′P′r_ab and P′P′n both show the highest coherent energies consistent with the results of using all events stacking, which also demonstrates the reliability of the P′P′ phase detected by the array analysis. The presence of distinct coherent energy peaks near the model predictions strengths the reliability of phase detection through vespagram analysis. To further address uncertainties and evaluate the robustness of the vespagram result, we employ bootstrap resampling tests, as detailed in **Section 3.2.2.2**.

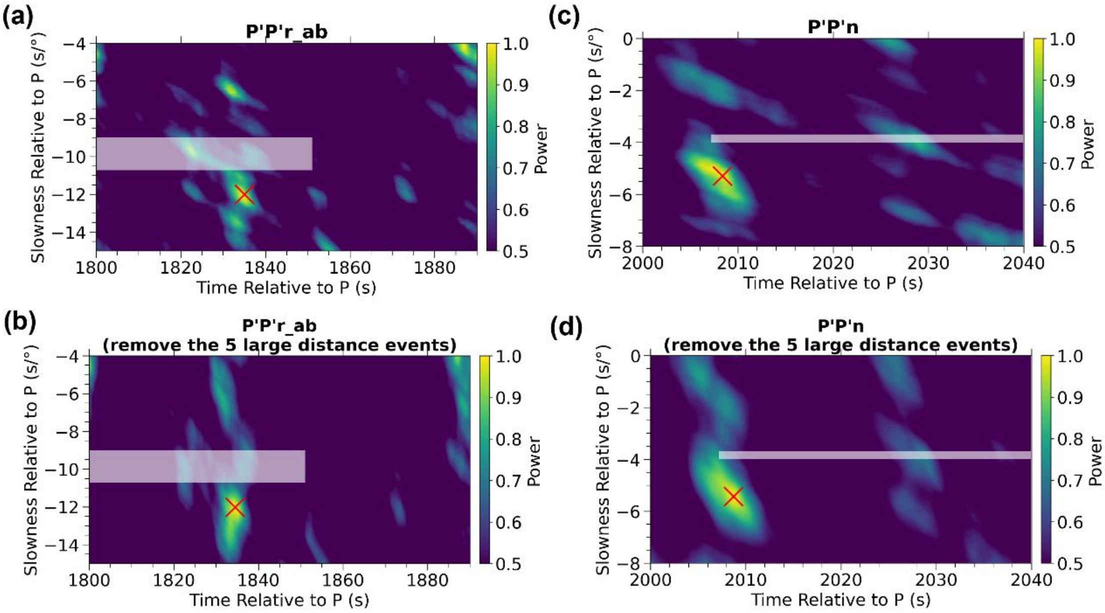

Supplementary Fig. 10. Vespagram analysis for P′P′ phase using different events. Vespagram analysis for (a) P′P′r_ab and (b) P′P′n using all 23 events. In (c)-(d), 18 events, excluding S0784a, S1157a, S1222a, S1012d, and S0325a are included in the vespagram. Notably, compared to the P'P'n, the P'P'r_ab samples deeper part of the core and its uncertainties in slowness are much larger. This indicates that existing models differ most significantly in the deep core (Supplementary Fig. 1).

3.2.2.1.3 PKKP

Compared to P′P′, PKKP could sample the very center of the core at a radius of ~270 km for a pure liquid core (Supplementary Fig. 2b). However, existing core velocity models are primarily constrained by the travel times of SKS [2], which only sample the shallow part of the core, with a radius larger than ~750 km. Therefore, the deeper Martian core structure has a large uncertainty, making the search for PKKP difficult. Thus, we extend our search to a much longer time window for vespagram analysis (Supplementary Fig. 11a). It is noticed that two obvious coherent energies, with the slowness of the predicted PKKP (-7.0 ~ -9.0 s/°), appear at 1,290 s, and 1,340 s, both preceding the model predictions significantly. Moreover, their similar slownesses indicate that both arrivals should be related to core phases with rays transiting the deep core as the PKKP. Supplementary Fig. 11b displays six events on which both signals are simultaneously visible. Polarization analysis reveals that their high vertical-horizontal summed FDPA intensity (VRM-HRM) (see **Methods** for more

details), further provides additional support for their potential identification as PKKP phases.

It is worth noting that Khan et al. (2023) and Samuel et al. (2023) propose the existence of a molten silicate layer (MSL) [3, 4], with a thickness of ~150 km, overlying the liquid core, which reconciles the conflict arising from the large size and low density of the Martian core. With the presence of the MSL, two phases are expected to reflect both at the CMB ($PKKP_{CMB}$) and the top of the MSL layer ($PKKP_{MSL}$) (Supplementary Fig. 12a). For the MSL_IPGP model (Supplementary Fig. 1) [4], within the distance range of 27°–40°, both phases are distinguishable on the vespagram (Supplementary Fig. 12d), and the $PKKP_{CMB}$ is ~60 s earlier than the $PKKP_{MSL}$. Therefore, as for the observations in Supplementary Fig. 11, if the ~1,290 s arrival corresponds to the $PKKP_{CMB}$, the arrival at ~1,340 s might be associated with the $PKKP_{MSL}$.

Regardless of both cases, the arrival of PKKP occurs at least ~50 s earlier than the predictions, suggesting a considerably faster core toward the center. If a pure liquid core is assumed, explaining such a fast PKKP requires a velocity gradient of about ~0.0025 1/s steeper in the central 880 km of the core compared to the shallow core (Supplementary Fig. 13). However, achieving such a pronounced velocity gradient toward the center may also be challenging for a purely liquid core [38-45]. Alternatively, introducing a solid inner core at the center provides a more straightforward explanation for our observations.

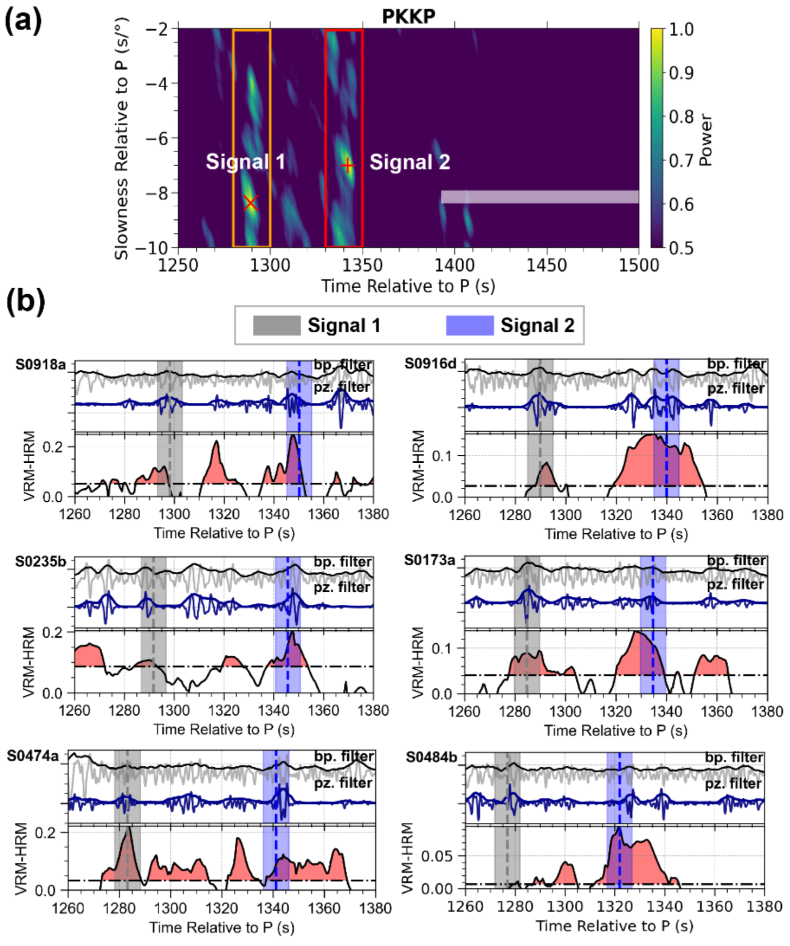

Supplementary Fig. 11. Identification of candidate two PKKP arrivals in individual events. (a) Vespagram analysis for PKKP using all 23 events, showing two coherent energies at ~1,290 s

(“Signal 1”; red cross) and ~1,340 s (“Signal 2”; red plus). (b) Polarization analysis of six events confirm the identification of the two signals in (a). In each panel: (top) bandpass- (grey line), (middle) polarization-filtered (blue line) waveforms with envelopes, and (bottom) vertical-horizontal summed FDPA intensity (VRM-HRM). Grey and blue dashed lines indicate the predicted travel times for the two PKKP phases at their respective slownesses from (a), with ±5 s uncertainties denoted by shaded regions. Horizontal dash-dotted line marks the mean value of VRM-HRM in the 100 s window preceding PKKP (“Signal 2”).

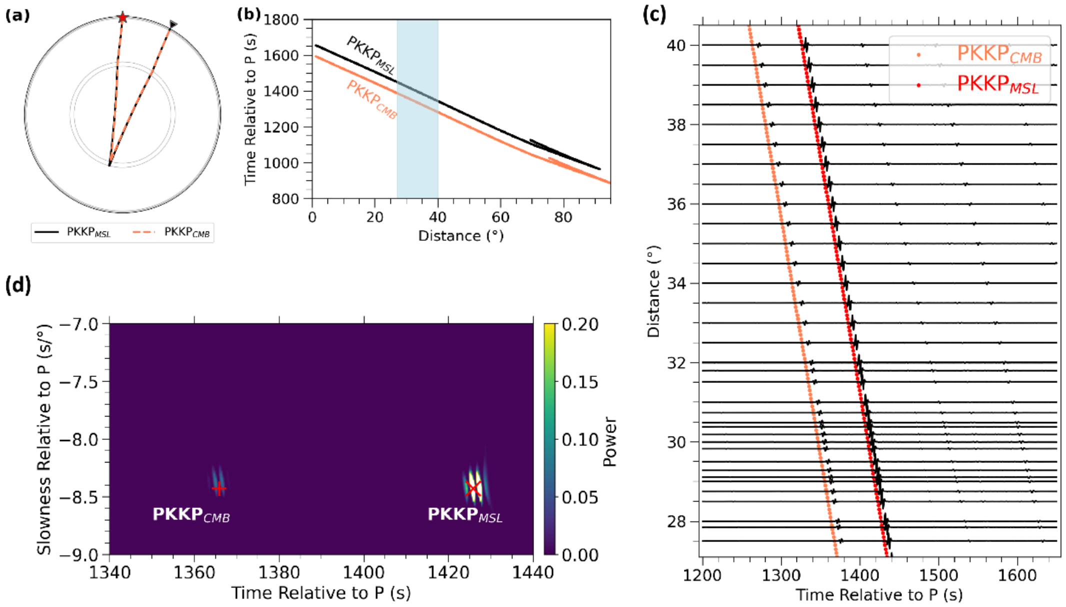

Supplementary Fig. 12. Synthetics of PKKP phase for the MSL_IPGP model. (a) Ray path and (b) travel time curves for $PKKP_{MSL}$ and $PKKP_{CMB}$, which are reflected from the top of molten silicate layer (MSL) and CMB, respectively. Here, the depth of the marsquake is set at 33 km. (c) Synthetic seismograms of $PKKP_{MSL}$ and $PKKP_{CMB}$ within a distance range of 27°–40°. (d) Vespagram analysis for $PKKP_{MSL}$ and $PKKP_{CMB}$, using the synthetics in (c).

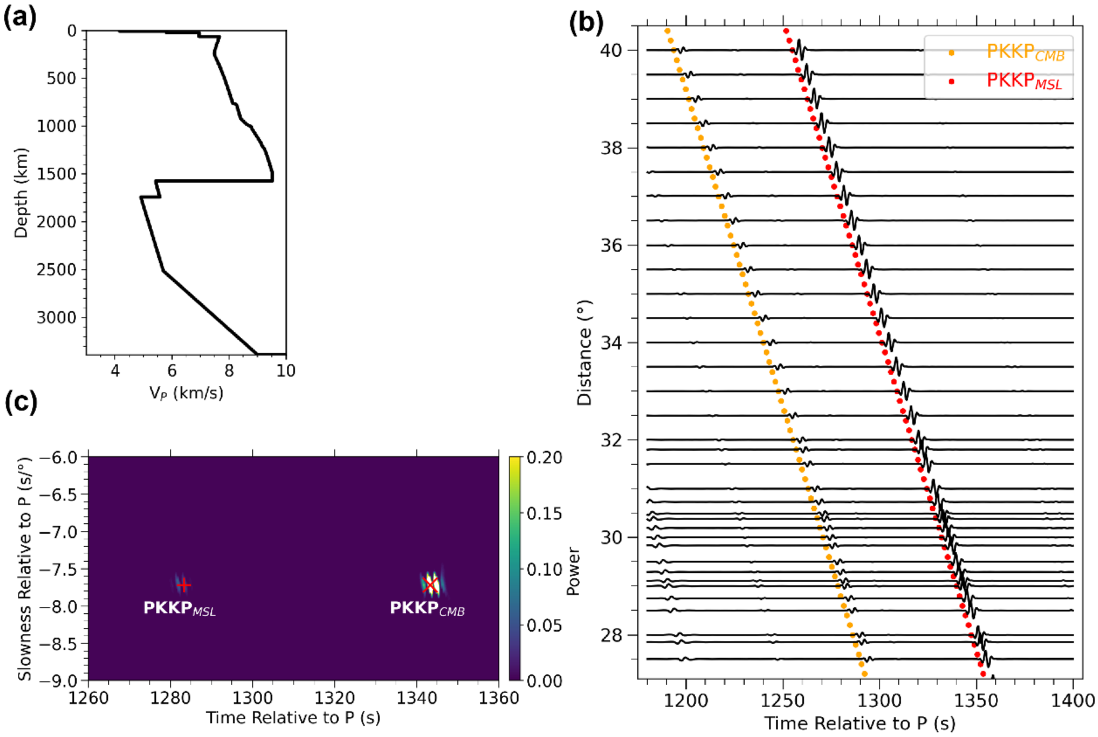

Supplementary Fig. 13. Synthetics for a model with a much steeper velocity gradient in the deep

core. In (a), the velocity gradient for the deep core is steeper ~0.0025 1/s than that of the MSL_IPGP model (Supplementary Fig. 1), aligning with the much faster PKKP observed in Supplementary Fig. 11. (b) Synthetic waveforms within a distance range of 27°–40° with a source depth of 33 km. (c) Vespagram analysis using the synthetics in (b).

3.2.2.2 Assessment of vespagram result robustness

To better accommodate the effects from data selection and possible location errors, we conduct bootstrap resampling tests (**Methods** and Supplementary Fig. 14). The results for P′P′r_ab are shown in Supplementary Fig. 15. Type I (randomly selecting two-thirds of the events), Type II (randomly selecting half of the events within a distance range of 29°–32°) and Type III (randomly shifting each trace within a range of -10 and 10 s before stacking) tests all give similar results, with noticeable coherent energy peaks at ~1,835 s relative to the P, although the slowness is somewhat inconsistent. These results confirm that the identification of the P′P′r_ab is robust.

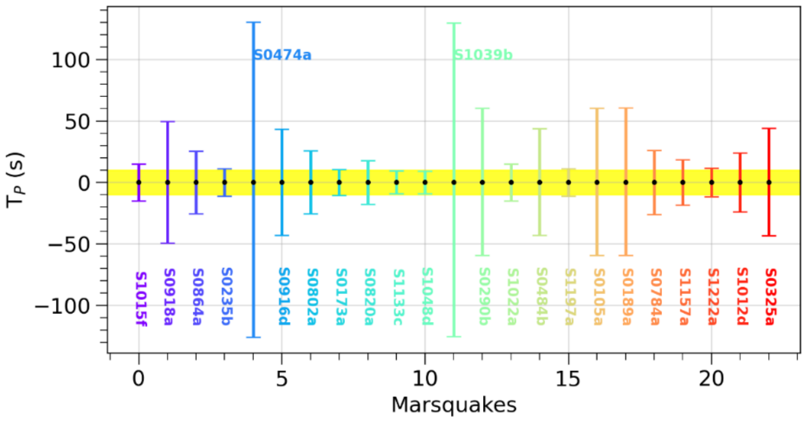

Supplementary Fig. 14. P-arrival time and uncertainty distribution for 23 events in vespagram analysis. The epicentral distances and uncertainties (1-σ) for events (Extended Data Table 1) are provided by the MQS in the V14 catalog [28]. The uncertainty of the P-arrival time for each event due to the uncertainty of the epicentral distance are calculated based on the SKS_GD model. The yellow region denotes a range of ±10 s uncertainties of the P-arrival time.

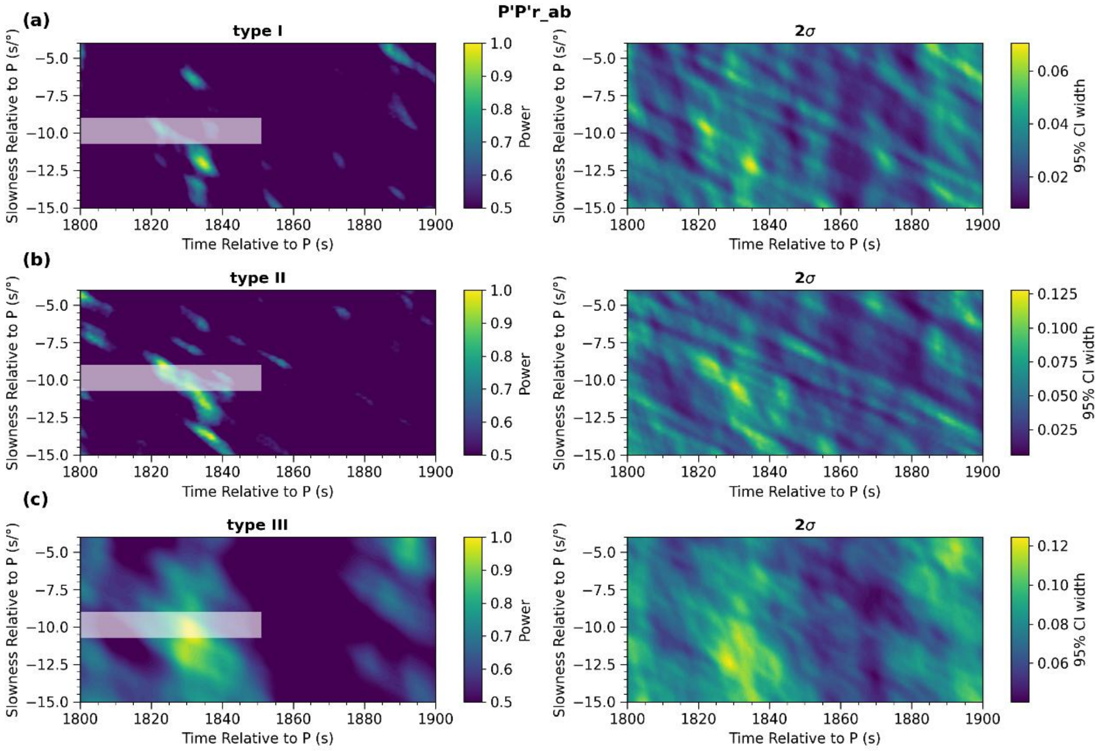

Supplementary Fig. 15. Bootstrap resampling tests for P′P′r_ab. The mean vespagram in (a) is derived from 200 iterations of slant stacking by randomly resampling two-thirds of all 23 events. The 2σ plot in (a) shows the corresponding width of a 95% confidence interval. The white shaded region denotes the predicted slowness and travel time range of P′P′r_ab at the reference distance of 29° using available seismic velocity models. (b) is the same as (a), but it involves randomly selecting half of the seismic events within the 29°–32° distance range, along with events at other distances (Extended Data Table 1). (c) is the same as (a) but for bootstrap resampling with a random shift of ±10 s for each trace before stacking.

3.2.2.3 Uncertainties from vespagram analysis

To better constrain the phase arrival time, slowness, and the associated uncertainties, we employ the type I bootstrap resampling test (**Methods**), in which two-thirds of the 23 events in Extended Data Table 1 are randomly selected to generate a vespagram. In each vespagram, points with energy greater than a specified threshold of the peak are assigned a value of 1, while the remaining points are assigned a value of 0. Repeating this process 10,000 times allows us to create a cumulative distribution for each point and compute the mean and standard deviation values for travel time and slowness, respectively. We use the PKKP phase to describe our threshold selection procedure (Supplementary Fig. 16). First, we examine the energy distributions within signal and selected noise windows, derived from the type I bootstrap resampling test (Supplementary Fig. 16a). Then, we evaluate three candidate thresholds based on the maximum energy: 50% (below the noise level), 70% (just above the noise level), and 85% (approximately the first quartile of the signal distribution) (Supplementary Fig. 16b). While lower thresholds increase detection frequency, they also result in scattered energy distributions (Supplementary Fig. 16c). To balance robustness and precision, we select 85% as the optimal threshold. This criterion is also applied to the identification of other core phases. Supplementary Fig. 17 gives an example of P′P′ phase, and

Supplementary Table 1 lists the results for other core phases.

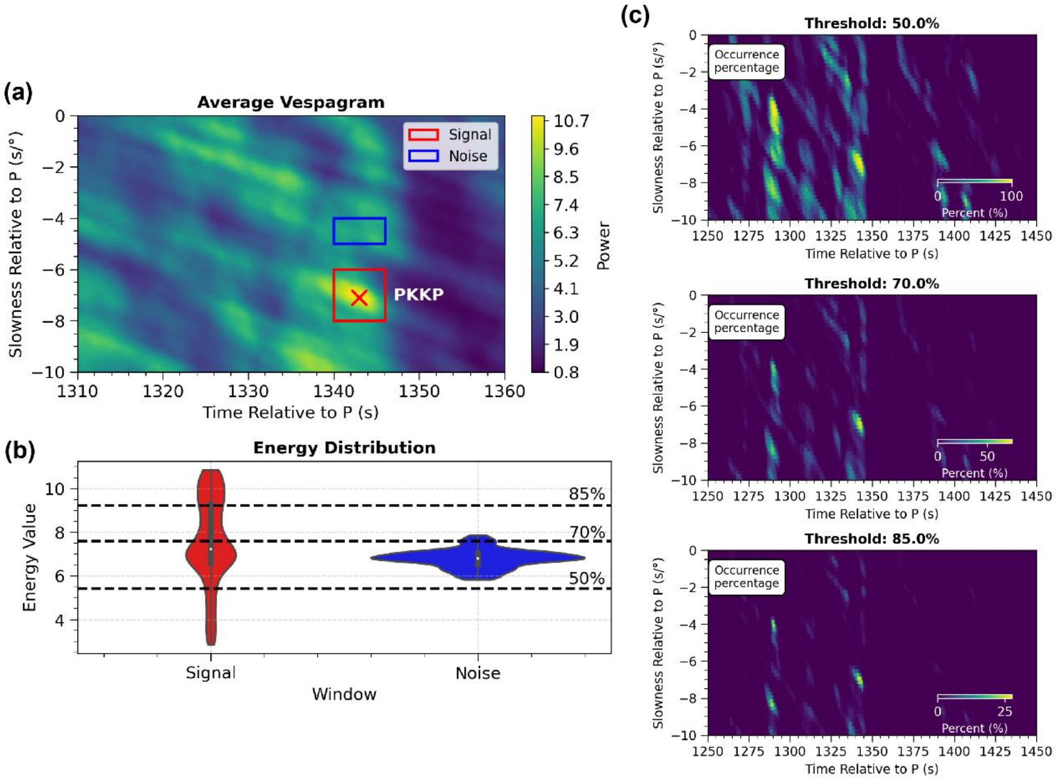

Supplementary Fig. 16. Threshold sensitivity tests. (a) Average vespagram from 200 iterations of slant stacking by randomly resampling two-thirds of all 23 events (type I bootstrap resampling test). Red and blue rectangles mark the signal and selected noise window, respectively. (b) Energy distributions within the signal (red) and noise (blue) windows in (a) across all 200 stacks. Violin plots show kernel density estimates, with white dots indicating medians and gray rectangles spanning the 1st to 3rd quartiles. Black dashed lines mark candidate thresholds at 50%, 70%, and 85% of the maximum energy. (c) Occurrence percentages of the PKKP phase for each threshold (top to bottom: 50%, 70%, 85%).

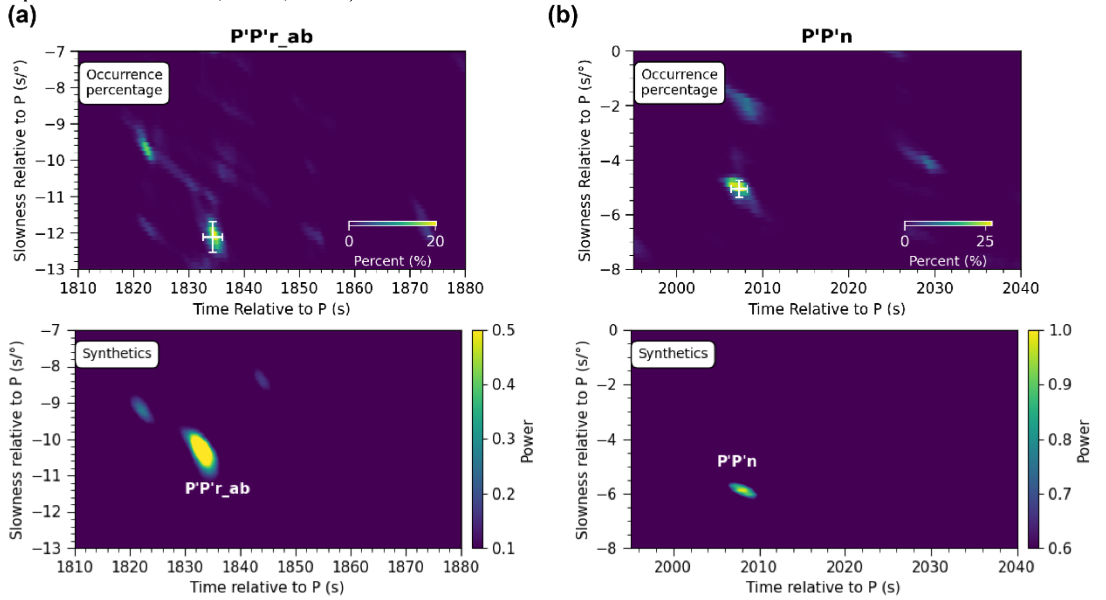

Supplementary Fig. 17. Uncertainty analysis and synthetics for P′P′ phase. (a) From up to bottom, uncertainty analysis for P′P′r_ab phase and vespagram analysis phase using synthetics, generated by the inverted BS_SKS_GD_IC model (case 8 in Extended Data Table 2), respectively. The upper

panel shows the occurrence percentage of grids with energy exceeding than 85% of the peak in each resampling test. White cross indicates the identification of P′P′r_ab at 1835 s, including error bars calculated by fitting its distribution with Gaussian functions (**Methods**). (b) Same as (a), but for P′P′n phase.

Supplementary Table 1. Travel time and slowness relative to P of four core phases based on vespagram analysis.

Phases	T_{phase}-T_P (s)	Slowness relative to P (s/°)
PKKP	1341±5	-7.0±0.4
P′P′r_ab	1835±4	-12.1±0.7
P′P′n	2008±3	-5.0±0.6
PKiKP	604±2	-6.5±0.6

3.2.3 Stage II: Analysis for individual events

As mentioned in **Methods**, we apply two complementary approaches to identify core phases for individual events. Here, we present an example of detecting P′P′r_ab on event S1048d (Supplementary Figs. 18-20). Additional examples are provided in **Supplementary Information B**.

As shown in Supplementary Fig. 2b, the core phases within the distance range of 27°–40° exhibit nearly vertical incidence, exception for P′P′n. Therefore, the inclination should fall approximately within the range of 70°–85° in the time-frequency window around the phase arrival. In addition, the back azimuth information in the same window should ideally align with the event location, allowing for an ambiguity of 180°. Given the considerable uncertainty in back azimuth estimates for marsquakes, we slightly relax this criterion for core phase arrival picking. To quantitatively assess the inclination and azimuth information, we compute the probability density for selected time-frequency windows by marginalizing over the frequency and time axes. The initial time window is set to be ±8 s around the phase arrivals, and the selection of frequency band between 0.1–2 Hz varies depending on events. Thus, we initially determine the dominant frequency band by polarized analysis of direct P-waves for each used event, and the detailed results are listed in Supplementary Table 2. Subsequently, these frequency bands are used to compute the probability density for our target core phases (Supplementary Tabs. 3-6). It is important to note that the frequency bands do not always align with P due to varying noise levels.

Furthermore, we also apply principal component analysis (PCA) [46] to measure the inclination in a ±2.5 s time window around the phase arrivals. Through linear fitting of the particle motion on Vertical(Z)-Radial(R) components, we can obtain the incident angle and its associated error in the vertical plane.

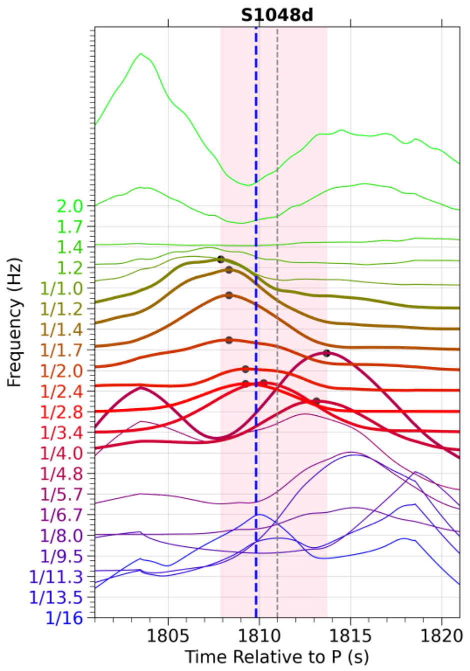

Supplementary Fig. 18. Example of filter bank analysis for P′P′r_ab of event S1048d. The colored waveforms represent filtered data with narrow bandpass filters with different center frequencies, as indicated by the colored text. The gray dashed line denotes the travel time picks computed by vespagram analysis and the blue dashed line denotes the root-mean-square value of time-domain envelope peaks (black circles) in a frequency range from 1/4.8 Hz to 1/1.2 Hz (heavy colored lines). The pink shaded region outlines the time distribution of the peaks.

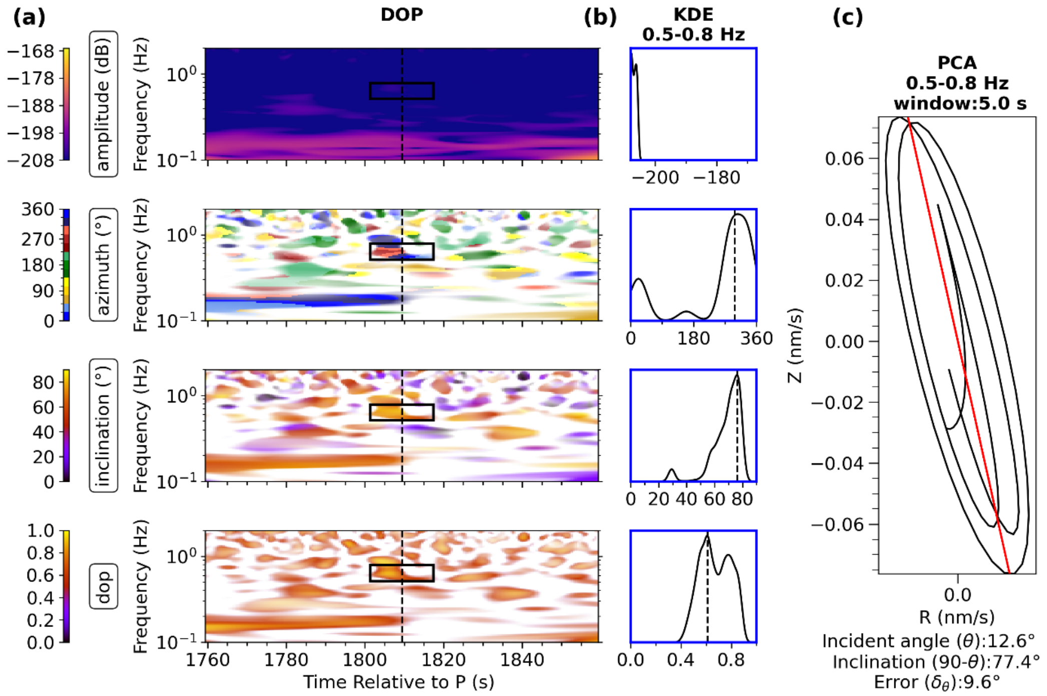

Supplementary Fig. 19. Example of polarization analysis on event S1048d for P′P′r_ab phase. (a) From top to bottom, spectrum, azimuth, inclination angle of the major axis of particle motion, and ellipticity (DOP) obtained from polarization analysis. We reject all parts of the signal with a DOP < 0.6 by setting the corresponding part of the S-transformed data to zero, which allowed us to suppress some weakly polarized signals. The inclination angle represents the angle deviating from the horizontal plane. The black dashed lines denote the travel-time picks obtained from filter bank analysis. The black rectangles outline the travel-time pick uncertainties of ±8 s in the frequency

range 0.5-0.8 Hz. (b) Kernel density estimation. The black dashed lines denote the maximum values of probability density computed by marginalizing over the frequency and time axes as the black rectangle shown in (a). (c) The particle motions on the vertical and radial components in a time window of ±2.5 s based on the travel time picked from filter bank analysis. The red line denotes the fitted line and its incident angle deviating from the vertical plane and error are annotated below.

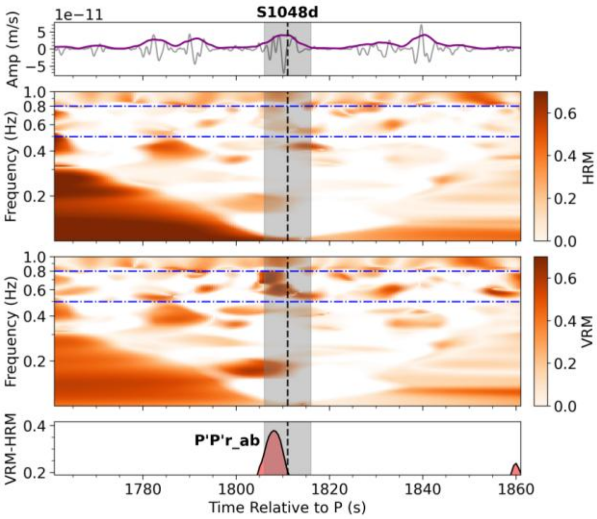

Supplementary Fig. 20. Example of frequency dependent polarization analysis (FDPA) on S1048d event for P′P′r_ab. Top row: Polarization filtered waveform and envelope on vertical component. Second row: Horizontal component of FDPA analysis (0.1–1.0 Hz). Third row: Vertical component of FDPA analysis. Bottom row: Averaged excess linearly polarized energy across 0.5–0.8 Hz (blue dotted lines in second and third panels). In each panel, the black dashed line denotes the travel time picked from filter bank analysis with an uncertainty of ±5 s, outlined by the gray shaded region.

3.3 Identification of PKiKP phase

As discussed in **Section 3.2.2.1.3**, the observed PKKP arrives significantly earlier than predicted, suggesting the presence of a much faster core toward the center. It is difficult to explain this phenomenon with a purely liquid core featuring a much stronger velocity gradient toward the center [38, 39, 41, 42, 47, 48]. Alternatively, having a solid inner-core (IC) with a higher velocity provides an easy and logical explanation. If an IC indeed exists, the PKiKP phase, reflection at the inner-core boundary (ICB), should be presented, which in turn provides the most direct and definitive evidence supporting the presence of IC. Thus, to explore this hypothesis, based on the SKS_GD model and assuming the P-wave velocity in IC to be 8.0 km/s, we test different IC radii and compute the theoretical relative slowness of PKiKP to P. Supplementary Fig. 21 displays the relative slowness range (-7.4 ~ -6.4 s/°) for PKiKP, considering an IC radius ranging from 0 to 900 km. Then, we find that an IC size of 600 km can approximately fit the PKKP arrival time. We further employ AxiSEM [7] to compute the synthetic waveforms for this model, which are used to perform vespagram analysis to assess the visibility of PKiKP within the distance range of 27°–40°. As shown in Supplementary Fig. 22, assuming an IC size of 600 km yields a prominent coherent energy at ~600 s relative to the P, with a relative slowness of ~-6.7 s/° (Supplementary Fig. 22c).

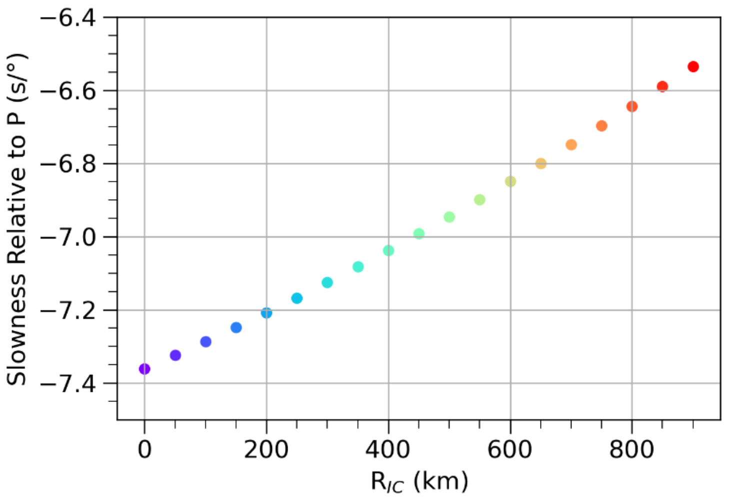

Supplementary Fig. 21. Relative slowness to P of PKiKP for different IC radii. Assuming that the velocity of the mantle and OC matches that of the SKS_GD model, while the velocity within the IC remains constant at 8.0 km/s, we can produce a range of velocity models by varying the radii of the IC. Subsequently, we calculate the relative slowness of PKiKP using TauP [6].

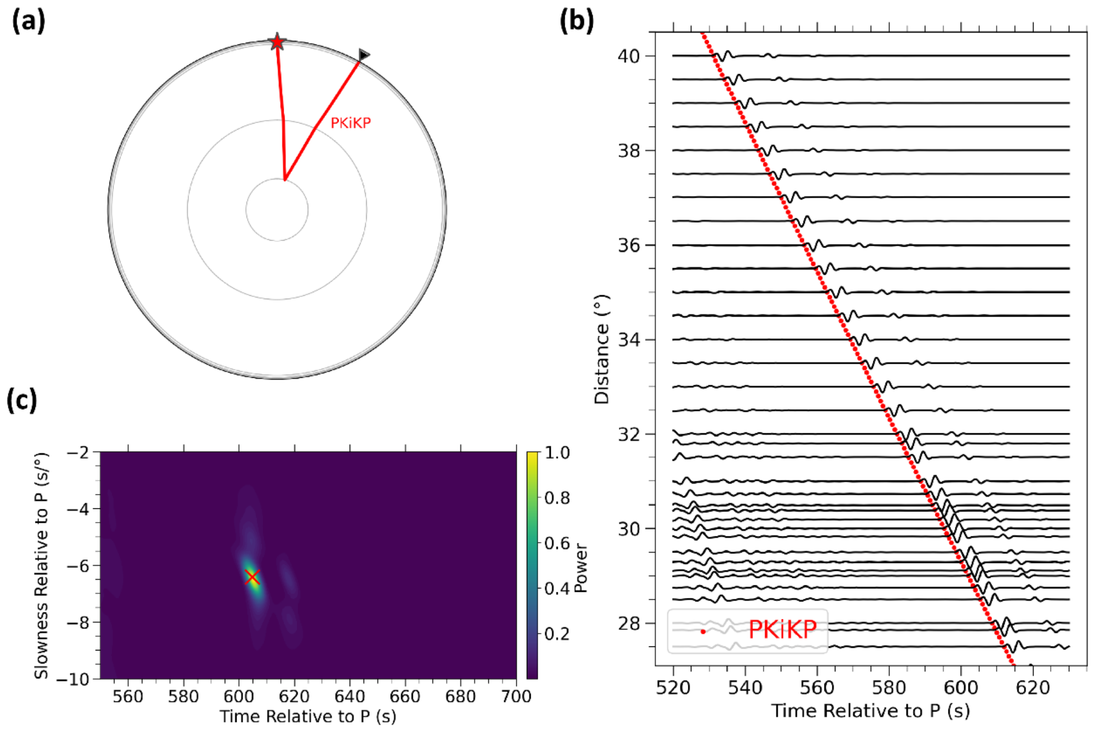

Supplementary Fig. 22. Synthetics for PKiKP assuming an IC size of 600 km. (a) Ray path of PKiKP at a distance of 29°. (b) Synthetic seismograms of PKiKP within a distance range of 27°–40°. (c) Vespagram analysis using the synthetics in (b).

Following the detection procedure for P′P′ and PKKP, we first try to identify PKiKP through vespagram analysis using 23 events within the distance range of 27°–40°. As shown in Supplementary Fig. 23a, a coherent energy peak at ~604 s and -7.0 ± 0.3 s/° affirms the identification of PKiKP. Stacking both the raw data and the glitch-only data, following Kim et al. (2021) [49], further validate that this signal is not an artifact caused by glitches (Supplementary Fig. 23).

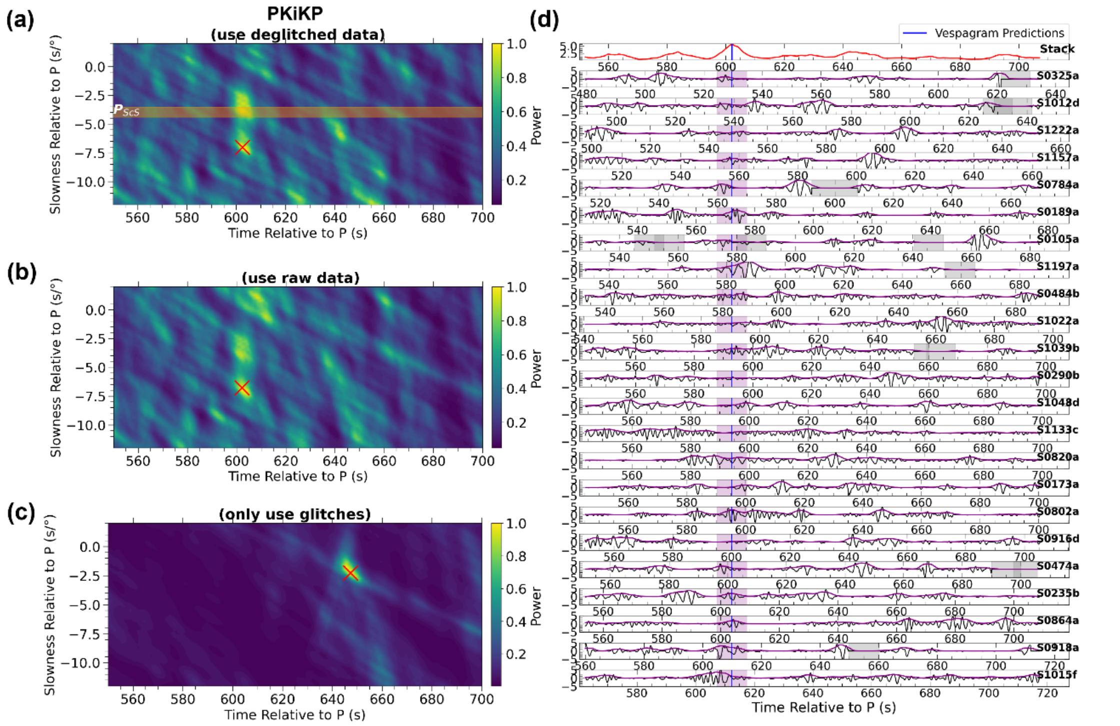

Supplementary Fig. 23. Effects of glitches on vespagram analysis. Vespagram analysis for PKiKP using (a) deglitched data, (b) raw data, and (c) detected glitches. The red crosses highlight the slowness and travel-time measurements with the highest coherent energy. The orange shaded region in (a) denotes the theoretical slowness range for ScS relative to P, based on available seismic models. (d) Polarization filtered waveforms after deglitching and the corresponding envelopes used as input for the vespagram analysis. Blue lines mark the calculated travel times of PKiKP using results from the vespagram analysis in (a), with an uncertainty of ±5 s shown by purple shaded region. The red trace in the top panel shows the envelope stack. The gray shaded regions indicate the presence of known instrument glitches.

It is worth noting that the signal observed at 604 s and -4 s/° may correspond to the ScS phase plus a top-side reflection at a mantle interface, given the similar slowness. To test this hypothesis, we multiply polarity correction factors to the three-component waveforms before stacking, accounting for the polarity difference between compressional and shear waves: the VRM-HRM factor is used to enhance vertically polarized energy, while the HRM-VRM factor amplifies horizontally polarized energy (**Methods**). This processing significantly strengthens both PKiKP on the Z-component (Supplementary Fig. 24f) and ScS on the T-component (Supplementary Fig. 24g), compared to the original vespagram analysis (Supplementary Fig. 24a-c). Although the energy at (604 s, -4 s/°) weakens on the Z-component (Supplementary Fig. 24f), it remains a relatively strong arrival, suggesting an incident P wave at the station. Thus, we generated synthetics incorporating a low-velocity zone (LVZ) with δVp = -10% and δVs = -15% at 150 km depth. While this model produces a weak ScSS150P (ScS plus a segment of S-to-P reflection at the top of the LVZ), the amplitude is insufficient to match observations (Supplementary Fig. 25a). Interestingly, a stronger S-to-S reflection (ScSS150S) is generated, potentially linked to those slightly delayed energies at -4 s/° on the horizontal components (Supplementary Fig. 24d-e). These results suggest a localized strong LVZ could potentially introduce additional signals near PKiKP. Furthermore, uncertainties in marsquake locations may also contribute. Synthetic tests introducing random shifts within ±5 s or ±10 s to individual trace prior stacking produce scattered energies with consistent arrival times but varying slownesses (Supplementary

Fig. 25b), indicating that some observed signals near 600 s may originate from marsquake location uncertainties. Nonetheless, PKiKP remains a prominent arrival across all vespagrams. However, given the limited data, we are unable to conclusively determine the origin of the signal at 604 s and -4 s/° at the current stage.

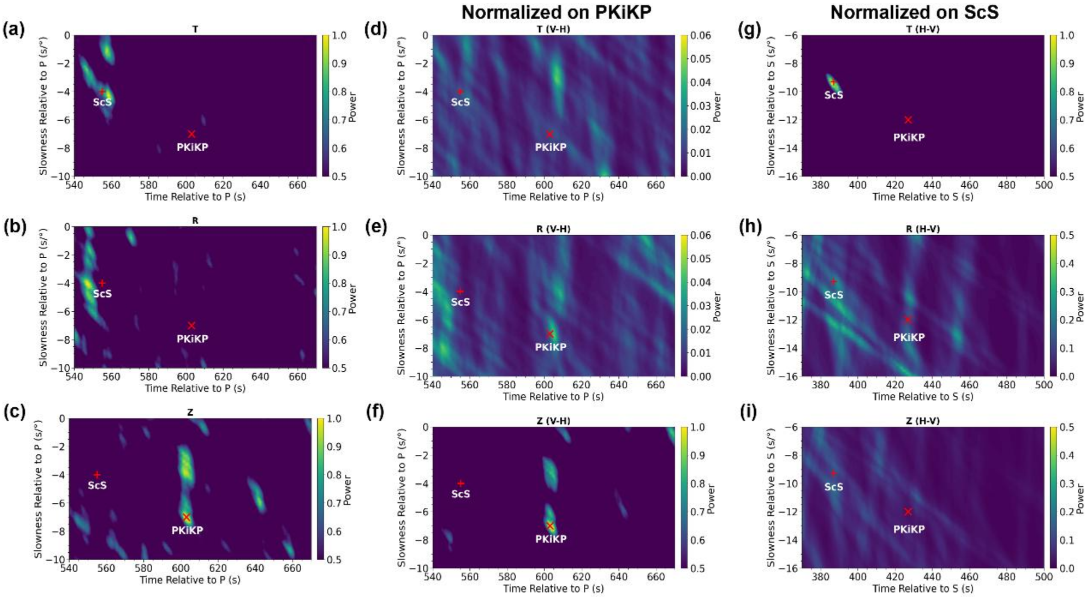

Supplementary Fig. 24. Comparison of vespagram analyses with and without further modification with the difference between vertical rectilinear motion (VRM) and horizontal rectilinear motion (HRM). (a)-(c) Vespagram of PKiKP and ScS on the T, R, and Z components, respectively, without further polarization filtering. (d)-(f) Vespagram on the T, R, Z components aligned on the P arrival after multiplying the VRM–HRM factor. (g-i) Same as (d-f), but aligned on the S arrival and multiplied by the HRM–VRM factor. In panels (d–f), amplitudes are normalized to PKiKP, with adjusted color scales to accommodate low amplitudes on the T and R components. In panels (g–i), amplitudes are normalized to ScS.

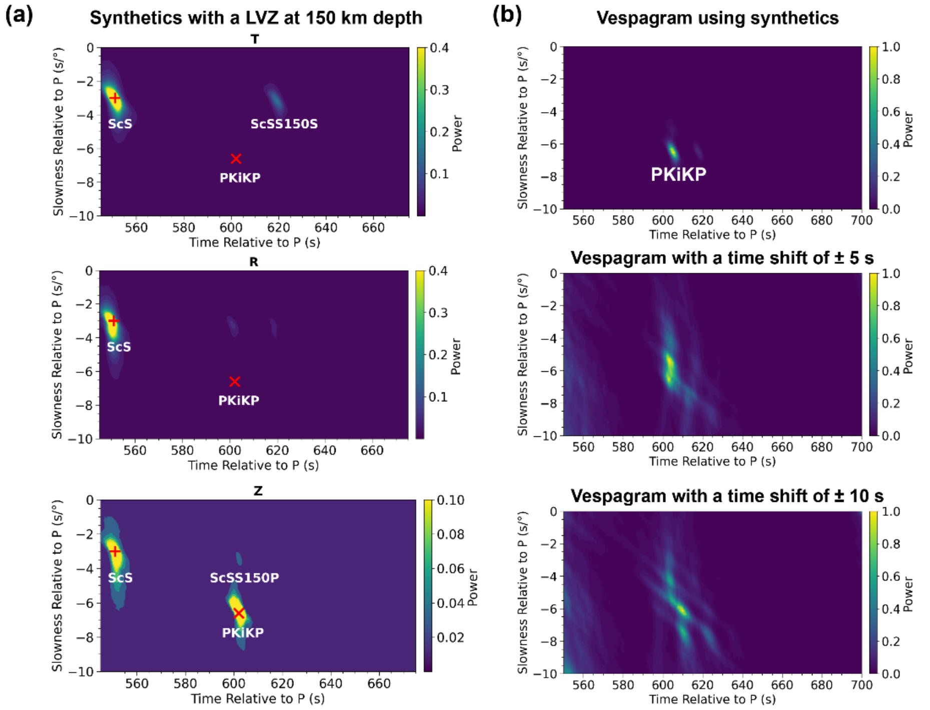

Supplementary Fig. 25. Possible origins of the observed energy at (604 s, −4 s/°) in the PKiKP vespagram. (a) Vespagram analysis on three components using synthetic data for a model with a

low-velocity zone (LVZ) at a depth of 150 km. (a) Vespagram analysis using synthetic data without time shifts (top), and with random time shifts within ±5 s (middle) and ±10 s (bottom) applied to individual traces.

As displayed in Supplementary Fig. 26, all three types of bootstrap resampling tests consistently show a strong energy peak at ~600 s, although the relative slowness in the Type III test is slightly higher at ~-5.0 s/°, compared to ~-6.8 s/° in the Type I and Type II tests. Further uncertainty analysis confirms that the most robust PKiKP arrival occurs at ~604 ± 2 s after P with a relative slowness of -6.5 ± 0.6 s/° (Fig. 2e).

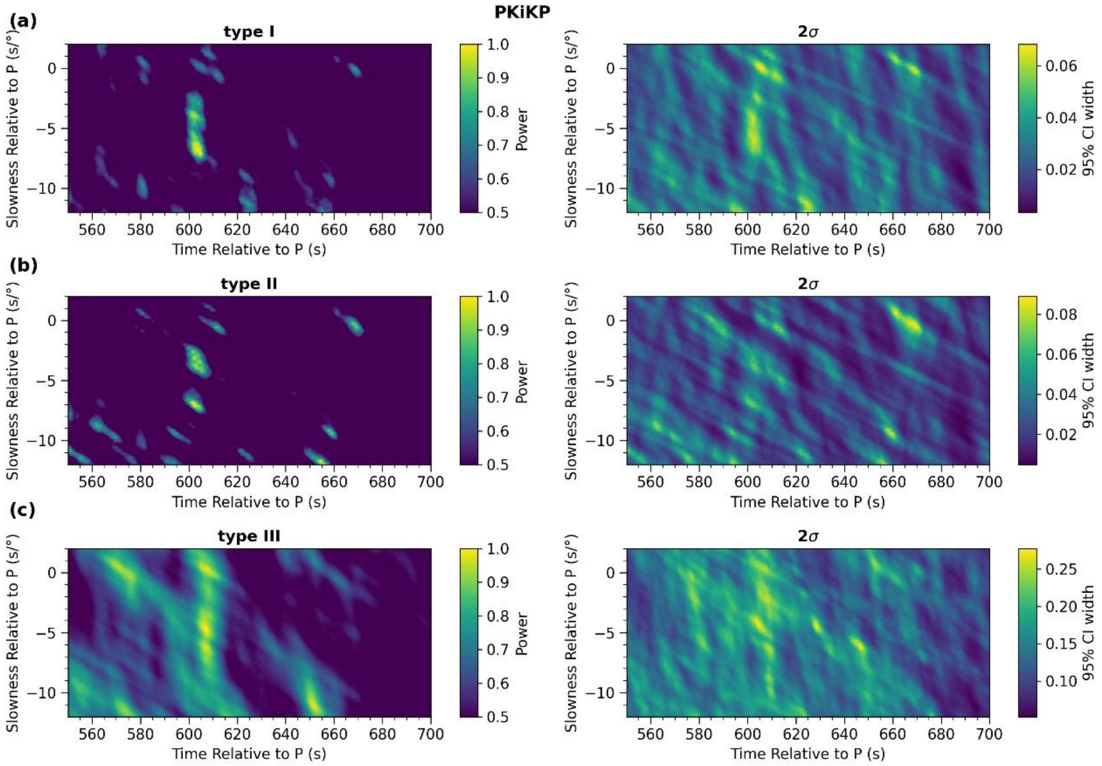

Supplementary Fig. 26. Bootstrap resampling tests for PKiKP. Refer to Supplementary Fig. 15 for a detailed caption**.**

Next, we identify the PKiKP on individual events using same techniques discussed in **Section 3.2.3**, including filter bank and polarized analysis (DOP, PCA and FDPA). Among the 23 events, 7 events show reliable PKiKP phase. Here, for the convenience of viewing, we consolidate the results of all methods into a single figure for each event. Here, we present an example on event S1015f (Supplementary Fig. 27). Additional examples are provided in **Supplementary Information B**.

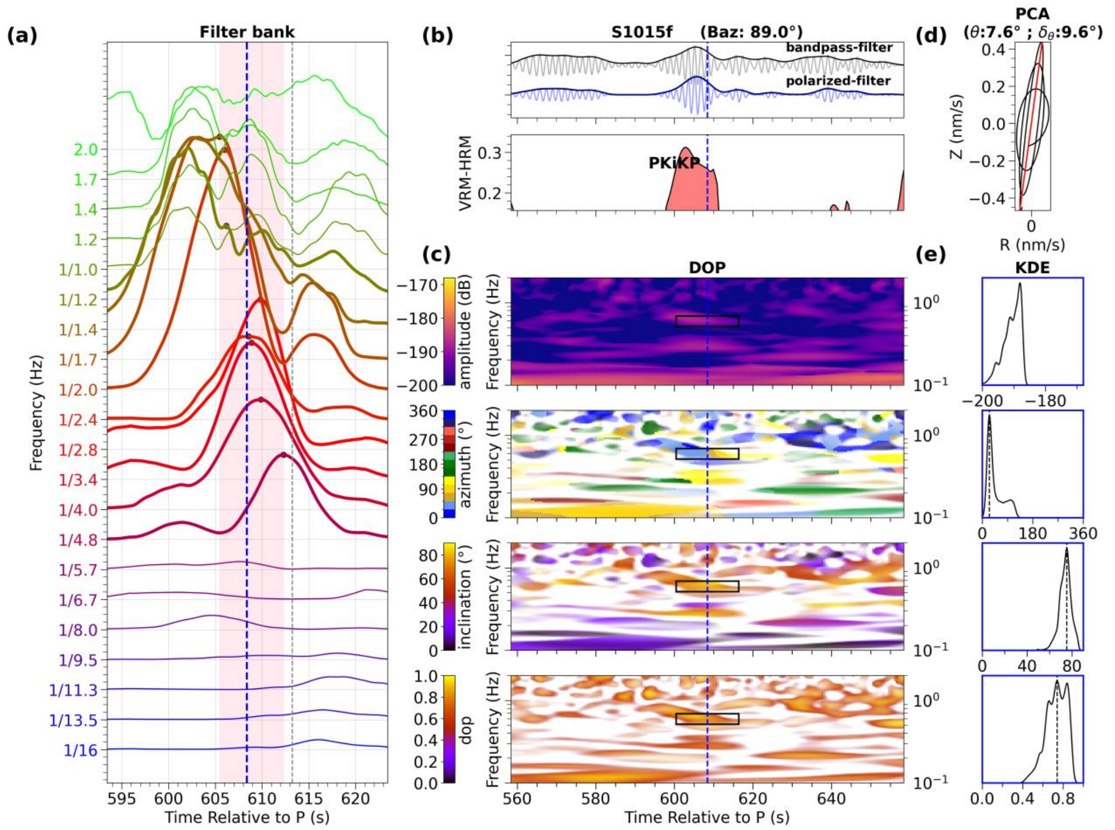

Supplementary Fig. 27. Identification of the PKiKP phase for event S1015f. (a) Filter bank analysis. Refer to Supplementary Fig. 18 for a detailed caption. (b) Waveform and FDPA analysis. The upper panel shows the vertical component waveforms and corresponding envelopes of the bandpass- (grey line) and polarization- (blue line) filtered traces within a frequency band indicated by the black rectangles circled in (c). The back-azimuth is provided by MQS [28]. The lower panel is the vertical-horizontal summed FDPA intensity as a function of time. (c) Polarization analysis. From top to bottom, spectrum, azimuth, inclination angle of the major axis of particle motion, and ellipticity (DOP) obtained from polarization analysis. We reject all parts of the signal with a DOP < 0.6 by setting the corresponding part of the S-transformed data to zero, which allowed us to suppress some weakly polarized signals. The inclination angle represents the angle deviating from the horizontal plane. The black dashed lines denote the travel-time picks obtained from filter bank analysis. The black rectangles outline the travel-time pick uncertainties of ± 8 s in the frequency range 0.5–0.7 Hz. The blue dashed lines in (b) and (c) denote the travel-time picked from filter bank analysis in (a). (d) The particle motions on the vertical and radial components in a time window of ± 2.5 s based on the travel time picked from filter bank analysis. The red line denotes the fitted line and its incident angle deviating from the vertical plane (θ) and error (δ_θ) are annotated below. (e) Kernel density estimation. The black dashed lines denote the maximum values of probability density computed by marginalizing over the frequency and time axes as the black rectangle shown in (c).

3.4 Amplitude and polarity of PKKP and PKiKP

In this section, we present complementary vespagram results of analyzing the amplitude ratio and polarity relationship of core phases using different approaches that are introduced in the **Methods**.

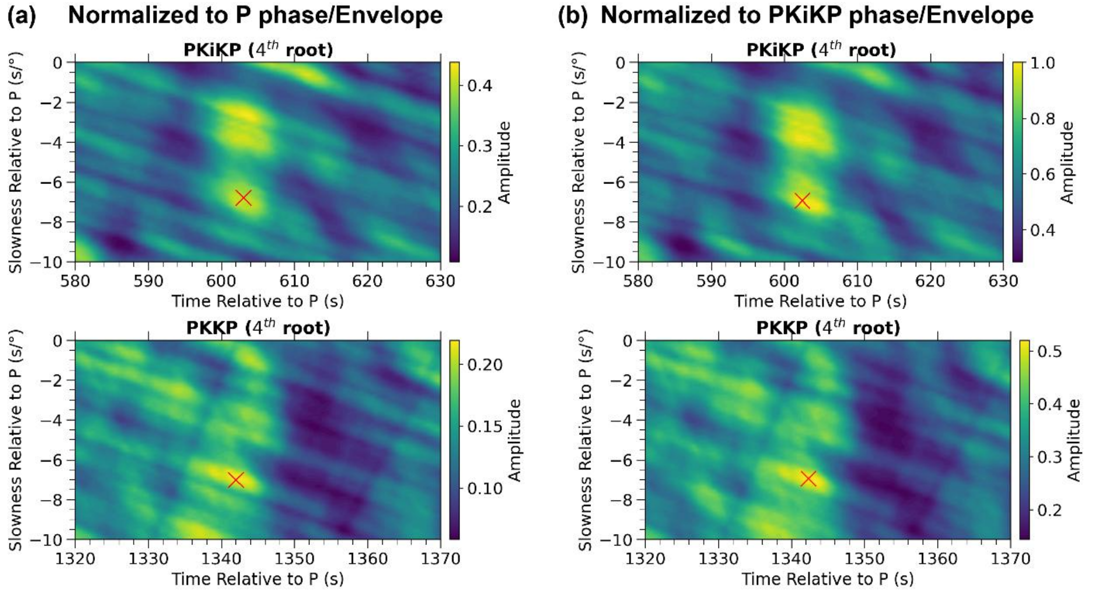

Supplementary Fig. 28. Vespagram analysis of PKiKP and PKKP amplitudes based on envelope. Note that directly using waveforms does not produce coherent energy. Therefore, normalized envelopes of 23 traces are used here to generate the vespagrams. Each trace used in the stacking is normalized to (a) P and (b) PKiKP envelopes. In each panel, the time window for the reference phase's (P or PKiKP) envelope is first normalized using Equation 1 in **Methods**. The envelopes of other phases are then normalized using the same ratio as the reference phase. Red crosses denote the highest coherent energy at the predicted phase arrival time.

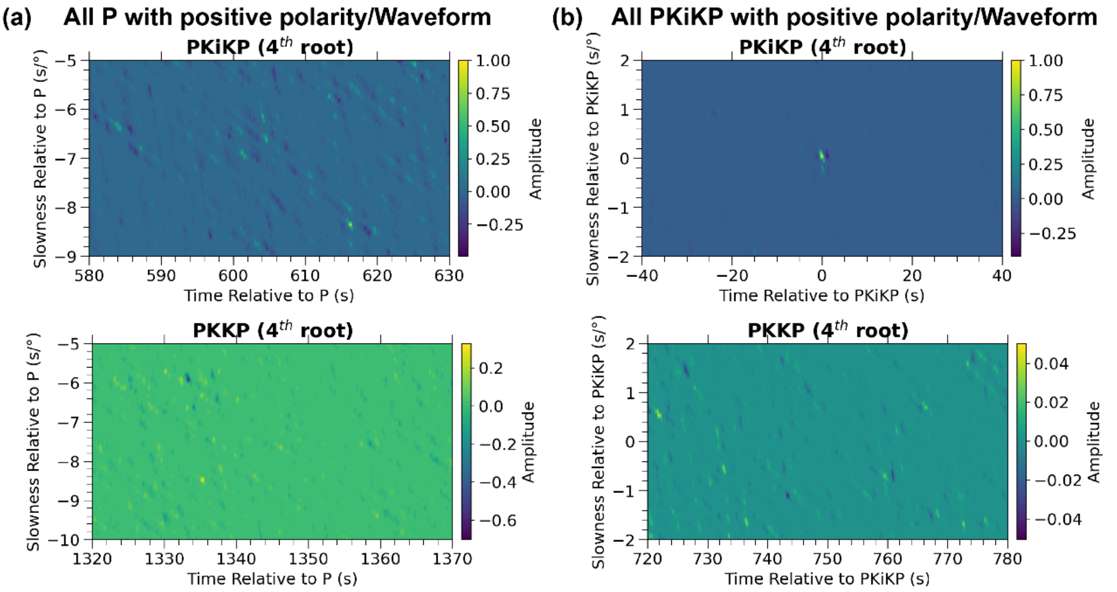

Supplementary Fig. 29. Vespagram analysis of PKKP and PKiKP polarities based on waveform. Before stacking, each trace is adjusted to ensure polarity of (a) P and (b) PKiKP positive and then normalize to the amplitudes PKiKP. Note that for PKKP, we adopt different color scales due to its low amplitude.

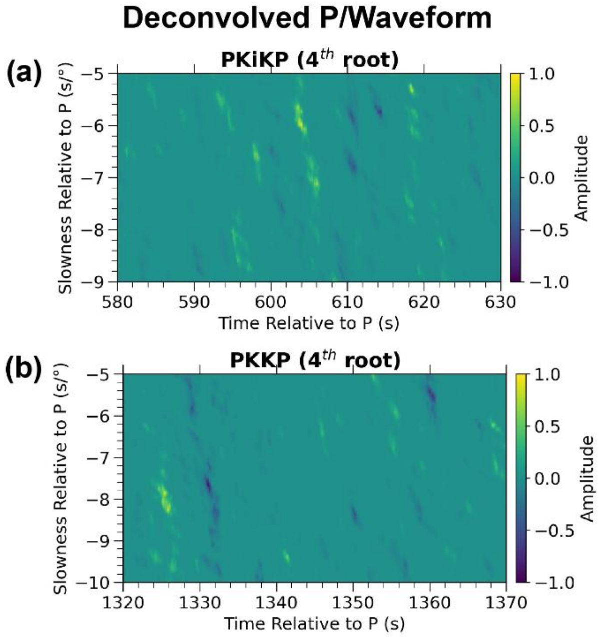

Supplementary Fig. 30. Vespagram analysis of PKKP and PKiKP after deconvolving the P waveform. No prominent energy peaks are presented in both vespagrams.

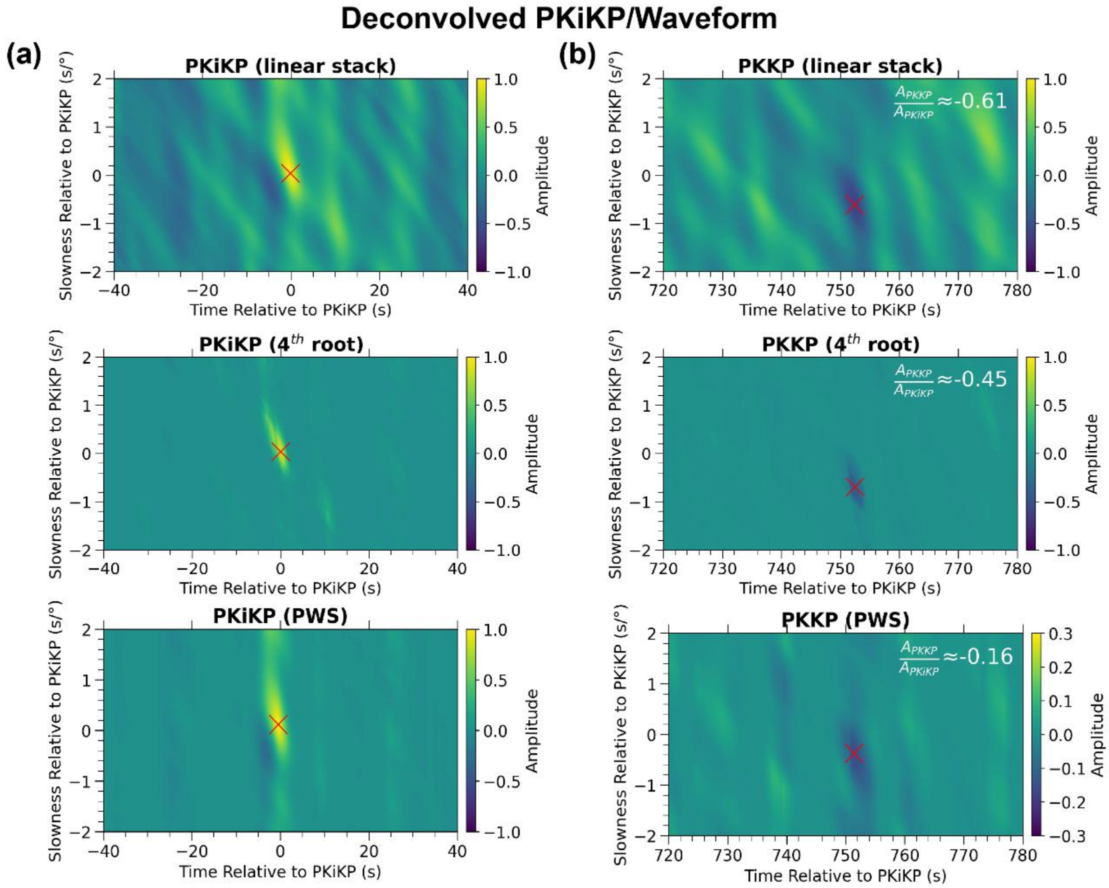

Supplementary Fig. 31. Vespagram analysis of PKiKP and PKKP after deconvolving the PKiKP waveform. (a) shows vespagrams for PKiKP using linear stacking, 4th-root stacking, and PWS methods applied to waveforms after deconvolving PKiKP. Red crosses denote the coherent energy peaks. (b) Same as b, but for PKKP. The calculated amplitude ratio between PKKP and PKiKP is labeled in white text. Note that the amplitude ratio has a negative value, indicating that PKKP and PKiKP have reversed polarities.

3.5 Summary of core phase picking

In this section, we summarize the detection information acquired from the aforementioned methods for core phases P′P′r_ab, P′P′n, PKKP, and PKiKP. This includes the phase arrivals obtained from vespagram analysis and filter bank for individually picked events, along with the frequency band used in polarized analysis. It is important to note that we only consider the arrival times obtained through filter bank analysis as the seismic phase arrivals for individual events, which are further incorporated into the subsequent inversions. The two methods of polarized analysis (DOP and FDPA) are employed to assess the validity of the picked phases. Thus, we do not explicitly measure the arrival times obtained through these polarized analysis methods. Moreover, to better accommodate the uncertainties in the arrival times of the direct P, we use the same methods to pick the P arrivals. Supplementary Tabs. 2-6 list the information in detail.

Supplementary Table 2. Information for polarized analysis (DOP and PCA) and filter bank analysis of direct P on selected high-quality events*

Events	Picked travel time from filter bank analysis t_{ftbk} (s)	Frequency bands for DOP f_{min} (Hz)	f_{max} (Hz)	Frequency bands for PCA f_{min} (Hz)	f_{max} (Hz)
S1015f	3.5±2.2	0.3	0.6	0.3	0.6
S0918a	7.2±2.4	0.5	0.8	0.5	0.8
S0864a	2.8±2.6	0.3	0.6	0.3	0.6
S0235b	2.9±2.0	0.2	0.5	0.2	0.5
S0474a	4.2±2.0	0.3	0.7	0.3	0.7
S0916d	2.4±2.4	0.5	0.8	0.5	0.8
S0802a	2.3±1.9	0.5	0.8	0.5	0.8
S0173a	5.8±2.7	0.3	0.8	0.3	0.8
S0820a	7.9±3.0	0.3	0.6	0.3	0.6
S1048d	2.7±2.2	0.4	0.8	0.4	0.8
S1133c	5.5±3.1	0.5	0.8	0.5	0.8
S1039b	2.9±0.8	0.2	0.5	0.2	0.5
S0290b	4.3±2.1	0.4	0.8	0.4	0.8
S1022a	2.5±1.6	0.2	0.5	0.2	0.5
S0484b	3.0±2.6	0.5	0.8	0.5	0.8
S1197a	3.7±1.6	0.3	0.6	0.3	0.6
S0105a	3.0±1.8	0.3	0.6	0.3	0.6
S0189a	4.2±1.8	0.2	0.4	0.2	0.4
S0784a	4.4±2.6	0.2	0.4	0.2	0.4
S1157a	3.2±2.3	0.15	0.3	0.15	0.3
S1222a	8.7±2.3	0.2	0.4	0.2	0.4
S1012d	4.2±2.5	0.5	0.8	0.5	0.8
S0325a	2.5±2.4	0.4	0.8	0.4	0.8

*The travel time picks are all relative to the P arrivals provided by MQS [28]. t_{ftbk} represents the root-mean-square and standard deviation values of time-domain envelope peaks in filter bank analysis. The incident angles of P are calculated based on the frequency bands listed for DOP and

PCA.

Supplementary Table 3. Summary of travel times of P′P′r_ab relative to P

	P′P′r_ab							
	**Vespa**	**Filter bank**	**FDPA**	**DOP**			**PCA**	
**Events**	t_{vespa} **(s)**	t_{ftbk} **(s)**	t_{FDPA} **(s)**	t_{DOP} **(s)**	f_{min} **(Hz)**	f_{max} **(Hz)**	f_{min} **(Hz)**	f_{max} **(Hz)**
S0173a	1822.4±5.0	1829.1±1.7	1829.1±8.0	1829.1±8.0	0.5	0.8	0.5	0.8
S0235b	1835.1±5.0	1831.5±2.2	1831.5±8.0	1831.5±8.0	0.6	0.9	0.6	0.9
S0802a	1822.4±5.0	1821.4±2.8	1821.4±8.0	1821.4±8.0	0.5	0.7	0.5	0.7
S0864a	1835.1±5.0	1836.6±3.0	1836.6±8.0	1836.6±8.0	0.2	0.4	0.2	0.4
S1015f	1847.7±5.0	1855.2±2.5	1855.2±8.0	1855.2±8.0	0.4	0.8	0.4	0.8
S1022a	1815.2±5.0	1799.7±2.1	1799.7±8.0	1799.7±8.0	0.4	0.6	0.4	0.6
S1048d	1820.6±5.0	1809.8±2.0	1809.8±8.0	1809.8±8.0	0.5	0.8	0.5	0.8
S1222a	1751.9±5.0	1741.7±1.9	1741.7±8.0	1741.7±8.0	0.25	0.4	0.25	0.4
S0918a	1844.1±5.0	1846.6±2.5	1846.6±8.0	1846.6±8.0	0.3	0.5	0.3	0.5

*t_{vespa} denotes the travel time of the phase obtained from vespagram analysis.

Supplementary Table 4. Summary of travel times of P′P′n relative to P

	P′P′n							
	**Vespa**	**Filter bank**	**FDPA**	**DOP**			**PCA**	
**Events**	t_{vespa} **(s)**	t_{ftbk} **(s)**	t_{FDPA} **(s)**	t_{DOP} **(s)**	f_{min} **(Hz)**	f_{max} **(Hz)**	f_{min} **(Hz)**	f_{max} **(Hz)**
S0105a	1994.1±5.0	1990.0±2.5	1990.0±8.0	1990.0±8.0	0.5	0.7	0.5	0.7
S0474a	2007.3±5.0	2007.8±2.6	2007.8±8.0	2007.8±8.0	0.3	0.5	0.3	0.5
S0802a	2003.9±5.0	2003.8±2.6	2003.8±8.0	2003.8±8.0	0.7	0.9	0.7	0.9
S0864a	2008.7±5.0	2007.7±2.2	2007.7±8.0	2007.7±8.0	0.2	0.4	0.2	0.4
S1133c	2003.2±5.0	2005.9±2.1	2005.9±8.0	2005.9±8.0	0.5	0.7	0.5	0.7

Supplementary Table 5. Summary of travel times of PKKP relative to P

	PKKP							
	**Vespa**	**Filter bank**	**FDPA**	**DOP**			**PCA**	
**Events**	t_{vespa} **(s)**	t_{ftbk} **(s)**	t_{FDPA} **(s)**	t_{DOP} **(s)**	f_{min} **(Hz)**	f_{max} **(Hz)**	f_{min} **(Hz)**	f_{max} **(Hz)**
S0235b	1346.4±5.0	1347.8±1.9	1347.8±8.0	1347.8±8.0	0.4	0.6	0.4	0.6
S0474a	1343.7±5.0	1343.7±1.3	1343.7±8.0	1343.7±8.0	0.5	0.7	0.5	0.7
S0484b	1323.4±5.0	1326.5±1.8	1326.5±8.0	1326.5±8.0	0.15	0.3	0.15	0.3
S0802a	1336.9±5.0	1330.7±2.6	1330.7±8.0	1330.7±8.0	0.5	0.8	0.5	0.8
S0918a	1353.2±5.0	1347.6±1.4	1347.6±8.0	1347.6±8.0	0.5	0.8	0.5	0.8
S1012d	1274.7±5.0	1274.9±2.5	1274.9±8.0	1274.9±8.0	0.2	0.4	0.2	0.4
S1048d	1335.6±5.0	1320.4±2.4	1320.4±8.0	1320.4±8.0	0.25	0.4	0.25	0.4

Supplementary Table 6. Summary of travel times of PKiKP relative to P

	PKiKP							
	**Vespagram**	**Filter bank**	**FDPA**	**DOP**			**PCA**	
**Events**	t_{vespa}* **(s)**	t_{ftbk} **(s)**	t_{FDPA} **(s)**	t_{DOP} **(s)**	f_{min} **(Hz)**	f_{max} **(Hz)**	f_{min} **(Hz)**	f_{max} **(Hz)**
S0235b	604.6±5.0	603.8±1.6	603.8±8.0	603.8±8.0	0.7	1	0.5	0.8
S0802a	596.0±5.0	596.4±1.8	596.4±8.0	596.4±8.0	0.5	0.7	0.5	0.7
S0864a	604.6±5.0	602.4±2.2	602.4±8.0	602.4±8.0	0.4	0.7	0.4	0.7
S1015f	613.2±5.0	608.4±2.7	608.4±8.0	608.4±8.0	0.5	0.7	0.5	0.7
S1039b	593.6±5.0	606.4±3.0	606.4±8.0	606.4±8.0	0.5	0.8	0.5	0.8
S1197a	582.5±5.0	586.5±1.2	586.5±8.0	586.5±8.0	0.2	0.5	0.2	0.5
S1012d	539.5±5.0	548.0±1.5	548.0±8.0	548.0±8.0	0.4	0.6	0.4	0.6

4 Inversions

4.1 Seismic model parameterization

To further justify our decision to focus solely on inverting core velocity structure, we examine the effect of different mantle structure on the differential travel time between PKKP and PKiKP ($\Delta T_{PKKP\text{-}PKiKP}$). We first fix the core velocity and test a range of mantle models (Supplementary Fig. 32a-b), including both available mantle velocity models (Supplementary Fig. 32a) and some more extreme 1D velocity models (Supplementary Fig. 32b). As shown in Supplementary Fig. 32d, $\Delta T_{PKKP\text{-}PKiKP}$ across different mantle models vary little, supporting the feasibility of exclusively inverting core velocity structure by having both measurements of $\Delta T_{PKKP\text{-}P}$ and $\Delta T_{PKiKP\text{-}P}$. Additionally, we explore the effect of varying outer-core (OC) velocity by perturbing the P-velocity by -10%, -5%, +5% and +10% (Supplementary Fig. 32c). These changes can produce significant variations in $\Delta T_{PKKP\text{-}PKiKP}$ (Supplementary Fig. 32d), indicating a strong trade-off between the P-velocity perturbation (δV_P) in the OC and the inner-core (IC) size (Supplementary Fig. 32e). However, by incorporating additional constraints from $\Delta T_{P'P'r\_ab\text{-}P}$ and $\Delta T_{P'P'n\text{-}P}$, we are able to reduce this trade off, although large uncertainty in the IC size remains with our limited observations. Moreover, we refrain from amalgamating travel times obtained in previous studies [1-4, 36, 37] and our new measurements to invert the whole Martian velocity structure, since the mantle near the antipode sampled by the P′P′ could potentially be heterogeneous. Nevertheless, to assess how different mantle models can affect the inversions, we select seven mantle models, including the mean, lower, and upper bound V_P models from the AK_subset models [1], two V_P models inverted by incorporating SKS travel times [2], and two models with MSL [3, 4] to perform inversion separately.

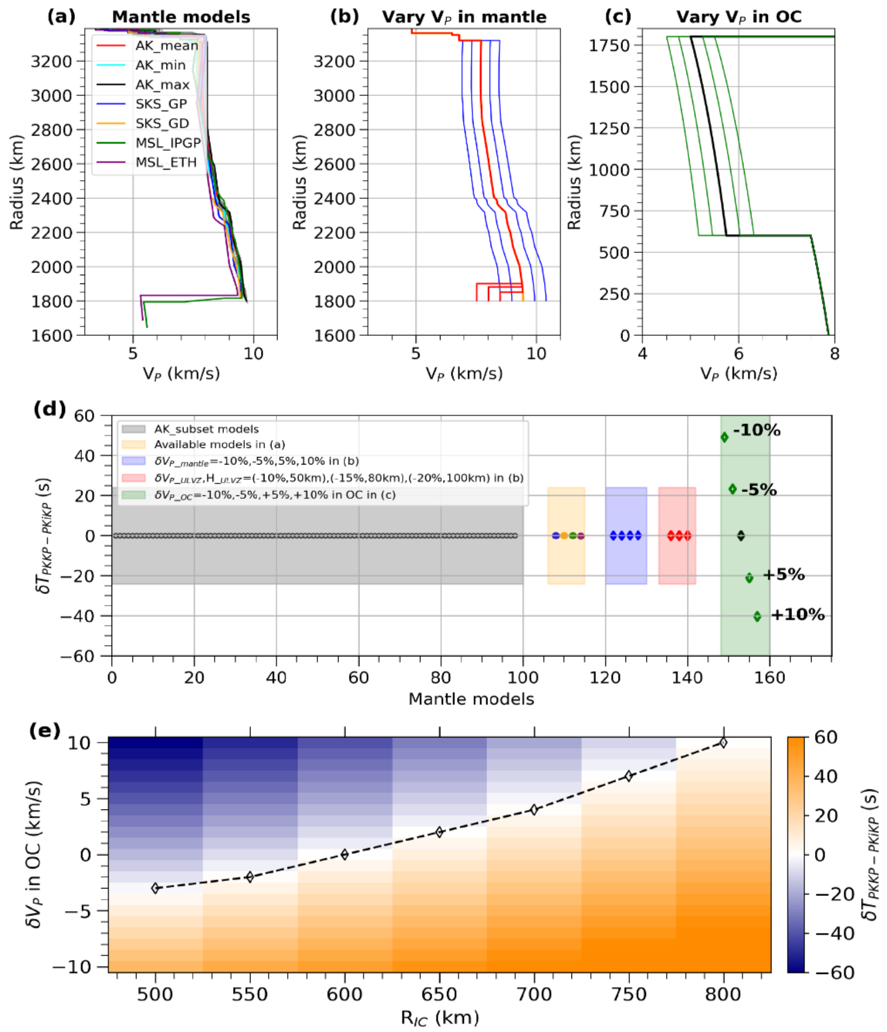

Supplementary Fig. 32. Effect of the mantle and core structures on $\Delta T_{PKKP\text{-}PKiKP}$. (a) Various mantle P-wave velocity models. The light gray lines show all 99 seismic models in the AK_subset models [1]. The mean, lower, and upper bound of the AK_subset models correspond to red, cyan, and black solid lines, respectively. The blue and orange lines represent the best "geophysical" (SKS_GP) and "geodynamical" (SKS_GD) inversion model incorporating SKS travel times [2]. The green and purple lines represent the inverted V_P models containing MSL by Samuel et al. (2023) [4] and Khan et al. (2023) [3], respectively. (b) Martian mantle structures with P-velocity perturbations and low-velocity-zone (LVZ) at the core-mantle boundary (CMB). The blue lines, from left to right, represent uniform mantle P-velocity perturbations of -10%, -5%, +5%, and +10%, respectively. The red lines, from left to right, represent LVZs with a δV_P of -20% and a height of 100 km, -15% and a height of 80 km, and -10% and a height of 50 km, respectively. (c) Martian core structure with P-velocity perturbations. The black line represents the assumed the reference Martian core structure, while the green lines, from left to right, represent outer core P-velocity perturbations of -10%, -5%, +5% and +10%, respectively. (d) $\Delta T_{PKKP\text{-}PKiKP}$ for different mantle and core velocity models. All $\Delta T_{PKKP\text{-}PKiKP}$ values are corrected by subtracting the mean differential time computed for the AK_subset models. (e) Trade-off between the outer core P-velocity perturbations and inner core size. The black symbols denote the points with zero deviation from the mean of $\Delta T_{PKKP\text{-}PKiKP}$ calculated using AK_subset models, which suggests that a 5% change in the outer core velocity could cause a variation of R_{IC} by as much as 100 km.

As shown in Extended Data Fig. 4 and Supplementary Table 7, we define five model parameters characterizing the P-wave velocity (V_P) structure of the Martian core: the outer-core (OC) radius (R_{OC}), the V_P at the CMB (V_{P_CMB}), the V_P of the outer-core

side at the ICB ($V_{P\_OC\_ICB}$), the IC radius (R_{IC}), and the V_P jump at ICB (δV_{P_ICB}). Here, the model spaces of the OC are based on the results of Irving et al. (2023) [2]. We further assume that the mean velocity gradients in the OC and IC are the same. The model parameters in the case of the model with a MSL are clarified in **Methods**.

Supplementary Table 7. Inverted Martian core parameters and their corresponding prior bounds

Description	Parameter	Value/range
**Outer-core**		
Outer-core radius	R_{OC}	1700 - 1900 km
P-wave velocity at the CMB	V_{P_CMB}	4.6 - 5.5 km/s
P-wave velocity of the outer-core side at the ICB	$V_{P\_OC\_ICB}$	5.0 - 6.5 km/s
**Inner-core**		
Inner-core radius	R_{IC}	0.5 - 750 km
P-wave velocity jump at ICB	δV_{P_ICB}	1 % - 60 %

4.2 Inversion results

Our inversion results, using different combinations of mantle models and observed datasets, are plotted in Supplementary Figs. 33-36. Here, we focus on the inversion results using the M_vesp dataset, while the results from the M_pick dataset are provided in **Supplementary Information B**. The inverted seismic velocity models and their corresponding fits to the observed travel times of different core phases are shown in Supplementary Figs. 38-40.

The inversion results show a consistent IC radius of 530–700 km, regardless of the choices of the data and mantle models (Supplementary Figs. 33-36). Notably, the IC radius emerges as a robust parameter, while other parameters exhibit strong trade-offs among them (Supplementary Fig. 37). It is worth mentioning that an inversion incorporating the slowness of PKiKP (Supplementary Fig. 34) could help to reduce the uncertainty in the IC radius.

In contrast, the OC radius is not well defined and also notably sensitive to mantle structures. For instance, using the AK_max model (Supplementary Fig. 35a), an upper bound mantle V_P model in the AK_subset models, produces an OC radius of 1,816 ± 67 km, significantly larger than the radius of 1,787 ± 61 km inverted for the AK_min model, featuring a lower bound model. Seismic observations of core-reflected seismic waves (ScS) report an OC radius of 1,830 ± 40 km [1]. Based on identified PcP from single-station correlation [50, 51] and ScS* from intersource correlations [52], the core radius is constrained to 1,560 ± 40 km and 1,812 ± 20 km, respectively. Irving et al. (2023) concludes a median OC radius of 1,780 to 1,810 km from their observed SKS data [2]. Similarly, the MSL_ETH model, which has the slowest mantle velocity of all models (Supplementary Fig. 32a) and a 142 km-thick MSL over the core, yields the smallest OC radius of 1,626±54 km (Supplementary Fig. 36a). This agrees with previously inverted OC radius of 1,650±20 km [4] and 1,675±30 km [3] assuming the presence of an MSL. Our inversion results also highlight challenges in resolving V_{P_CMB}. In contrast, the V_P of the outer-core side at the ICB ($V_{P\_OC\_ICB}$) is better resolved with a value of 5.7± 0.2 km, regardless of the mantle models chosen. In summary, while

significant uncertainties exist, our inverted OC properties broadly consistent with previous studies.

The velocity jump at the ICB provides important constraint on the geophysical properties of IC. However, as shown in Supplementary Figs. 33-36, this parameter is not well resolved in the inversions, which may arise from the fact that only the travel times of PKKP provide constraints on the IC velocity and there exist trade-offs between the OC parameters and IC velocity. To improve the resolution of δV_{P_ICB}, additional inversions are conducted by narrowing the ranges of OC properties, as obtained in Irving et al. (2023) [2]. Supplementary Table 8 lists the refined prior information of model parameters. Supplementary Fig. 41 displays the inversion result, illustrating that the uncertainty of δV_{P_ICB} remains large. Furthermore, considering that the best "geodynamical" inverted model provided in Irving et al. (2023) [2] effectively predict our P′P′ measurements (Supplementary Fig. 42), we set the OC parameters identical to those of the SKS_GD model and only invert R_{IC} and δV_{P_ICB}. Although this refined inversion reduces the uncertainty of the δV_{P_ICB}, with δV_{P_ICB} of $32 \pm 8\%$, equivalent to an IC V_P of 7.3-8.3 km/s (Supplementary Fig. 43), the effect is limited. Nonetheless, this updated inversion also produces a robust R_{IC} of 612 ± 50 km. We further select the velocity model with the minimal misfit from those models falling within the ranges of V_{P_CMB} (4.9-5.0 km/s) and R_{OC} (1,780-1,810 km) as indicated by the light blue band in Supplementary Figs. 33-36, consistent with Irving et al. (2023) [2]. These optimal models are collectively illustrated in Extended Data Fig. 5. For our subsequent analysis, we focus exclusively on the inverted model based on the SKS_GD model (BS_SKS_GD_IC model in Extended Data Fig. 5).

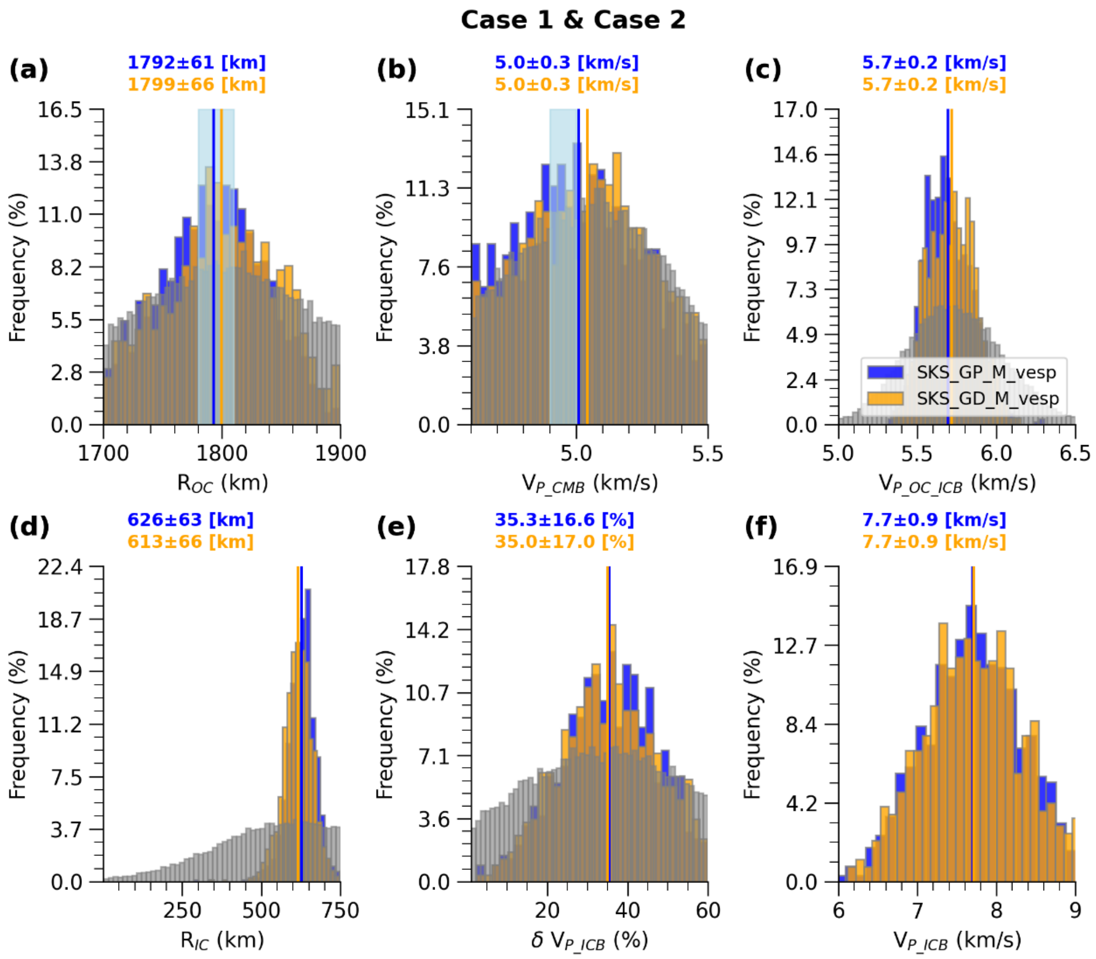

Supplementary Fig. 33. Marginal distributions of inverted core parameters using the M_vesp method, for case 1 and case 2. (a) Outer-core radius (R_{OC}), (b) P velocity at the CMB (V_{P_CMB}), P velocity of the outer-core side at the ICB ($V_{P\_OC\_ICB}$), (d) inner-core radius (R_{IC}), (e) P velocity jump at the ICB (δV_{P_ICB}), and (f) P velocity of the IC side at the ICB (V_{P_ICB}). Note that V_{P_ICB} is not an inverted parameter and is directly calculated from $V_{P\_OC\_ICB}$ and δV_{P_ICB}. The blue and orange histograms correspond to the posteriori distributions of two different mantle velocity models, namely SKS_GP and SKS_GD, with their mean values depicted by solid lines of corresponding colors. Mean values and 85% confidence intervals are indicated with colored text at the top. The gray histograms represent the priori distribution using the SKS_GD mantle velocity model. The light blue shaded regions outline the determined ranges of V_{P_CMB} and R_{OC} from Irving et al. (2023) [2].

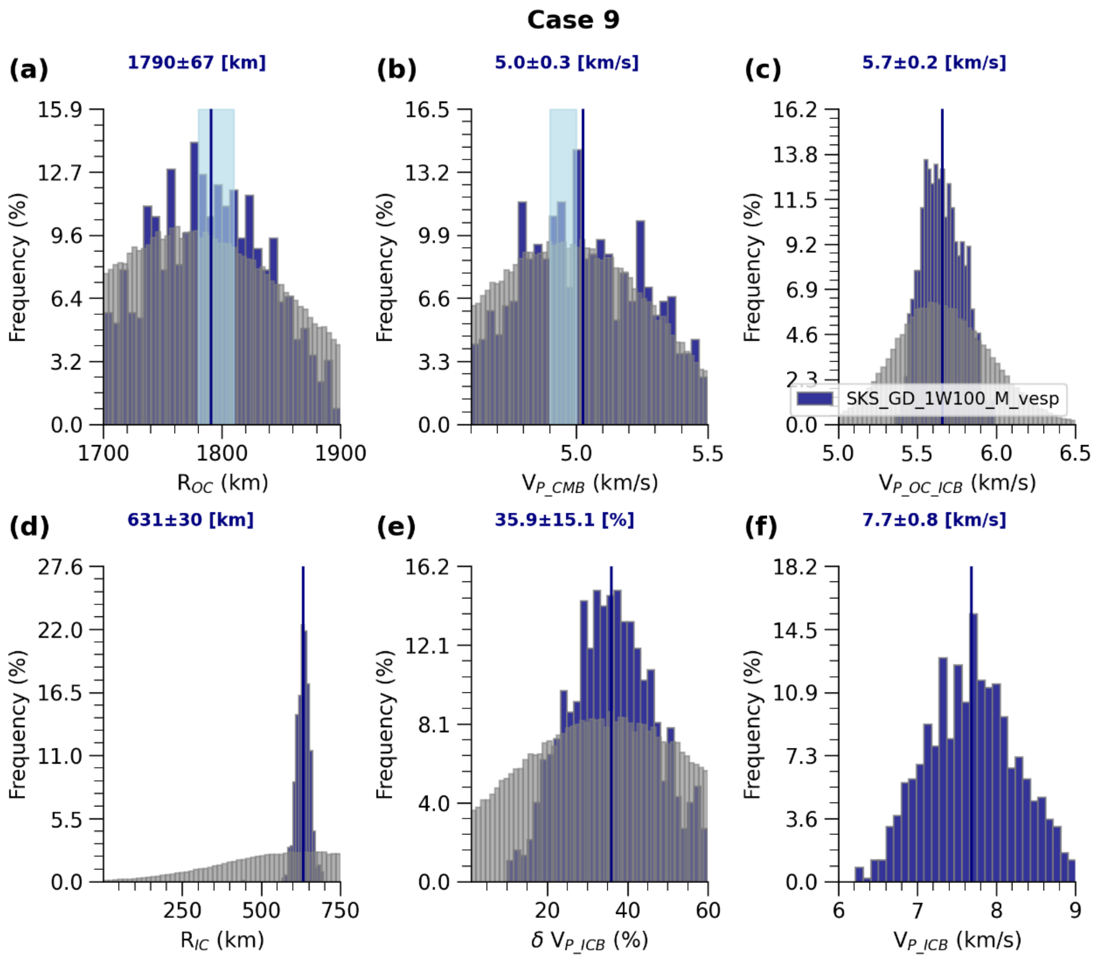

Supplementary Fig. 34. Marginal distributions of inverted core parameters using the M_vesp method, for case 9. Same as Supplementary Fig. 33, but incorporating the slowness of PKiKP with a weight of 100 into the inversion.

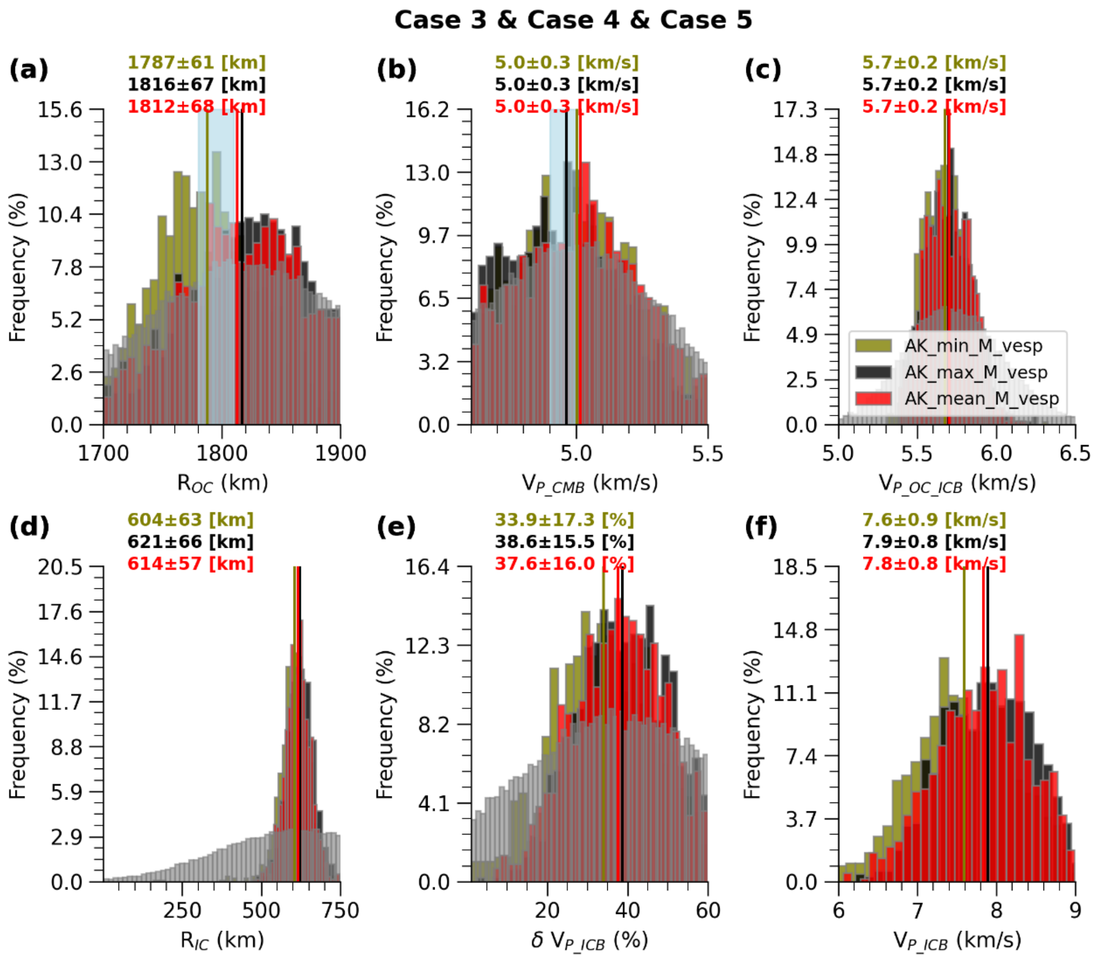

Supplementary Fig. 35. Marginal distributions of inverted core parameters based on AK_min (cyan; case 3), AK_max (black; case 5), and AK_mean (red; case 4) models. The gray histograms represent the priori distribution using the AK_mean model.

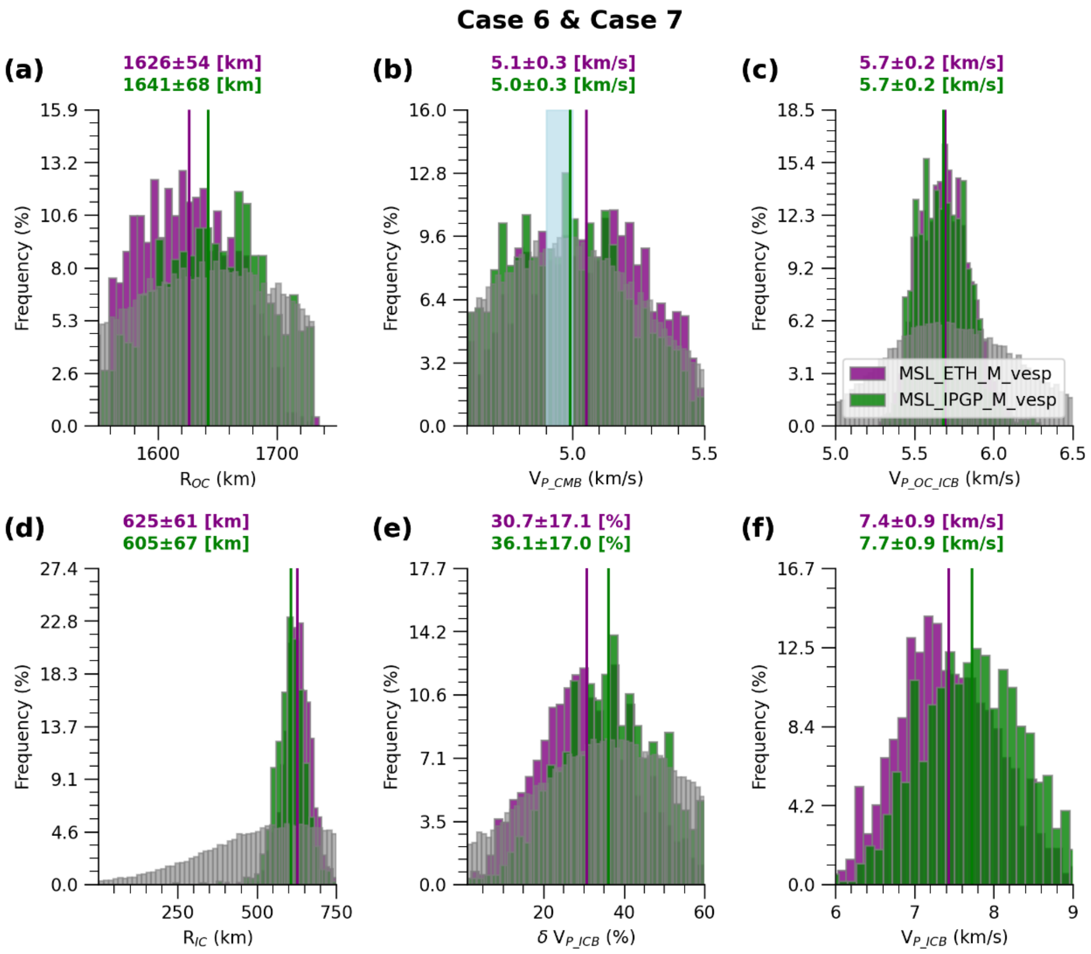

Supplementary Fig. 36. Marginal distributions of inverted core parameters based on MSL_IPGP (green; case 7) and MSL_ETH (purple; case 6) model. The gray histograms represent the priori distribution using the MSL_ETH model. Note that the outer-core radius (R_{OC}) here is smaller than those inverted from models without MSL (Supplementary Figs. 33-35), based on the assumption that the inverted PKKP is actually $PKKP_{MSL}$ (Supplementary Figs. 12-13).

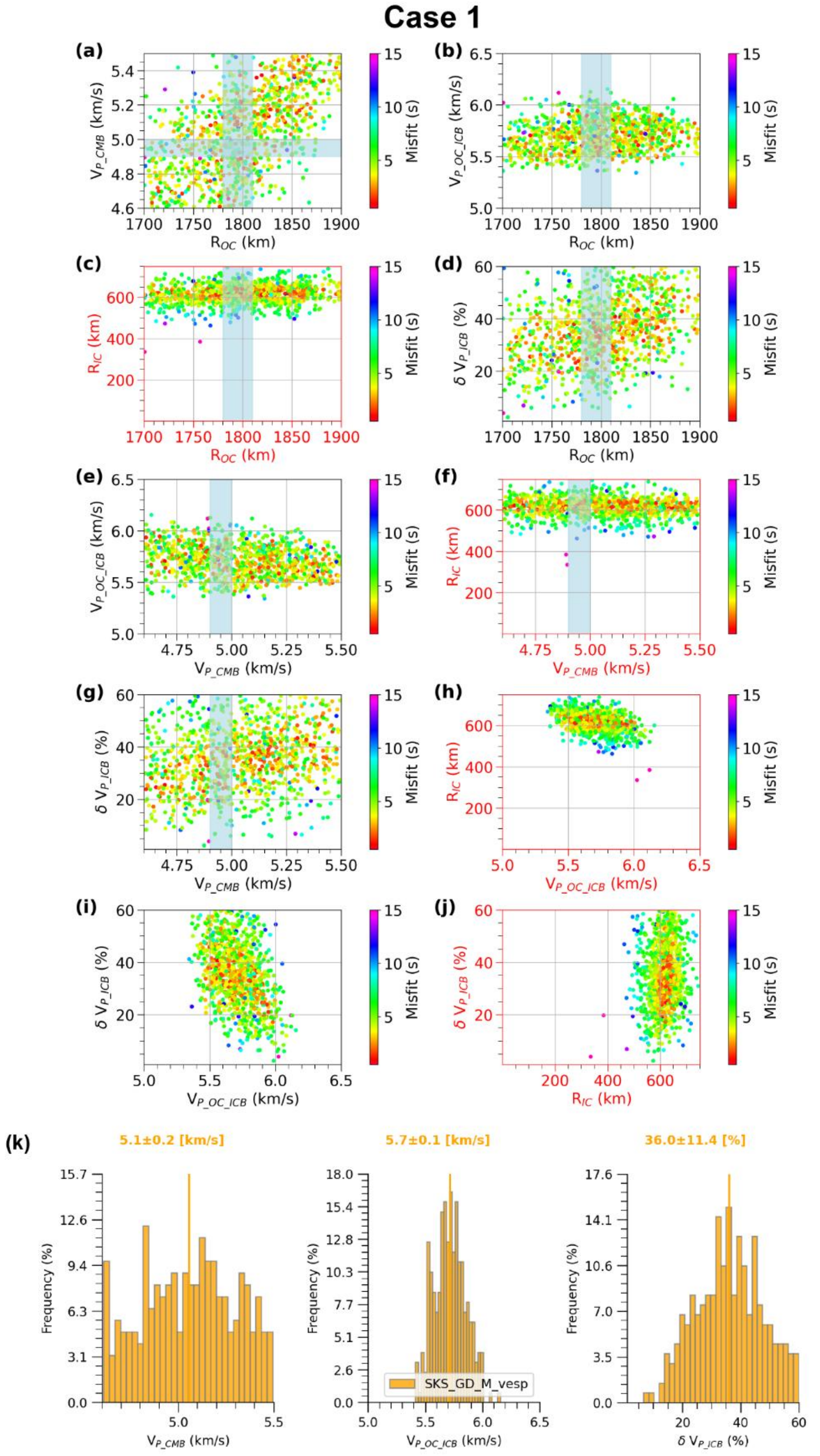

Supplementary Fig. 37. Trade-offs among the five inverted core parameters based on the SKS_GD model (case 1). Light blue shaded regions outline the determined ranges of V_{P_CMB} and R_{OC} from Irving et al. (2023) [2]. The four subfigures (c, f, h, and i) with red axes highlight the trade-offs between the inner-core radius (R_{IC}) and the other four parameters. (k) Marginal distributions of inverted core velocities sampled within the 25% confidence interval of the inferred inner core (IC) radius (R_{IC}=613 ± 18 km) for case 1. From left to right: V_{P_CMB}, $V_{P\_OC\_ICB}$, and δV_{P_ICB}. Mean values and 1σ are indicated with colored text at the top.

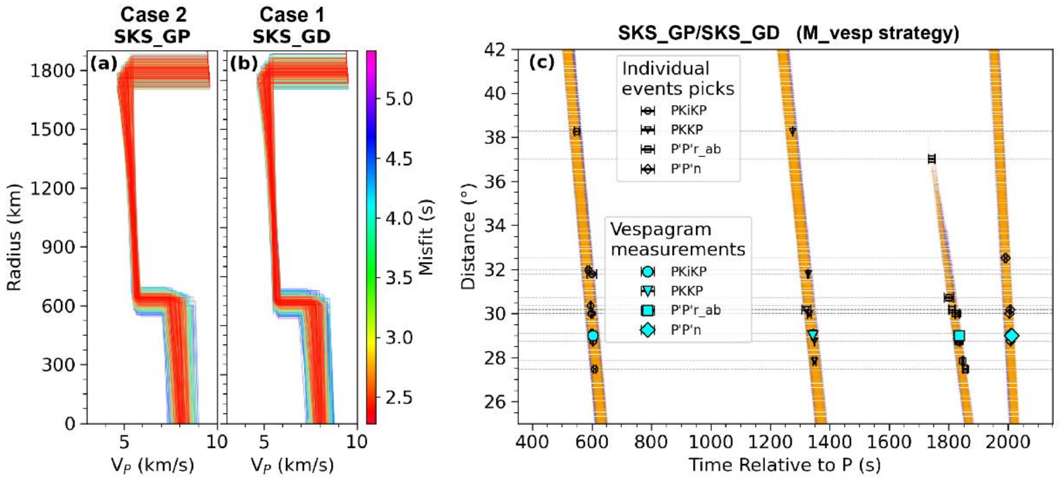

Supplementary Fig. 38. P velocity profiles of the Martian core based on the inverted parameters for two mantle models of (a) SKS_GP and (b) SKS_GD. The plots display results using the M_vesp strategy, in which only four travel times (cyan solid symbols in (c)) from the vespagram measurements are used for the inversion here. The colored lines in (a)-(b) represent differential travel time misfits between the data and predictions from the inverted models. (c) Predicted differential travel times for all models in (a)-(b). Blue and orange lines denote the predictions from the inverted core models based on the SKS_GP and SKS_GD models, respectively. The open and cyan solid symbols denote the individual events picks and vespagram measurements, respectively.

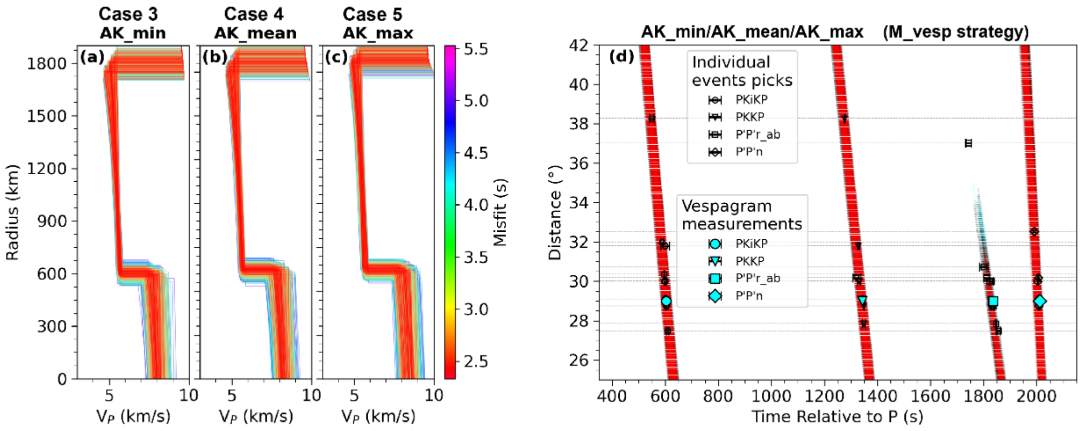

Supplementary Fig. 39. P velocity profiles of the Martian core based on the inverted parameters for three mantle models from the AK_subset models. (a) AK_min, (b) AK_mean, and (c) AK_max model. In (d), cyan, red, and black lines denote the predictions from the inverted core models based on the AK_min, AK_mean, and AK_max models, respectively.

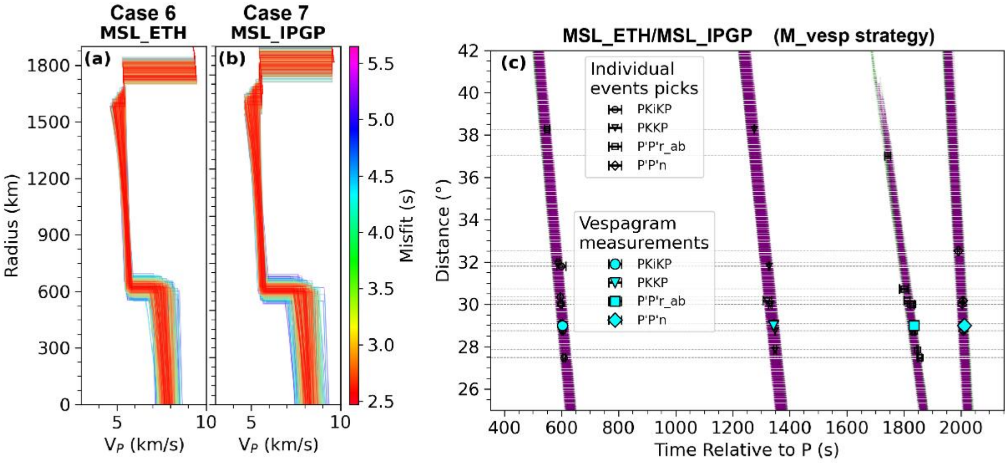

Supplementary Fig. 40. P velocity profiles of the Martian core based on the inverted parameters for two mantle models of (a) MSL_IPGP and (b) MSL_ETH. In (c), the green and purple lines denote predictions from the inverted core models based on MSL_IPGP and MSL_ETH models, respectively. Note that PKKP in (c) is actually $PKKP_{MSL}$ (Supplementary Figs. 12-13).

Supplementary Table 8. Refined prior bounds of the core parameters*

Description	Parameter	Value/range
**Outer-core**		
Outer-core radius	R_{OC}	1780 - 1810 km
P-wave velocity at the CMB	V_{P_CMB}	4.9 - 5.0 km/s
P-wave velocity of the outer-core side at the ICB	$V_{P\_OC\_ICB}$	5.0 - 6.5 km/s
**Inner-core**		
Inner-core radius	R_{IC}	0.5 - 750 km
P-wave velocity jump at the ICB	δV_{P_ICB}	1 % - 60 %

*The ranges of the outer core parameters are based on the results in Irving et al. (2023) [2].

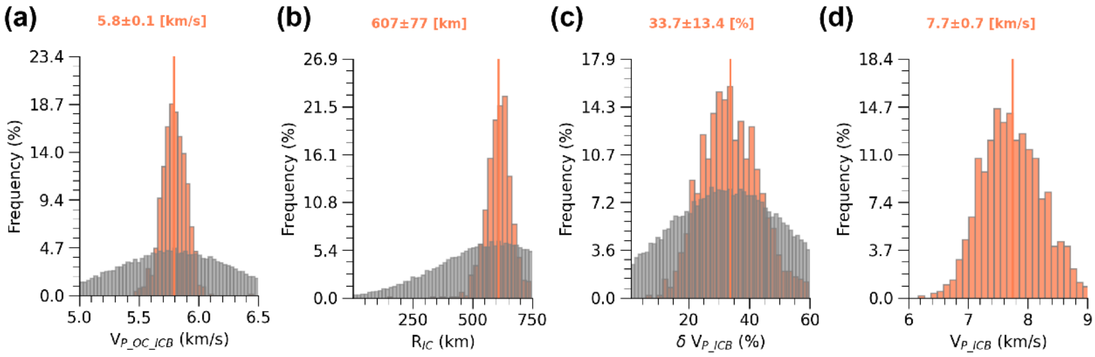

Supplementary Fig. 41. Marginal distributions of the inverted core parameters with narrower OC model spaces as in Supplementary Table 8.

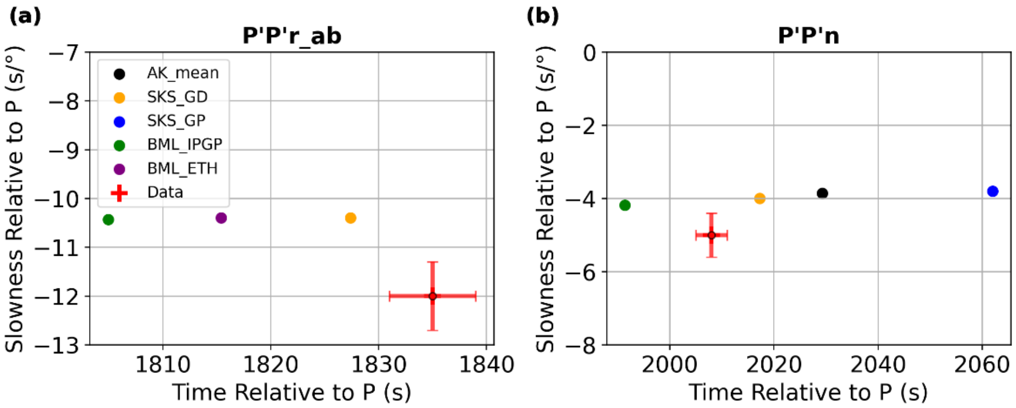

Supplementary Fig. 42. Predicted travel time and slowness of the P′P′ phase at the epicentral distance of 29° using original velocity models without the IC. (a) and (b) are for P′P′r_ab and P′P′n, respectively. Red circles with errors represent the observations obtained from the vespagram analysis.

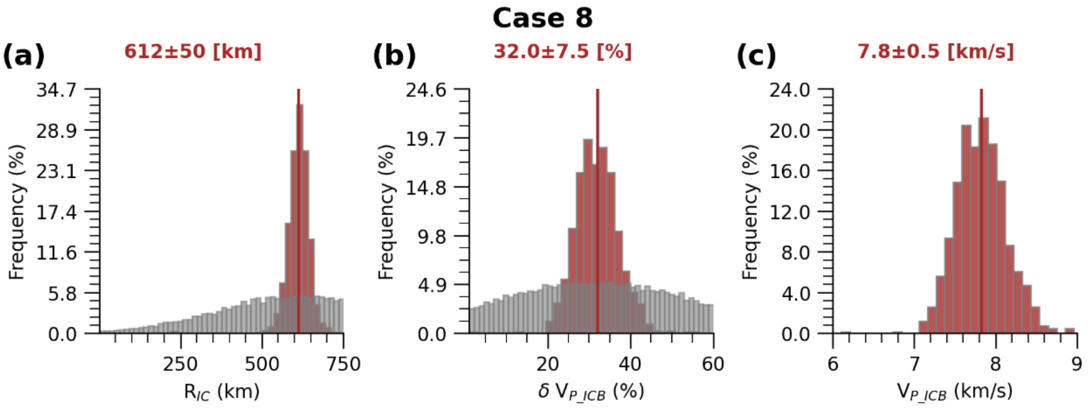

Supplementary Fig. 43. Marginal distributions of the IC parameters by fixing the OC parameters in the inversion. The OC parameters are identical to those of the model SKS_GD. (a)-(c) show Inner-core radius (R_{IC}), P velocity jump at ICB (δV_{P_ICB}), and P velocity of the IC side at the ICB (V_{P_ICB}), respectively.

5 Further supports for the core with an IC

5.1 PKiKP for large distance events

In the vespagram analysis, we focus on high-quality marsquakes in the distance range of 27°–40° for the detection of core phases. Additional quality-A or -B events with larger epicentral distances in the MQS catalog (Extended Data Table 1), including S0183a, S0185a, S1102a, S1153a, S1415a, S0976a and two impact events, S1000a and S1094b, serve as important events to validate our inverted core model. In particular, we aim to examine whether PKiKP can be identified for these events in the presence of an IC.

Supplementary Fig. 44 exhibits the polarization filtered waveforms and envelopes on the vertical component for three events at large distances, where PKiKP phases are clearly illustrated. These observations are further supported by their FDPA results, as shown in Supplementary Fig. 45. Although other potential core phases arrivals are also

marked on the waveforms, we exercise caution in placing high expectations on them due to large uncertainties arising from possible structure complexity and possible contaminations from other phases.

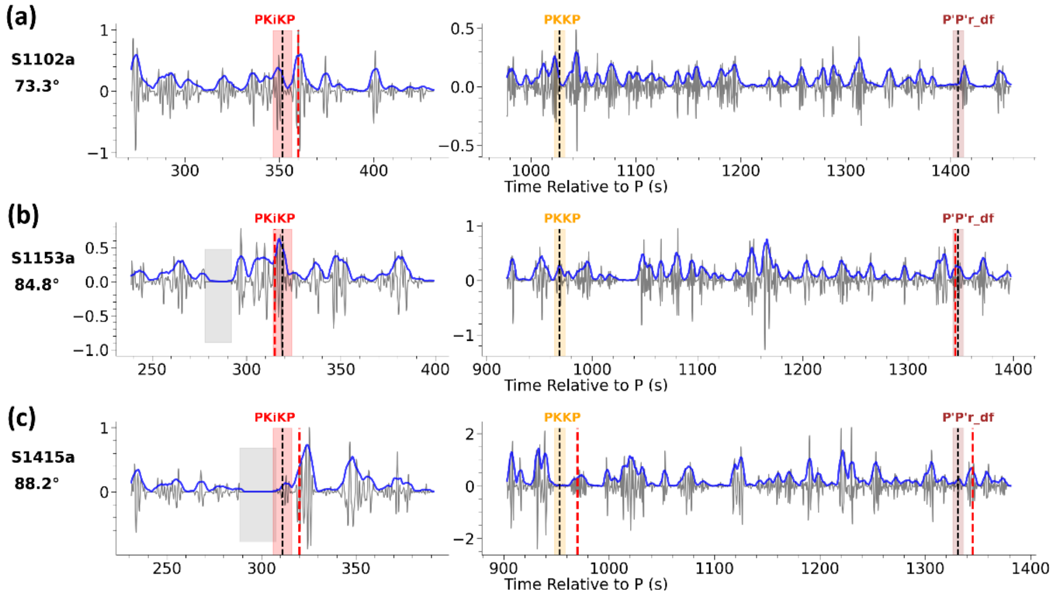

Supplementary Fig. 44. Polarization filtered waveforms and envelopes on the vertical component for three events at large distances. Panels (a), (b), (c) correspond to events S1102a, S1153a, and S1415a, respectively. Their epicentral distances, provided by MQS [28], are displayed on the left of each panel. The black lines denote the predicted arrival times of annotated phases using the inverted model of BS_SKS_GD_IC (case 8 in Extended Data Table 2), with colored shaded regions indicating a time interval of ± 5 s around phase arrivals. Red lines mark the arrival times of phases computed by frequency-dependent polarization analysis (FDPA). Gray shaded regions indicate the presence of known instrument glitches. To facilitate amplitude comparison, both segments of polarization filtered waveforms are normalized by the maximum amplitude in the first one including the PKiKP phase.

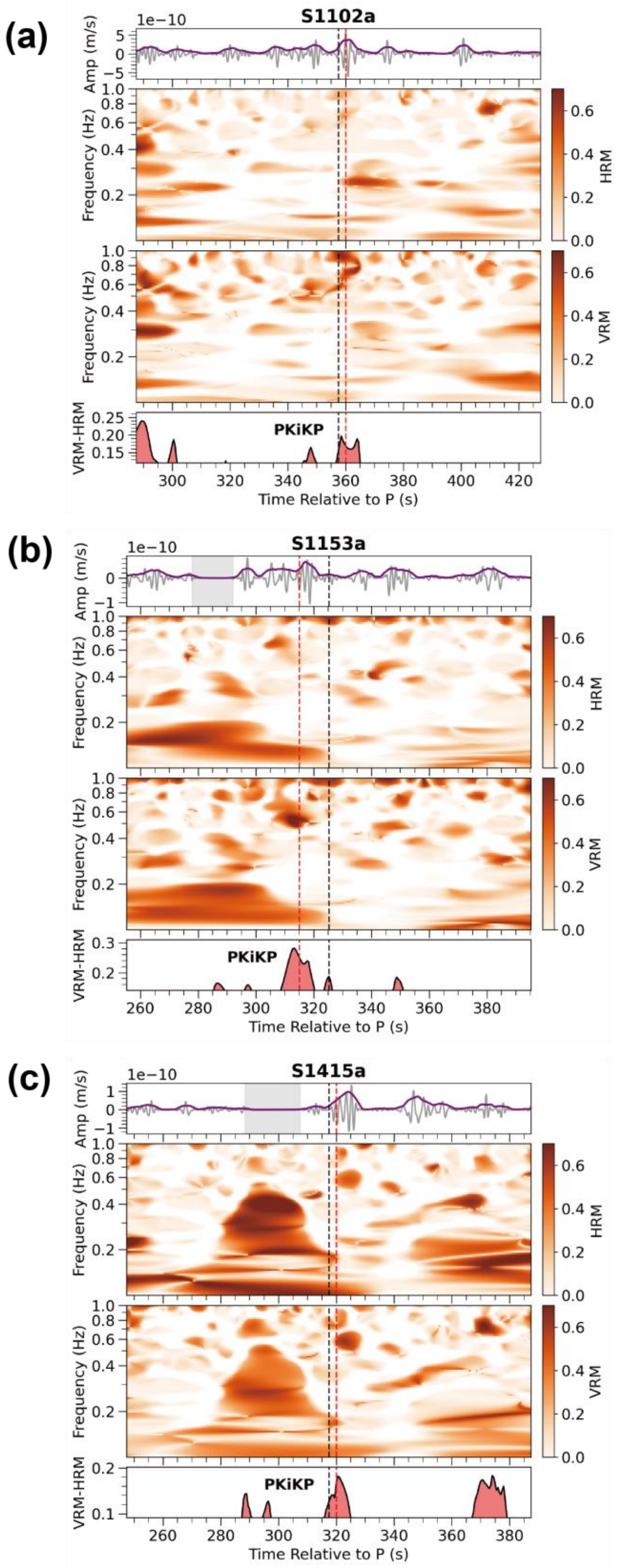

Supplementary Fig. 45. Frequency dependent polarization analysis (FDPA) for PKiKP at large distance. Panels (a), (b), (c) correspond to events S1102a, S1153a, and S1415a, respectively. Each subfigure presents, from top to bottom: Polarized filter waveform and envelope on vertical component, Horizontal component of FDPA analysis (0.1–1.0 Hz), Vertical component of FDPA analysis, and Averaged excess linearly polarized energy across 0.5–0.8 Hz. The black and red dashed lines denote the predicted travel time using the inverted model of BS_SKS_GD_IC and the measured travel time by FDPA, respectively.

5.2 P′P′r_df phase

In the presence of an IC with a radius of ~600 km, a distinct branch of P′P′, i.e., PKIKPPKIKP or P′P′r_df, travels through the IC (Supplementary Fig. 46a), in addition to rays propagating through the OC (P′P′r_ab and P′P′n) within the distance range of 27°–40°. It is worth noting that the P′P′r_bc is weak for this new model with IC, which offers a neat explanation for the observed P′P′r_bc signals with low energy in the vespagrams, as discussed in **Section 3.2.2.** Furthermore, P′P′r_df arrives much earlier than P′P′r_ab due to the high velocity IC (Supplementary Fig. 46b). If visible energy corresponding to the time and slowness for P′P′r_df can be identified, it can provide crucial evidence for the existence of an IC.

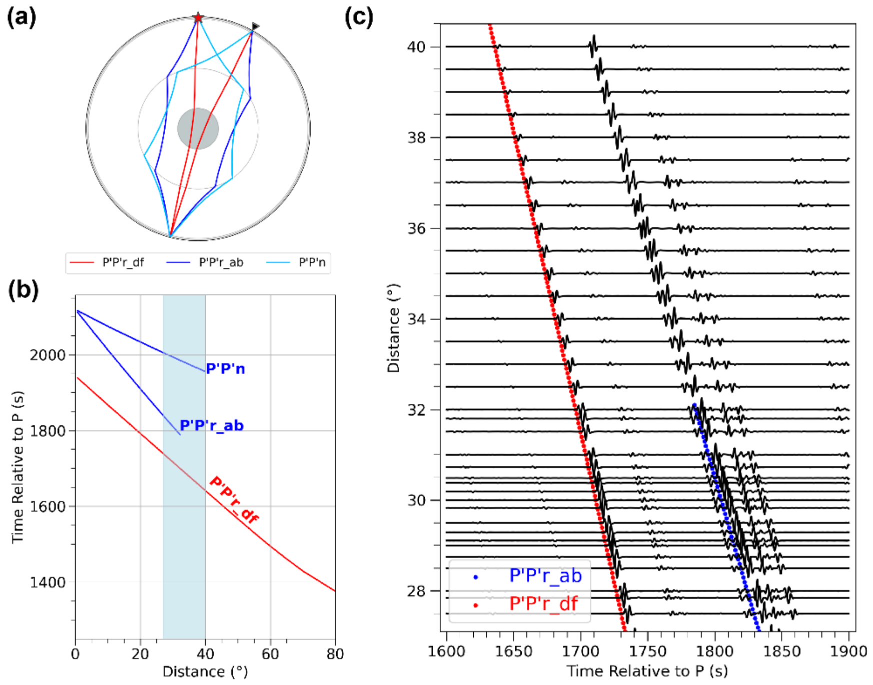

Supplementary Fig. 46. Synthetic waveforms of P′P′r_df. (a) Ray path diagram of P′P′r_df at a distance of 29°. (b) Travel time curves of P′P′ phases relative to P. The light blue shaded region denotes the targeted distance range of 27°–40°. (c) Synthetic seismograms of P′P′r_df and P′P′r_ab in the distance range of 27°–40°, and their travel times relative to P are marked with red and blue lines, respectively.

Although the synthetics suggest that P′P′r_df has a weak amplitude compared to P′P′r_ab (Supplementary Fig. 46c), it can be readily identified in the vespagram of the data, showing a distinct coherent energy peak at the expected arrival time (Extended Data Fig. 6d-f). Moreover, bootstrap resampling test consistently shows energy peaks at ~1,715 s with a slowness of -7.0 s/° relative to the P. This result closely matches the model predictions (Extended Data Fig. 6f), further supporting the identification of P′P′r_df.

5.3 PKIIKP phase

In the presence of an IC, the PKIIKP phase, a reflection from the inner side of the ICB, is expected (Supplementary Fig. 47a). Interestingly, within the distance range of 27°–40°, the PcSScP phase overlaps with the time window of the PKIIKP phase (Supplementary Fig. 47b). Both phases are clearly visible in the synthetics (Supplementary Fig. 47c) and vespagram for the data (Extended Data Fig. 6g-i). However, as shown in Supplementary Fig. 47c, the amplitude of the PcSScP phase is much larger than that of the PKIIKP phase, which is also evident in the vespagram for the data (Extended Data Fig. 6g-h). This makes a robust identification of PKIIKP challenging. Additionally, the low slowness resolution observed may be due to the overlap of the two phases, complicating their separation though array analysis.

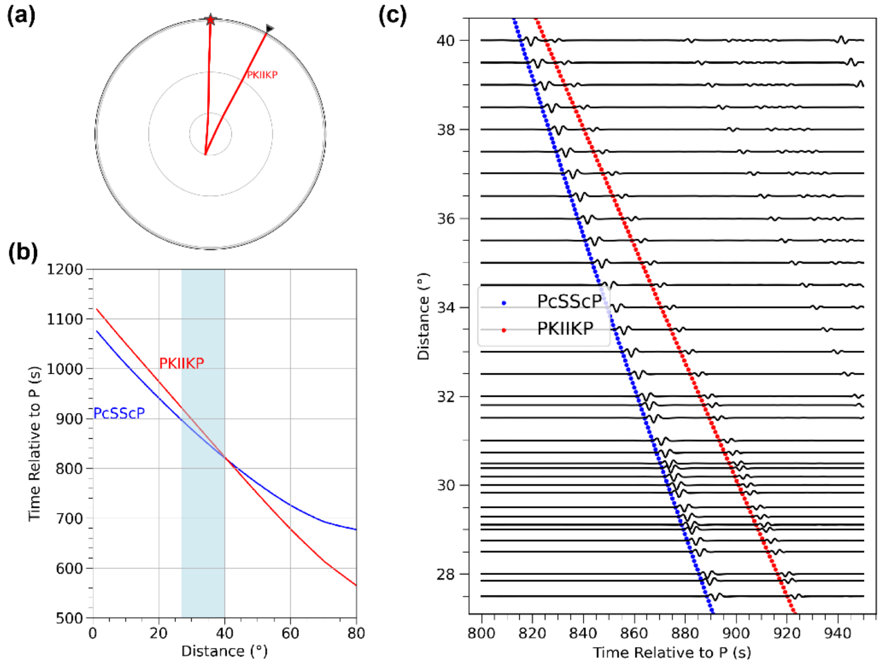

Supplementary Fig. 47. Synthetic waveforms of PKIIKP. (a) Ray path diagram of PKIIKP at a distance of 29°. (b) Travel time curves of PKIIKP and PcSScP relative to P. The light blue shaded region denotes the targeted distance range of 27°–40°. (c) Synthetic seismograms of PcSScP and PKIIKP in the distance range of 27°–40° and their travel times relative to P are marked with blue and red lines, respectively.

6 Density of the IC

6.1 The amplitude ratio between PKiKP and PKKP

The amplitude of PKiKP is largely controlled by the impedance contrast at the ICB. Therefore, we can use PKKP as a reference phase and examine the amplitude ratio between PKiKP and PKKP (**Section 3.4**) to estimate the density jump across the ICB. Furthermore, event S0235b has both phases visible in the data, enabling us to conduct a series of synthetic tests to examine the optimal density jump to match the observation.

We then generate synthetic waveforms with the inverted velocity model BS_SKS_GD_IC, while systematically varying the density jump at the ICB from 0% to 50%. Here, an explosion source at a depth of 33 km is assumed. Extended Data Fig.

7 shows the waveform comparisons. Although the detailed waveforms of PKiKP are not fully modelled, a density jump at the ICB of 7 $\pm$ 5% is preferred to explain the observed amplitude ratio. However, it is important to acknowledge that the estimation presented here may be subject to significant uncertainties, due to the unknown source mechanism, heterogeneities in the mantle, attenuation effects, and less defined CMB structures.

6.2 Geodesy constraints

The geodesy measurements, such as its mean planetary density, moment of inertia (MOI), and solar tidal deformation [53, 54], can place important constrain on the density of the Martian core. In our study, based on the available density models, we simply estimate the density jump at the ICB (Supplementary Fig. 1) to simultaneously match the mean planetary density (3.935 ± 0.0012 g/cm^3) and mean normalized moment of inertia (MOI) of Mars (0.3634 ± 0.00006). We first compute the mean density ($\bar{\rho}$) and MOI (I), following the integral formula,

$$I = \frac{8\pi}{3} \int \rho(r) r^4 dr, \quad (1)$$

$$\bar{\rho} = \frac{3}{R^3} \int \rho(r) r^2 dr, \quad (2)$$

where r is the radius of an interior layer and ρ is the density of this layer. R is the radius of Mars.

Take an example of MSL_ETH model, we perturb the density at the CMB but maintain the same density gradient as that in the reference model. With a fixed IC size of 610 km, the mean planetary density and MOI are recalculated following Equations 1-2 for each combination of the perturbed CMB density and density jump at the ICB. As shown in Supplementary Fig. 48, to preserve the mean density, a heavier IC would induce a density decrease in the OC. For the reference model of MSL_ETH, a density jump less than ~10 % at the ICB can fit both the density and MOI measurements, considering their respective uncertainties.

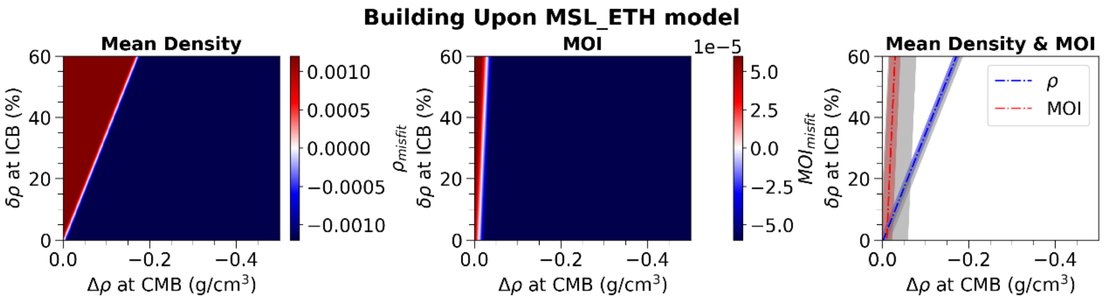

Supplementary Fig. 48. Estimation on density jump at the ICB building upon the MSL_ETH density model. From left to right: the mean planetary density misfit between calculations and geodesy measurement, the moment of inertia (MOI) misfit between calculations and geodesy measurement, and the best fitted lines for the mean density (blue) and MOI (red) and their associated uncertainties. For MSL_ETH density model, the uncertainties from 1,000 inversion models provided by Khan et al. (2023) [3] are also computed, as the grey shaded regions shown. To simultaneously fit the observed density and MOI, a density jump less than ~10 % at the ICB is preferred.

7 Composition and dynamics of the Martian core

7.1 Mineral composition

Considering the IC with a radius of 600 km, temperature at the Martian ICB (with pressure of ~35 GPa) is estimated to be at 1800–2100 K [55-58]. Here, we focus on a Martian core with a sulfur content of 6.6–12 wt.% [59, 60]. Under these conditions, a solid Fe-Ni alloy is expected to crystallize from the (Fe, Ni) + S liquid, descending to the center of Mars to form the solid inner core. This process leaves Fe_3S liquid in the outer core [61].

We then model the compressional-wave velocity (V_P) and density jumps across the ICB with a S content of 10.6 wt.% at 35 GPa and 2000 K. The Fe-Ni alloy is in the face-center cubic (fcc) structure in the Martian inner core [62]. Since Ni has a minor influence on the velocity and density of Fe-Ni alloy, we ignore the contribution of Ni in our following modeling [63]. Here we calculated the V_P and density of fcc Fe-Ni alloy using the experimental results of Kantor et al. (2007) [64], yielding a V_P of 6.16 km/s and density of 9.05 g/cm^3 at 35 GPa and 2000 K. In comparison, our inverted IC has a velocity of 7.3–8.3 km/s at the ICB and a density of 7.2–7.7 g/cm^3, assuming a 7% density jump at the ICB based on the OC density in the MSL_ETH model [3]. Thus, the Fe-Ni alloy exhibits a much lower V_P and significantly higher density than observations.

We further calculate the velocity and density jump across the ICB assuming a liquid Fe_3S OC and Fe-Ni alloy IC (Supplementary Table 9). The thermal equation of state of liquid Fe_3S was measured at ~2000 K up to 52 GPa in Kawaguchi et al. (2017) [65], and varying the temperature has a minor influence on the density of liquid Fe_3S. The bulk modulus of liquid Fe or Fe_3S was calculated following:

$$K_S = -V \times \frac{dP}{dV}, \quad (3)$$

where V is the volume, and P is pressure. The velocity was calculated as

$$V_P = \sqrt{\frac{K_S}{\rho}}, \quad (4)$$

where K_S is the adiabatic bulk modulus, and ρ is density.

In this scenario, when the Martian core has no other light elements except for S, the V_P and density jumps across the Martian ICB are 4.6% and 17.0%, respectively. These values are much lower than our observed V_P of ~30% and higher than density jump of ~7 %. Lowering the ICB temperature to1800 K can slightly increase the V_P jump to 7.8% and lower the density jump to 16.9%, which still cannot satisfy our observed changes across the Martian ICB.

Supplementary Table 9. Thermal elastic parameters of Fe and Fe alloy

	K_{T0} (GPa)	K_{T0}'	V_0 (Å^3)	ρ_0(g/cm^3)	θ_0 (K)	γ_0	q
Fe_3S-Liquid [65][a]	96.1(27)	4.00(13)	--	5.62(91)	--	--	--
Fe-Liquid [66][b]	109.7	4.66	--	7.019	--	--	--

[a]at 2000 K; [b]at 1800 K.

As clarified in **Methods**, using the parameters listed in Supplementary Table 10, our calculations demonstrate that under Martian ICB conditions (35 GPa, 2000 K), if the IC is carbon-enriched, the density and velocity jumps at the boundary would be

approximately 20–27% and ~22% (Supplementary Fig. 49), respectively. These values are inconsistent with our seismological constraints. In contrast, for the second model with an O-enriched inner core, the density jump is reduced to 3-9%, while the velocity jump remains 24-31%, both of which align well with our seismic observations (Extended Data Fig. 8). The velocity and density of the outer core at the ICB are 5.45-6.01 km/s and 6.08-6.48 g/cm³, respectively, while those of the inner core are ~7.86 km/s and 6.68 g/cm³. The resulting density and velocity jumps of 3-9% and 24-31% are consistent with our seismic observations.

Supplementary Table 10. Parameters used in our modeling

	K_{T0} (GPa)	K_{T0}'	V_0 (Å³)	θ_0 (K)	γ_0	q
Fe_3C	311(17)	3.4(1)	148.8(10)	314(fixed)	1.06(42)	1.92(173)
FeO	149(1)	3.60(4)	20.36(fixed)	417(fixed)	1.41(5)	0.5(fixed)

* In the model, densities and seismic velocities of solid FeO and Fe_3C were from Fischer et al. (2011) [67] and Takahashi et al. (2019) [68].

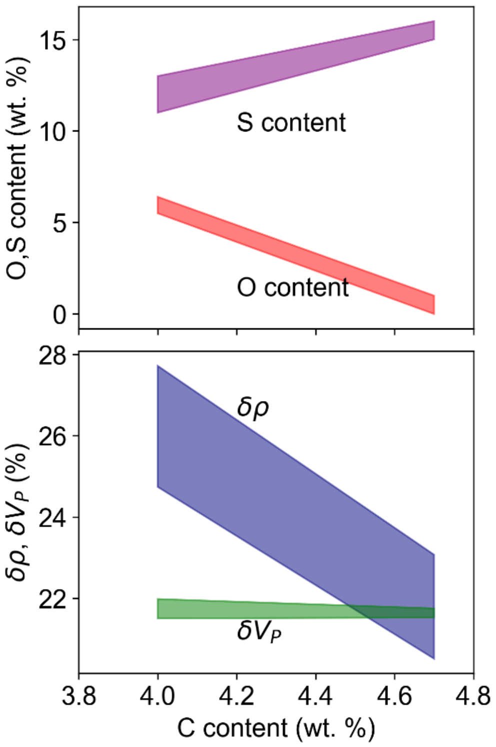

Supplementary Fig. 49. Influence of light elements on the density and velocity jumps across the ICB assuming a C-enriched Martian core. From up to bottom: Amount of S, O, and C in the Martian core used in our modeling; Influence of C content on the density and velocity jumps across the ICB.

7.2 Implications for dynamics

The presence of a solid Martian IC would imply efficient core cooling if the core was very hot in the past. Efficient core cooling also necessitates a robust mantle heat transfer mechanism like large-scale mantle convection [69, 70]. This could result in thermal convection in the core if heat flow across the CMB exceeds the rate at which heat can be conducted out adiabatically. In the presence of vigorous thermal convection, the core should produce a global dynamo field. Indeed, evidence of crustal

magnetization suggests that a dynamo operated early in the history of Mars [71-73] before eventually shutting down, likely due to the gradual reduction in CMB heat flow as the core-mantle temperature difference decreased [57, 69, 74].

Even with diminishing CMB heat flow, however, the core would continue to cool and could eventually begin to crystallize. Under certain conditions, core crystallization, and the resulting compositional convection, is another mechanism that can drive a dynamo [75, 76]. In the top-down (iron snow) regime [77], this requires the crystals to re-melt and mix into the underlying liquid and requires the crystal melt to be denser than the liquid below [78]. In the bottom-up (Earth-like) IC growth regime, core crystallization could drive a dynamo if the IC grows rapidly and if the light element partitioning between the solid and liquid phases results in a residual fluid that is buoyant with respect to the rest of the OC [74, 75]. For example, Morard et al. (2022) [79] report a liquid FeO density of 5.94 g/cm^3 at 39 GPa and 4000 K, lower than OC density at the ICB inferred from the SKS_GD (6.3–6.75 g/cm^3) [2] and MSL_ETH (6.77–7.11 g/cm^3) [3] models. In such a case, the residual fluid after FeO crystallization would be denser than the surrounding core and unlikely to drive convection. However, large uncertainties in Martian core density estimates preclude a definitive assessment. Whether these conditions can be met depends on many factors related to core chemistry and the core's thermal evolution, which in turn depends on heat transport through the mantle and the uncertain initial conditions. Hence, the absence of a strong present-day Martian global dynamo field does not necessarily require the absence of ongoing core crystallization. Finally, if the mantle becomes too sluggish to remove heat from the core, core crystallization would stop, and a dynamo would no longer be possible.

Although a full thermal evolution model is beyond the scope of the present work, our results are broadly consistent with previously considered scenarios in which the Martian core initially cooled rapidly but is now cooling too slowly to drive thermal convection and, in spite of past or even present crystallization, is unable to drive a dynamo due to some combination of core crystallization proceeding too slowly (or not at all) or a lack of density contrast associated with the formation (or melting) of crystals in the core [57, 69, 74]. Further understanding the formation of the Martian IC and its implications for dynamo evolution requires more detailed modeling and improved knowledge of Martian core composition as well as mantle viscosity. Such investigations will be important not only for clarifying Martian interior dynamics but also for understanding dynamo generation in other planetary bodies like Mercury and Ganymede.

References

1. S. C. Stähler, A. Khan, W. B. Banerdt, P. Lognonné, D. Giardini, S. Ceylan, M. Drilleau, A. C. Duran, R. F. Garcia, Q. Huang, D. Kim, V. Lekic, H. Samuel, M. Schimmel, N. Schmerr, D. Sollberger, É. Stutzmann, Z. Xu, D. Antonangeli, C. Charalambous, P. M. Davis, J. C. E. Irving, T. Kawamura, M. Knapmeyer, R. Maguire, A. G. Marusiak, M. P. Panning, C. Perrin, A.-C. Plesa, A. Rivoldini, C. Schmelzbach, G. Zenhäusern, É. Beucler, J. Clinton, N. Dahmen, M. van Driel, T. Gudkova, A. Horleston, W. T. Pike, M. Plasman, and S. E. Smrekar. Seismic detection of the martian core. *Science*, *373*(6553), 443-448 (2021), https://doi.org/10.1126/science.abi7730
2. J. C. E. Irving, V. Lekić, C. Durán, M. Drilleau, D. Kim, A. Rivoldini, A. Khan,

H. Samuel, D. Antonangeli, W. B. Banerdt, C. Beghein, E. Bozdağ, S. Ceylan, C. Charalambous, J. Clinton, P. Davis, R. Garcia, G. Domenico, A. C. Horleston, Q. Huang, K. J. Hurst, T. Kawamura, S. D. King, M. Knapmeyer, J. Li, P. Lognonné, R. Maguire, M. P. Panning, A.-C. Plesa, M. Schimmel, N. C. Schmerr, S. C. Stähler, E. Stutzmann, and Z. Xu. First observations of core-transiting seismic phases on Mars. *Proceedings of the National Academy of Sciences*, *120*(18), e2217090120 (2023), https://doi.org/10.1073/pnas.2217090120

3. A. Khan, D. Huang, C. Durán, P. A. Sossi, D. Giardini, and M. Murakami. Evidence for a liquid silicate layer atop the Martian core. *Nature*, *622*(7984), 718-723 (2023), https://doi.org/10.1038/s41586-023-06586-4
4. H. Samuel, M. Drilleau, A. Rivoldini, Z. Xu, Q. Huang, R. F. Garcia, V. Lekić, J. C. E. Irving, J. Badro, P. H. Lognonné, J. A. D. Connolly, T. Kawamura, T. Gudkova, and W. B. Banerdt. Geophysical evidence for an enriched molten silicate layer above Mars's core. *Nature*, *622*(7984), 712-717 (2023), https://doi.org//10.1038/s41586-023-0693
5. A. S. Konopliv, R. S. Park, A. Rivoldini, R. M. Baland, S. Le Maistre, T. Van Hoolst, M. Yseboodt, and V. Dehant. Detection of the Chandler Wobble of Mars From Orbiting Spacecraft. *Geophysical Research Letters*, *47*(21) (2020), https://doi.org/10.1029/2020GL090568
6. H. P. Crotwell, T. J. Owens, and J. Ritsema. The TauP Toolkit: Flexible Seismic Travel-time and Ray-path Utilities. *Seismological Research Letters*, *70*(2), 154-160 (1999), https://doi.org/10.1785/gssrl.70.2.154
7. T. Nissen-Meyer, M. van Driel, S. C. Stähler, K. Hosseini, S. Hempel, L. Auer, A. Colombi, and A. Fournier. AxiSEM: broadband 3-D seismic wavefields in axisymmetric media. *Solid Earth*, *5*(1), 425-445 (2014), https://doi.org/10/f56h99
8. P. Lognonné, W. B. Banerdt, W. T. Pike, D. Giardini, U. Christensen, R. F. Garcia, T. Kawamura, S. Kedar, B. Knapmeyer-Endrun, L. Margerin, F. Nimmo, M. Panning, B. Tauzin, J. R. Scholz, D. Antonangeli, S. Barkaoui, E. Beucler, F. Bissig, N. Brinkman, M. Calvet, S. Ceylan, C. Charalambous, P. Davis, M. van Driel, M. Drilleau, L. Fayon, R. Joshi, B. Kenda, A. Khan, M. Knapmeyer, V. Lekic, J. McClean, D. Mimoun, N. Murdoch, L. Pan, C. Perrin, B. Pinot, L. Pou, S. Menina, S. Rodriguez, C. Schmelzbach, N. Schmerr, D. Sollberger, A. Spiga, S. Stähler, A. Stott, E. Stutzmann, S. Tharimena, R. Widmer-Schnidrig, F. Andersson, V. Ansan, C. Beghein, M. Böse, E. Bozdag, J. Clinton, I. Daubar, P. Delage, N. Fuji, M. Golombek, M. Grott, A. Horleston, K. Hurst, J. Irving, A. Jacob, J. Knollenberg, S. Krasner, C. Krause, R. Lorenz, C. Michaut, R. Myhill, T. Nissen-Meyer, J. ten Pierick, A. C. Plesa, C. Quantin-Nataf, J. Robertsson, L. Rochas, M. Schimmel, S. Smrekar, T. Spohn, N. Teanby, J. Tromp, J. Vallade, N. Verdier, C. Vrettos, R. Weber, D. Banfield, E. Barrett, M. Bierwirth, S. Calcutt, N. Compaire, C. L. Johnson, D. Mance, F. Euchner, L. Kerjean, G. Mainsant, A. Mocquet, J. A. Rodriguez Manfredi, G. Pont, P. Laudet, T. Nebut, S. de Raucourt, O. Robert, C. T. Russell, A. Sylvestre-Baron, S. Tillier, T. Warren, M. Wieczorek, C. Yana and P. Zweifel. Constraints on the shallow elastic and anelastic structure of Mars from InSight seismic data. *Nature Geoscience*, *13*(3), 213-220 (2020), https://doi.org/10/ggnks8
9. N. Brinkman, S. C. Stähler, D. Giardini, C. Schmelzbach, A. Khan, A. Jacob, N. Fuji, C. Perrin, P. Lognonné, E. Beucler, M. Böse, S. Ceylan, C. Charalambous, J. F. Clinton, M. van Driel, F. Euchner, A. Horleston, T. Kawamura, B.

Knapmeyer‐Endrun, G. Mainsant, M. P. Panning, W. T. Pike, J. R. Scholz, J. O. A. Robertsson, and W. B. Banerdt. First Focal Mechanisms of Marsquakes. *Journal of Geophysical Research: Planets*, *126*(4) (2021), https://doi.org/10/gqzs87

10. R. Maguire, V. Lekić, D. Kim, N. Schmerr, J. Li, C. Beghein, Q. Huang, J. C. E. Irving, F. Karakostas, P. Lognonné, S. C. Stähler, and W. B. Banerdt. Focal Mechanism Determination of Event S1222a and Implications for Tectonics Near the Dichotomy Boundary in Southern Elysium Planitia, Mars. *Journal of Geophysical Research: Planets*, *128*(9), e2023JE007793 (2023), https://doi.org/10/gs4v9t
11. C. Perrin, A. Jacob, A. Lucas, R. Myhill, E. Hauber, A. Batov, T. Gudkova, S. Rodriguez, P. Lognonné, J. Stevanović, M. Drilleau, and N. Fuji. Geometry and Segmentation of Cerberus Fossae, Mars: Implications for Marsquake Properties. *Journal of Geophysical Research: Planets*, *127*(1) (2022), https://doi.org/10/gqzs84
12. G. Zenhäusern, S. C. Stähler, J. F. Clinton, D. Giardini, S. Ceylan, and R. F. Garcia. Low‐Frequency Marsquakes and Where to Find Them: Back Azimuth Determination Using a Polarization Analysis Approach. *Bulletin of the Seismological Society of America*, *112*(4), 1787-1805 (2022), https://doi.org/10/gr4gdv
13. M. Böse, D. Giardini, S. Stähler, S. Ceylan, J. F. Clinton, M. van Driel, A. Khan, F. Euchner, P. Lognonné, and W. B. Banerdt. Magnitude Scales for Marsquakes. *Bulletin of the Seismological Society of America*, *108*(5A), 2764-2777 (2018), https://doi.org/10/gfcv88
14. S. Ceylan, J. F. Clinton, D. Giardini, S. C. Stähler, A. Horleston, T. Kawamura, M. Böse, C. Charalambous, N. L. Dahmen, M. van Driel, C. Durán, F. Euchner, A. Khan, D. Kim, M. Plasman, J.-R. Scholz, G. Zenhäusern, E. Beucler, R. F. Garcia, S. Kedar, M. Knapmeyer, P. Lognonné, M. P. Panning, C. Perrin, W. T. Pike, A. E. Stott, and W. B. Banerdt. The marsquake catalogue from InSight, sols 0–1011. *Physics of the Earth and Planetary Interiors*, *333*, 106943 (2022), https://doi.org/10/gtd2zt
15. M. Drilleau, H. Samuel, R. F. Garcia, A. Rivoldini, C. Perrin, C. Michaut, M. Wieczorek, B. Tauzin, J. A. D. Connolly, P. Meyer, P. Lognonné, and W. B. Banerdt. Marsquake Locations and 1‐D Seismic Models for Mars From InSight Data. *Journal of Geophysical Research: Planets*, *127*(9) (2022), https://doi.org/10/gqzs83
16. M. Sita and S. van der Lee. Potential Volcano-Tectonic Origins and Faulting Mechanisms of Three Low-Frequency Marsquakes Detected by a Single InSight Seismometer. *Journal of Geophysical Research: Planets*, *127*(10), e2022JE007309 (2022), https://doi.org/10/gtd2zr
17. T. Kawamura, J. F. Clinton, G. Zenhäusern, S. Ceylan, A. C. Horleston, N. L. Dahmen, C. Duran, D. Kim, M. Plasman, S. C. Stähler, F. Euchner, C. Charalambous, D. Giardini, P. Davis, G. Sainton, P. Lognonné, M. Panning, and W. B. Banerdt. S1222a - the largest Marsquake detected by InSight. *Geophysical Research Letters*, *n/a*(n/a), e2022GL101543 https://doi.org/10/grhn4k
18. D. Giardini, P. Lognonné, W. B. Banerdt, W. T. Pike, U. Christensen, S. Ceylan, J. F. Clinton, M. van Driel, S. C. Stähler, M. Böse, R. F. Garcia, A. Khan, M. Panning, C. Perrin, D. Banfield, E. Beucler, C. Charalambous, F. Euchner, A.

Horleston, A. Jacob, T. Kawamura, S. Kedar, G. Mainsant, J. R. Scholz, S. E. Smrekar, A. Spiga, C. Agard, D. Antonangeli, S. Barkaoui, E. Barrett, P. Combes, V. Conejero, I. Daubar, M. Drilleau, C. Ferrier, T. Gabsi, T. Gudkova, K. Hurst, F. Karakostas, S. King, M. Knapmeyer, B. Knapmeyer-Endrun, R. Llorca-Cejudo, A. Lucas, L. Luno, L. Margerin, J. B. McClean, D. Mimoun, N. Murdoch, F. Nimmo, M. Nonon, C. Pardo, A. Rivoldini, J. A. R. Manfredi, H. Samuel, M. Schimmel, A. E. Stott, E. Stutzmann, N. Teanby, T. Warren, R. C. Weber, M. Wieczorek, and C. Yana. The seismicity of Mars. *Nature Geoscience*, *13*(3), 205-212 (2020), https://doi.org/10/gg27cg

19. C. Durán, A. Khan, S. Ceylan, G. Zenhäusern, S. Stähler, J. F. Clinton, and D. Giardini. Seismology on Mars: An analysis of direct, reflected, and converted seismic body waves with implications for interior structure. *Physics of the Earth and Planetary Interiors*, *325*, 106851 (2022), https://doi.org/10.1016/j.pepi.2022.106851

20. B. Fernando, I. J. Daubar, C. Charalambous, P. M. Grindrod, A. Stott, A. Al Ateqi, D. Atri, S. Ceylan, J. Clinton, M. Fillingim, E. Hauber, J. R. Hill, T. Kawamura, J. Liu, A. Lucas, R. Lorenz, L. Ojha, C. Perrin, S. Piqueux, S. Stähler, D. Tirsch, C. Wilson, N. Wójcicka, D. Giardini, P. Lognonné, and W. B. Banerdt. A Tectonic Origin for the Largest Marsquake Observed by InSight. *Geophysical Research Letters*, *50*(20), e2023GL103619 (2023), https://doi.org/10/gsv8cs

21. S. C. Stähler, A. Mittelholz, C. Perrin, T. Kawamura, D. Kim, M. Knapmeyer, G. Zenhäusern, J. Clinton, D. Giardini, P. Lognonné, and W. B. Banerdt. Tectonics of Cerberus Fossae unveiled by marsquakes. *Nature Astronomy*, *6*(12), 1376-1386 (2022), https://doi.org/10/gq497j

22. A. C. Horleston, J. F. Clinton, S. Ceylan, D. Giardini, C. Charalambous, J. C. E. Irving, P. Lognonné, S. C. Stähler, G. Zenhäusern, N. L. Dahmen, C. Duran, T. Kawamura, A. Khan, D. Kim, M. Plasman, F. Euchner, C. Beghein, É. Beucler, Q. Huang, M. Knapmeyer, B. Knapmeyer-Endrun, V. Lekić, J. Li, C. Perrin, M. Schimmel, N. C. Schmerr, A. E. Stott, E. Stutzmann, N. A. Teanby, Z. Xu, M. Panning, and W. B. Banerdt. The Far Side of Mars: Two Distant Marsquakes Detected by InSight. *The Seismic Record*, *2*(2), 88-99 (2022), https://doi.org/10.1785/0320220007

23. M. P. Panning, W. B. Banerdt, C. Beghein, S. Carrasco, S. Ceylan, J. F. Clinton, P. Davis, M. Drilleau, D. Giardini, A. Khan, D. Kim, B. Knapmeyer-Endrun, J. Li, P. Lognonné, S. C. Stähler, and G. Zenhäusern. Locating the Largest Event Observed on Mars With Multi-Orbit Surface Waves. *Geophysical Research Letters*, *50*(1), e2022GL101270 (2023), https://doi.org/10.1029/2022GL101270

24. M. Drilleau, É. Beucler, P. Lognonné, M. P. Panning, B. Knapmeyer-Endrun, W. B. Banerdt, C. Beghein, S. Ceylan, M. van Driel, R. Joshi, T. Kawamura, A. Khan, S. Menina, A. Rivoldini, H. Samuel, S. Stähler, H. Xu, M. Bonnin, J. Clinton, D. Giardini, B. Kenda, V. Lekic, A. Mocquet, N. Murdoch, M. Schimmel, S. E. Smrekar, É. Stutzmann, B. Tauzin, and S. Tharimena. MSS/1: Single-Station and Single-Event Marsquake Inversion. *Earth and Space Science*, *7*(12), e2020EA001118 (2020), https://doi.org/10/gtd56m

25. M. Böse, J. F. Clinton, S. Ceylan, F. Euchner, M. van Driel, A. Khan, D. Giardini, P. Lognonné, and W. B. Banerdt. A probabilistic framework for single-station location of seismicity on Earth and Mars. *Physics of the Earth and Planetary Interiors*, *262*, 48-65 (2017), https://doi.org/10/f9qptz

26. A. Khan, M. van Driel, M. Böse, D. Giardini, S. Ceylan, J. Yan, J. Clinton, F. Euchner, P. Lognonné, N. Murdoch, D. Mimoun, M. Panning, M. Knapmeyer, and W. B. Banerdt. Single-station and single-event marsquake location and inversion for structure using synthetic Martian waveforms. *Physics of the Earth and Planetary Interiors*, *258*, 28-42 (2016), https://doi.org/10/f82tr5
27. J. Clinton, D. Giardini, M. Böse, S. Ceylan, M. van Driel, F. Euchner, R. F. Garcia, S. Kedar, A. Khan, S. C. Stähler, B. Banerdt, P. Lognonne, E. Beucler, I. Daubar, M. Drilleau, M. Golombek, T. Kawamura, M. Knapmeyer, B. Knapmeyer-Endrun, D. Mimoun, A. Mocquet, M. Panning, C. Perrin, and N. A. Teanby. The Marsquake Service: Securing Daily Analysis of SEIS Data and Building the Martian Seismicity Catalogue for InSight. *Space Science Reviews*, *214*(8), 133 (2018), https://doi.org/10/gtd58w
28. InSight Marsquake Service, "Mars Seismic Catalogue, InSight Mission; V14 2023-04-01" (ETHZ, IPGP, JPL, ICL, Univ. Bristol, 2023); https://doi.org:10.12686/a17
29. J. F. Clinton, S. Ceylan, M. van Driel, D. Giardini, S. C. Stähler, M. Böse, C. Charalambous, N. L. Dahmen, A. Horleston, T. Kawamura, A. Khan, G. Orhand-Mainsant, J.-R. Scholz, F. Euchner, W. B. Banerdt, P. Lognonné, D. Banfield, E. Beucler, R. F. Garcia, S. Kedar, M. P. Panning, C. Perrin, W. T. Pike, S. E. Smrekar, A. Spiga, and A. E. Stott. The Marsquake catalogue from InSight, sols 0–478. *Physics of the Earth and Planetary Interiors*, *310* (2021), https://doi.org/10.1016/j.pepi.2020.106595
30. F.-C. Lin, V. C. Tsai, B. Schmandt, Z. Duputel, and Z. Zhan. Extracting seismic core phases with array interferometry. *Geophysical Research Letters*, *40*(6), 1049-1053 (2013), https://doi.org/10.1002/grl.50237
31. J. Zhang, X. Song, Y. Li, P. G. Richards, X. Sun, and F. Waldhauser. Inner Core Differential Motion Confirmed by Earthquake Waveform Doublets. *Science*, *309*(5739), 1357-1360 (2005), https://doi.org/10.1126/science.1113193
32. C. Ammon, T. Lay, and D. Simpson. Great Earthquakes and Global Seismic Networks. *Seismological Research Letters*, *81*, 965-971 (2010), https://doi.org/10.1785/gssrl.81.6.965
33. S. M. Mousavi, Y. Sheng, W. Zhu, and G. C. Beroza. STanford EArthquake Dataset (STEAD): A Global Data Set of Seismic Signals for AI. *IEEE Access*, *7*, 179464-179476 (2019), https://doi.org/10.1109/ACCESS.2019.2947848
34. K. Obara, K. Kasahara, S. Hori, and Y. Okada. A densely distributed high-sensitivity seismograph network in Japan: Hi-net by National Research Institute for Earth Science and DisasterPrevention. *Review of scientific instruments*, *76*(2) (2005), https://doi.org/10.1063/1.1854197
35. Q. Huang, N. C. Schmerr, S. D. King, D. Kim, A. Rivoldini, A.-C. Plesa, H. Samuel, R. R. Maguire, F. Karakostas, and V. Lekić. Seismic detection of a deep mantle discontinuity within Mars by InSight. *Proceedings of the National Academy of Sciences*, *119*(42), e2204474119 (2022), https://doi.org/10.1073/pnas.2204474119
36. A. Khan, S. Ceylan, M. van Driel, D. Giardini, P. Lognonné, H. Samuel, N. C. Schmerr, S. C. Stähler, A. C. Duran, Q. Huang, D. Kim, A. Broquet, C. Charalambous, J. F. Clinton, P. M. Davis, M. Drilleau, F. Karakostas, V. Lekic, S. M. McLennan, R. R. Maguire, C. Michaut, M. P. Panning, W. T. Pike, B. Pinot, M. Plasman, J.-R. Scholz, R. Widmer-Schnidrig, T. Spohn, S. E. Smrekar, and W. B. Banerdt. Upper mantle structure of Mars from InSight seismic data. *Science*, *373*(6553), 434-438 (2021), https://doi.org/10.1126/science.abf2966

37. D. Kim, W. B. Banerdt, S. Ceylan, D. Giardini, V. Lekić, P. Lognonné, C. Beghein, É. Beucler, S. Carrasco, C. Charalambous, J. Clinton, M. Drilleau, C. Durán, M. Golombek, R. Joshi, A. Khan, B. Knapmeyer-Endrun, J. Li, R. Maguire, W. T. Pike, H. Samuel, M. Schimmel, N. C. Schmerr, S. C. Stähler, E. Stutzmann, M. Wieczorek, Z. Xu, A. Batov, E. Bozdag, N. Dahmen, P. Davis, T. Gudkova, A. Horleston, Q. Huang, T. Kawamura, S. D. King, S. M. McLennan, F. Nimmo, M. Plasman, A. C. Plesa, I. E. Stepanova, E. Weidner, G. Zenhäusern, I. J. Daubar, B. Fernando, R. F. Garcia, L. V. Posiolova, and M. P. Panning. Surface waves and crustal structure on Mars. *Science*, *378*(6618), 417-421 (2022), https://doi.org/10.1126/science.abq715
38. B. Bolt. The constitution of the core: seismological evidence. *Philosophical Transactions of the Royal Society of London. Series A, Mathematical and Physical Sciences*, *306*(1492), 11-20 (1982), https://doi.org/10.1098/rsta.1982.0062
39. R. Jeanloz. The nature of the Earth's core. *Annual Review of Earth and Planetary Sciences*, *18*(1), 357-386 (1990), https://doi.org/10.1146/annurev.ea.18.050190.002041
40. M. D. Kohler and T. Tanimoto. One-layer global inversion for outermost core velocity. *Physics of the Earth and Planetary Interiors*, *72*(3), 173-184 (1992), https://doi.org/10/fqg9vg
41. A. M. Dziewonski and D. L. Anderson. Preliminary reference Earth model. *Physics of the earth and planetary interiors*, *25*(4), 297-356 (1981), https://doi.org/10.1016/0031-9201(81)90046-7
42. W. c. Yu, L. Wen, and F. Niu. Seismic velocity structure in the Earth's outer core. *Journal of Geophysical Research: Solid Earth*, *110*(B2) (2005), https://doi.org/10.1029/2003JB002928
43. J. C. Irving, S. Cottaar, and V. Lekić. Seismically determined elastic parameters for Earth's outer core. *Science advances*, *4*(6), eaar2538 (2018), https://doi.org/10.1126/sciadv.aar253
44. J. Badro, A. S. Côté, and J. P. Brodholt. A seismologically consistent compositional model of Earth's core. *Proceedings of the National Academy of Sciences of the United States of America*, *111*(21), 7542-7545 (2014), https://doi.org/10/gqh8qh
45. G. G. Buchbinder. A velocity structure of the Earth's core. *Bulletin of the Seismological Society of America*, *61*(2), 429-456 (1971), https://doi.org/10.1785/BSSA0610020429
46. J. Shlens. A Tutorial on Principal Component Analysis. *arXiv.org*, https://arxiv.org/abs/1404.1100v1 (2014),
47. M. C. Brennan, R. A. Fischer, and J. C. E. Irving. Core formation and geophysical properties of Mars. *Earth and Planetary Science Letters*, *530*, 115923 (2020), https://doi.org/10.1016/j.epsl.2019.115923
48. H. Terasaki, A. Rivoldini, Y. Shimoyama, K. Nishida, S. Urakawa, M. Maki, F. Kurokawa, Y. Takubo, Y. Shibazaki, T. Sakamaki, A. Machida, Y. Higo, K. Uesugi, A. Takeuchi, T. Watanuki, and T. Kondo. Pressure and Composition Effects on Sound Velocity and Density of Core-Forming Liquids: Implication to Core Compositions of Terrestrial Planets. *Journal of Geophysical Research: Planets*, *124*(8), 2272-2293 (2019), https://doi.org/10.1029/2019JE005936
49. D. Kim, P. Davis, V. Lekić, R. Maguire, N. Compaire, M. Schimmel, E. Stutzmann, J. C. E. Irving, P. Lognonné, J. R. Scholz, J. Clinton, G. Zenhäusern, N. Dahmen, S. Deng, A. Levander, M. P. Panning, R. F. Garcia, D. Giardini, K.

Hurst, B. Knapmeyer‐Endrun, F. Nimmo, W. T. Pike, L. Pou, N. Schmerr, S. C. Stähler, B. Tauzin, R. Widmer‐Schnidrig, and W. B. Banerdt. Potential Pitfalls in the Analysis and Structural Interpretation of Seismic Data from the Mars InSight Mission. *Bulletin of the Seismological Society of America*, *111*(6), 2982-3002 (2021), https://doi.org/10.1785/0120210123

50. S. Deng and A. Levander. Autocorrelation Reflectivity of Mars. *Geophysical Research Letters*, *47*(16), e2020GL089630 (2020), https://doi.org/10.1029/2020GL089630
51. S. Deng and A. Levander. Seismic Autocorrelation Analysis of Deep Mars. *Geophysical Research Letters*, *50*(24), e2023GL105046 (2023), https://doi.org/10.1029/2023GL105046
52. S. Wang and H. Tkalčić. Scanning for planetary cores with single-receiver intersource correlations. *Nature Astronomy*, *6*(11), 1272-1279 (2022), https://doi.org/10.1038/s41550-022-01796-8
53. F. Sohl, G. Schubert, and T. Spohn. Geophysical constraints on the composition and structure of the Martian interior. *Journal of Geophysical Research: Planets*, *110*(E12) (2005), https://doi.org/10.1029/2005JE002520
54. C. F. Yoder, A. S. Konopliv, D. N. Yuan, E. M. Standish, and W. M. Folkner. Fluid core size of mars from detection of the solar tide. *Science*, *300*(5617), 299-303 (2003), https://doi.org/10.1126/science.1079645
55. A. Khan, P. A. Sossi, C. Liebske, A. Rivoldini, and D. Giardini. Geophysical and cosmochemical evidence for a volatile-rich Mars. *Earth and Planetary Science Letters*, *578*, 117330 (2022), https://doi.org/10.1016/j.epsl.2021.117330
56. S. A. Hauck II and R. J. Phillips. Thermal and crustal evolution of Mars. *Journal of Geophysical Research: Planets*, *107*(E7), 6-1-6-19 (2002), https://doi.org/10.1029/2001JE001801
57. J.-P. Williams and F. Nimmo. Thermal evolution of the Martian core: Implications for an early dynamo. *Geology*, *32*(2), 97-100 (2004), https://doi.org/10.1130/g19975.1
58. G. Schubert and T. Spohn. Thermal history of Mars and the sulfur content of its core. *Journal of Geophysical Research: Solid Earth*, *95*(B9), 14095-14104 (1990), https://doi.org/10.1029/JB095iB09p14095
59. K. Lodders and B. Fegley. An oxygen isotope model for the composition of Mars. *Icarus*, *126*(2), 373-394 (1997), https://doi.org/10.1006/icar.1996.5653
60. T. Yoshizaki and W. F. McDonough. The composition of Mars. *Geochimica et Cosmochimica Acta*, *273*, 137-162 (2020), https://doi.org/10.1016/j.gca.2020.01.011
61. A. J. Stewart, M. W. Schmidt, W. van Westrenen, and C. Liebske. Mars: A New Core-Crystallization Regime. *Science*, *316*(5829), 1323-1325 (2007), https://doi.org/10.1126/science.1140549
62. T. J. Ahrens, K. G. Holland, and G. Q. Chen. Phase diagram of iron, revised-core temperatures. *Geophysical Research Letters*, *29*(7), 54-51-54-54 (2002), https://doi.org/10.1029/2001gl014350
63. H. K. Mao, Y. Wu, L. C. Chen, J. F. Shu, and A. P. Jephcoat. Static compression of iron to 300 GPa and Fe0.8Ni0.2 alloy to 260 GPa: Implications for composition of the core. *Journal of Geophysical Research: Solid Earth*, *95*(B13), 21737-21742 (1990), https://doi.org/10.1029/JB095iB13p21737
64. A. P. Kantor, I. Y. Kantor, A. V. Kurnosov, A. Y. Kuznetsov, N. A. Dubrovinskaia, M. Krisch, A. A. Bossak, V. P. Dmitriev, V. S. Urusov, and L. S. Dubrovinsky.

Sound wave velocities of fcc Fe–Ni alloy at high pressure and temperature by mean of inelastic X-ray scattering. *Physics of the Earth and Planetary Interiors*, *164*(1), 83-89 (2007), https://doi.org/10.1016/j.pepi.2007.06.006
65. S. I. Kawaguchi, Y. Nakajima, K. Hirose, T. Komabayashi, H. Ozawa, S. Tateno, Y. Kuwayama, S. Tsutsui, and A. Q. R. Baron. Sound velocity of liquid Fe-Ni-S at high pressure. *Journal of Geophysical Research: Solid Earth*, *122*(5), 3624-3634 (2017), https://doi.org/10.1002/2016jb013609
66. W. W. Anderson and T. J. Ahrens. An equation of state for liquid iron and implications for the Earth's core. *Journal of Geophysical Research: Solid Earth*, *99*(B3), 4273-4284 (1994), https://doi.org/10.1029/93JB03158
67. R. A. Fischer, A. J. Campbell, O. T. Lord, G. A. Shofner, P. Dera, and V. B. Prakapenka. Phase transition and metallization of FeO at high pressures and temperatures. *Geophysical Research Letters*, *38*(24) (2011), https://doi.org/10.1029/2011GL049800
68. S. Takahashi, E. Ohtani, T. Sakamaki, S. Kamada, H. Fukui, S. Tsutsui, H. Uchiyama, D. Ishikawa, N. Hirao, Y. Ohishi, and A. Q. R. Baron. Sound velocity of Fe3C at high pressure and high temperature determined by inelastic X-ray scattering. *Comptes Rendus Geoscience*, *351*(2-3), 190-196 (2019), https://doi.org/10.1016/j.crte.2018.09.005
69. D. J. Stevenson. Mars' core and magnetism. *Nature*, *412*(6843), 214-219 (2001), https://doi.org/10.1038/35084155
70. R. E. Young and G. Schubert. Temperatures Inside Mars: Is the core liquid or solid? *Geophysical Research Letters*, *1*, 157-160 (1974), https://doi.org/10.1029/GL001i004p00157
71. M. H. Acuña, J. E. P. Connerney, N. F, Ness, R. P. Lin, D. Mitchell, C. W. Carlson, J. McFadden, K. A. Anderson, H. Rème, C. Mazelle, D. Vignes, P. Wasilewski, and P. Cloutier. Global Distribution of Crustal Magnetization Discovered by the Mars Global Surveyor MAG/ER Experiment. *Science*, *284*(5415), 790-793 (1999), https://doi.org/10.1126/science.284.5415.790
72. J. E. P. Connerney, M. H. Acuña, N. F. Ness, G. Kletetschka, D. L. Mitchell, R. P. Lin, and H. Reme. Tectonic implications of Mars crustal magnetism. *Proceedings of the National Academy of Sciences*, *102*(42), 14970-14975 (2005), https://doi.org/10.1073/pnas.0507469102
73. B. Langlais, E. Thébault, A. Houliez, Michael E. Purucker, and Robert J. Lillis. A New Model of the Crustal Magnetic Field of Mars Using MGS and MAVEN. *Journal of Geophysical Research: Planets*, *124*(6), 1542-1569 (2019), https://doi.org/10.1029/2018JE005854
74. D. J. Hemingway and P. E. Driscoll. History and Future of the Martian Dynamo and Implications of a Hypothetical Solid Inner Core. *Journal of Geophysical Research: Planets*, *126*(4), e2020JE006663 (2021), https://doi.org/10.1029/2020JE006663
75. P. Driscoll and D. Bercovici. On the thermal and magnetic histories of Earth and Venus: Influences of melting, radioactivity, and conductivity. *Physics of the Earth and Planetary Interiors*, *236*, 36-51 (2014), https://doi.org/10.1016/j.pepi.2014.08.004
76. J. R. Lister and B. A. Buffett. The strength and efficiency of thermal and compositional convection in the geodynamo. *Physics of the Earth and Planetary Interiors*, *91*(1-3), 17-30 (1995), https://doi.org/10.1016/0031-9201(95)03042-U
77. D. Breuer, T. Rueckriemen, and T. Spohn. Iron snow, crystal floats, and inner-

core growth: modes of core solidification and implications for dynamos in terrestrial planets and moons. *Progress in Earth and Planetary Science*, *2*(1), 39 (2015), https://doi.org/10.1186/s40645-015-0069-y

78. P. Olson, M. Landeau, and B. Hirsh. Laboratory experiments on rain-driven convection: Implications for planetary dynamos. *Earth and Planetary Science Letters*, *457* (2016), https://doi.org/10.1016/j.epsl.2016.10.015

79. G. Morard, D. Antonangeli, J. Bouchet, A. Rivoldini, S. Boccato, F. Miozzi, E. Boulard, H. Bureau, M. Mezouar, C. Prescher, S. Chariton, and E. Greenberg. Structural and Electronic Transitions in Liquid FeO Under High Pressure. *Journal of Geophysical Research: Solid Earth*, *127*(11), e2022JB025117 (2022), https://doi.org/10.1029/2022JB025117
