# Supplementary material for: Seismic detection of a 600-km solid inner core in Mars
Source: Nature. 2025 Sep 3;645(8079):67–72. doi: 10.1038/s41586-025-09361-9 (PMC12408336; doi:10.1038/s41586-025-09361-9)
Supplement: Supplementary file 2 — This file contains additional information on the individual events analysis for different phases, and inversion results obtained from measurements directly from the vespagrams. [file 41586_2025_9361_MOESM2_ESM.pdf]

Supplementary Information B for

Seismic Detection of a 600-km Solid Inner Core in Mars

Huixing Bi, Daoyuan Sun*, Ningyu Sun, Zhu Mao, Mingwei Dai, Douglas Hemingway

Corresponding author: sdy2014@ustc.edu.cn

The PDF file includes:

1 Overview 2
2 Analysis for individual events 2
    2.1 P 2
    2.2 P′P′r_ab 15
    2.3 P′P′n 19
    2.4 PKKP 22
    2.5 PKiKP 26
3 Inversion results from M_pick method 30

1 Overview

This supplement contains additional information on the individual events analysis for the direct-P, PKKP, P′P′r_ab and P′P′n phase (**Section 3.2.3** in the **Supplementary Information A**), and inversion results obtained from the M_pick method (**Section 4** in the **Supplementary Information A**).

2 Analysis for individual events

In this section, we display the analysis of P-wave and detected core phases, including P′P′r_ab, P′P′n and PKKP, for individual events.

2.1 P

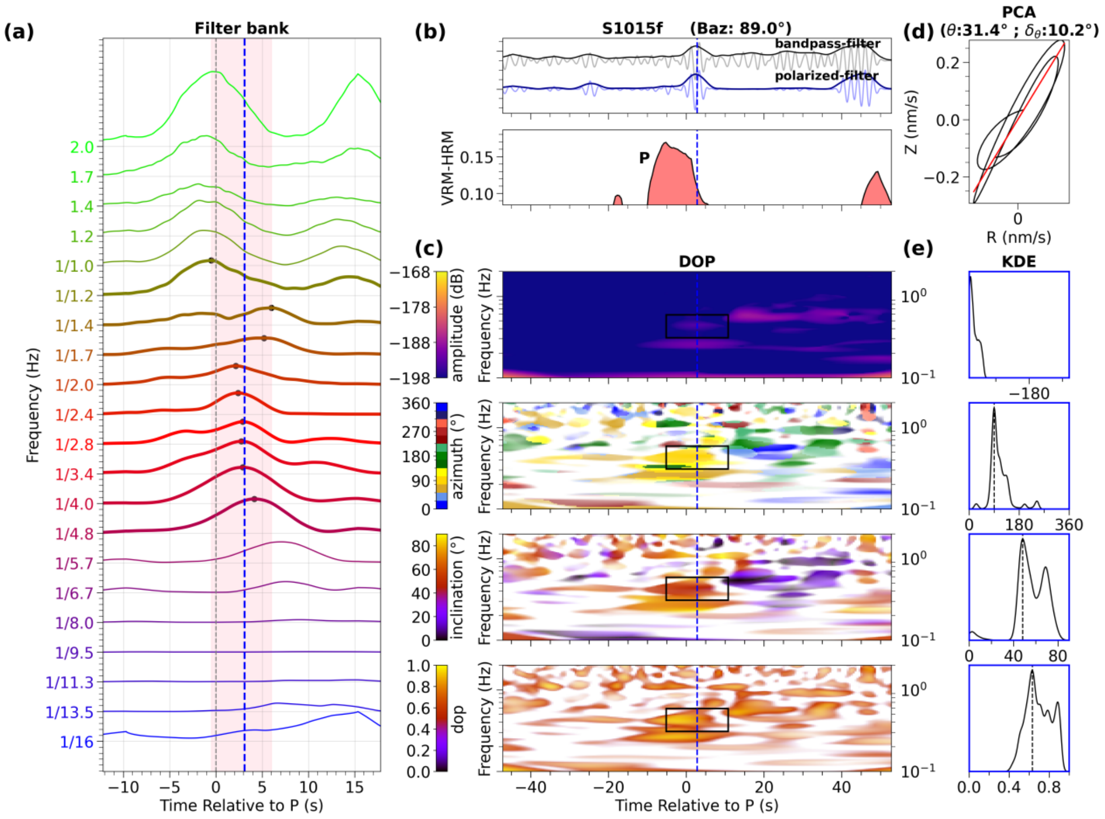

Fig. SS1. Identification of P phase for event S1015f. (a) Filter bank analysis. (b) Waveform and FDPA analysis. The upper panel shows the vertical component waveforms and corresponding envelopes of the bandpass- (grey line) and polarization- (blue line) filtered traces within a frequency band indicated by the black rectangles circled in (c). The back-azimuth is provided by MQS. The lower panel is the vertical-horizontal summed FDPA intensity as a function of time. (c) Polarized analysis. From top to bottom, spectrum, azimuth, inclination angle of the major axis of particle motion, and ellipticity (DOP) obtained from polarization analysis. We reject all parts of the signal

with a DOP < 0.6 by setting the corresponding part of the S-transformed data to zero, which allowed us to suppress some weakly polarized signals. The inclination angle represents the angle deviating from the horizontal plane. The black dashed lines denote the travel-time picks obtained from filter bank analysis. The black rectangles outline the travel-time pick uncertainties of ± 8 s in the frequency range 0.3-0.6 Hz. The blue dashed lines in (b) and (c) denote the travel-time picked from filter bank analysis in (a). (d) The particle motions on the vertical and radial components in a time window of ± 2.5 s based on the travel time picked from filter bank analysis. The red line denotes the fitted line and its incident angle deviating from the vertical plane (θ) and error (δ_θ) are annotated below. (e) Kernel density estimation. The black dashed lines denote the maximum values of probability density computed by marginalizing over the frequency and time axes as the black rectangle shown in (c).

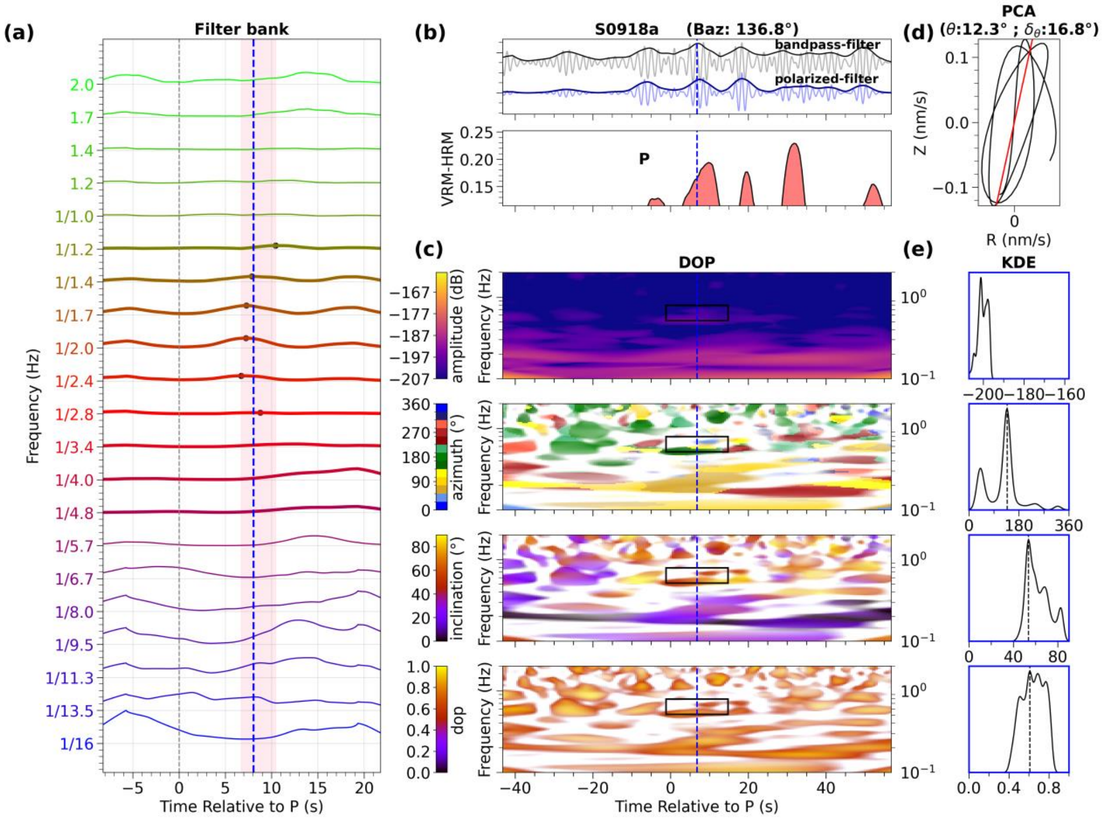

Fig. SS2. Identification of P phase on individual event S0918a, same as Fig. SS1.

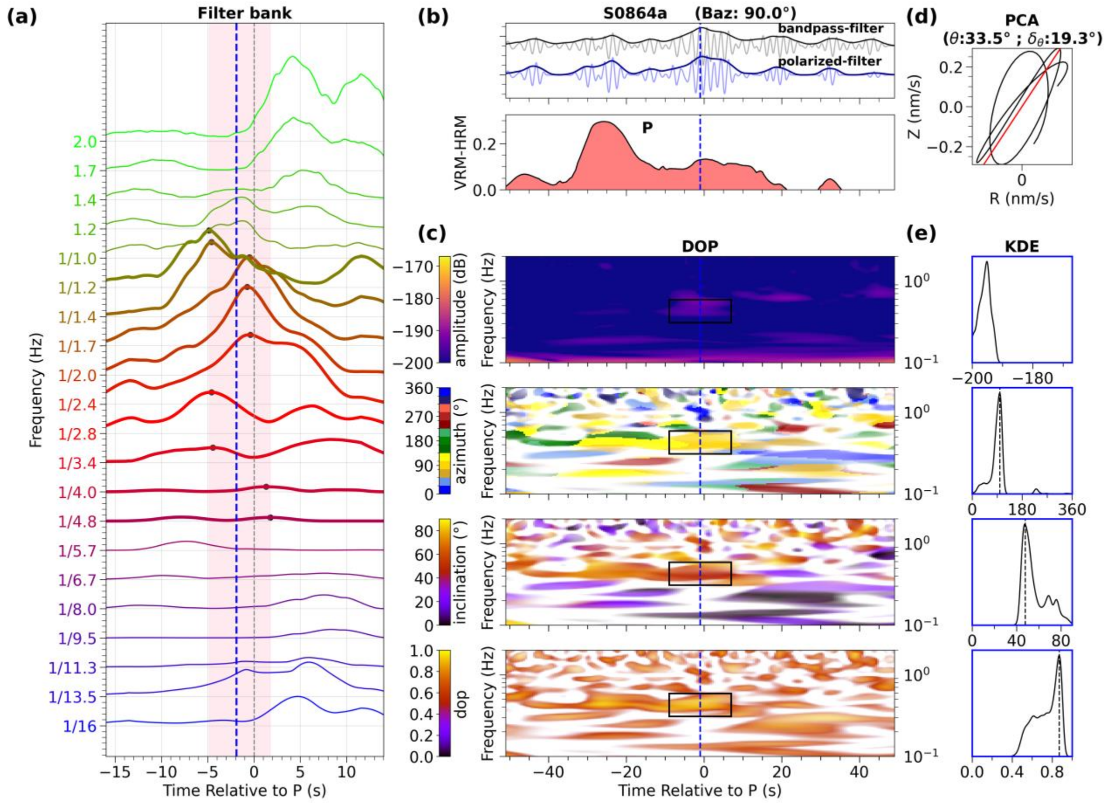

Fig. SS3. Identification of P phase on individual event S0864a, same as Fig. SS1.

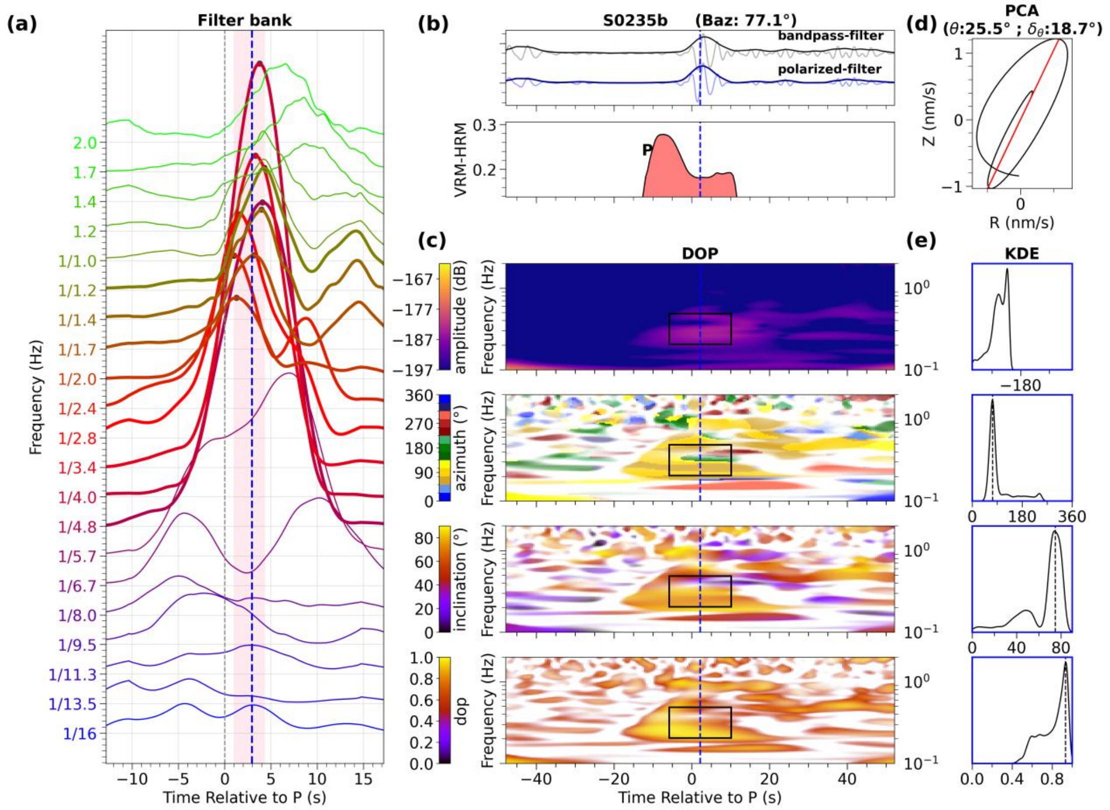

Fig. SS4. Identification of P phase on individual event S0235b, same as Fig. SS1.

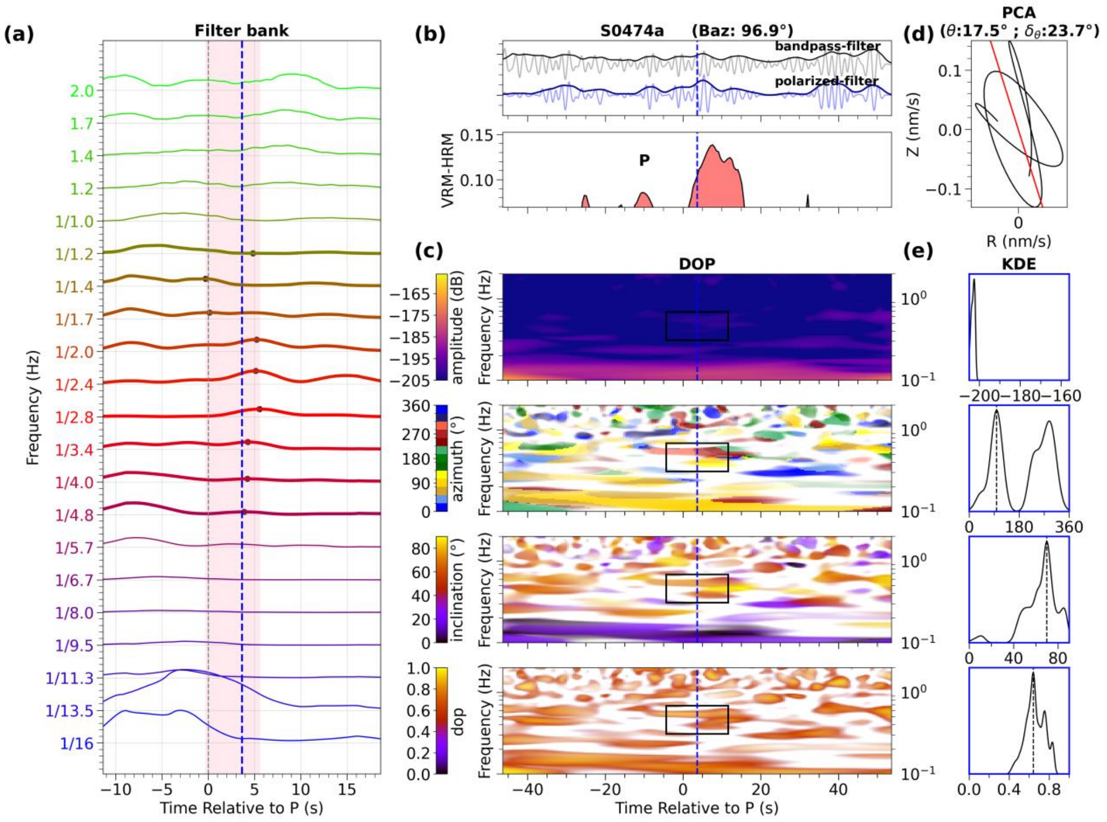

Fig. SS5. Identification of P phase on individual event S0474a, same as Fig. SS1.

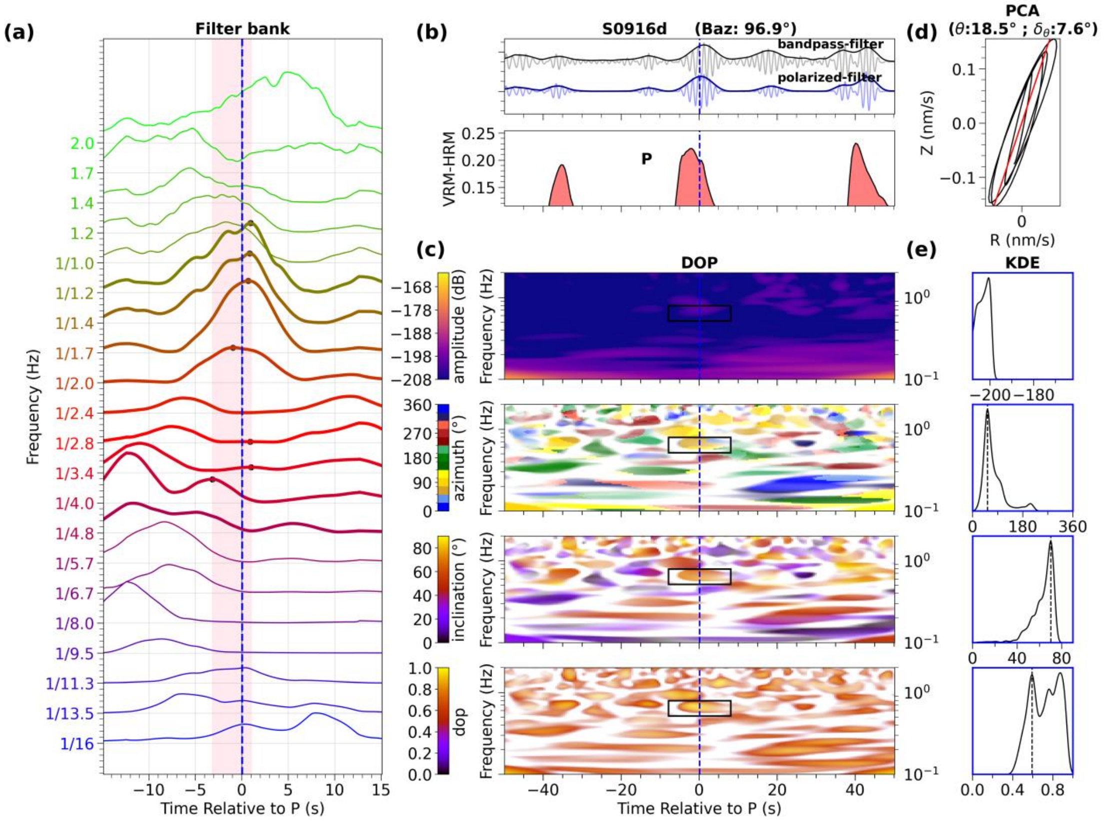

Fig. SS6. Identification of P phase on individual event S0916d, same as Fig. SS1.

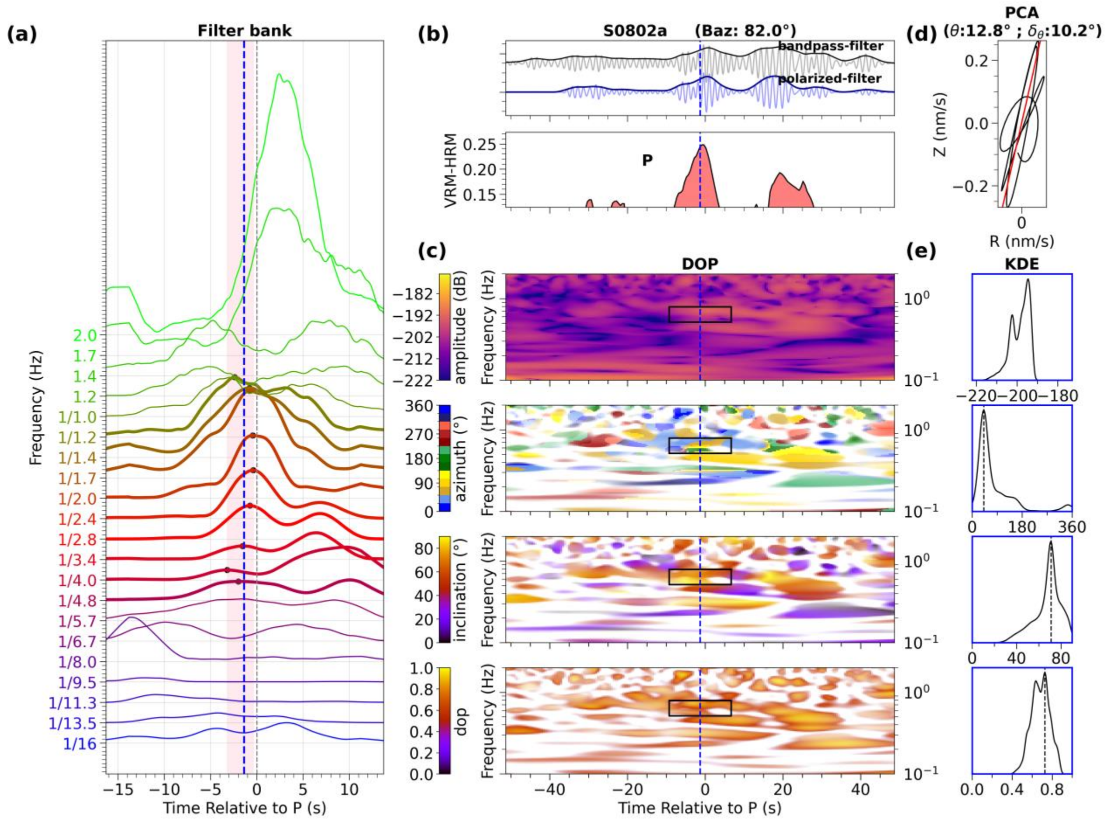

Fig. SS7. Identification of P phase on individual event S0802a, same as Fig. SS1.

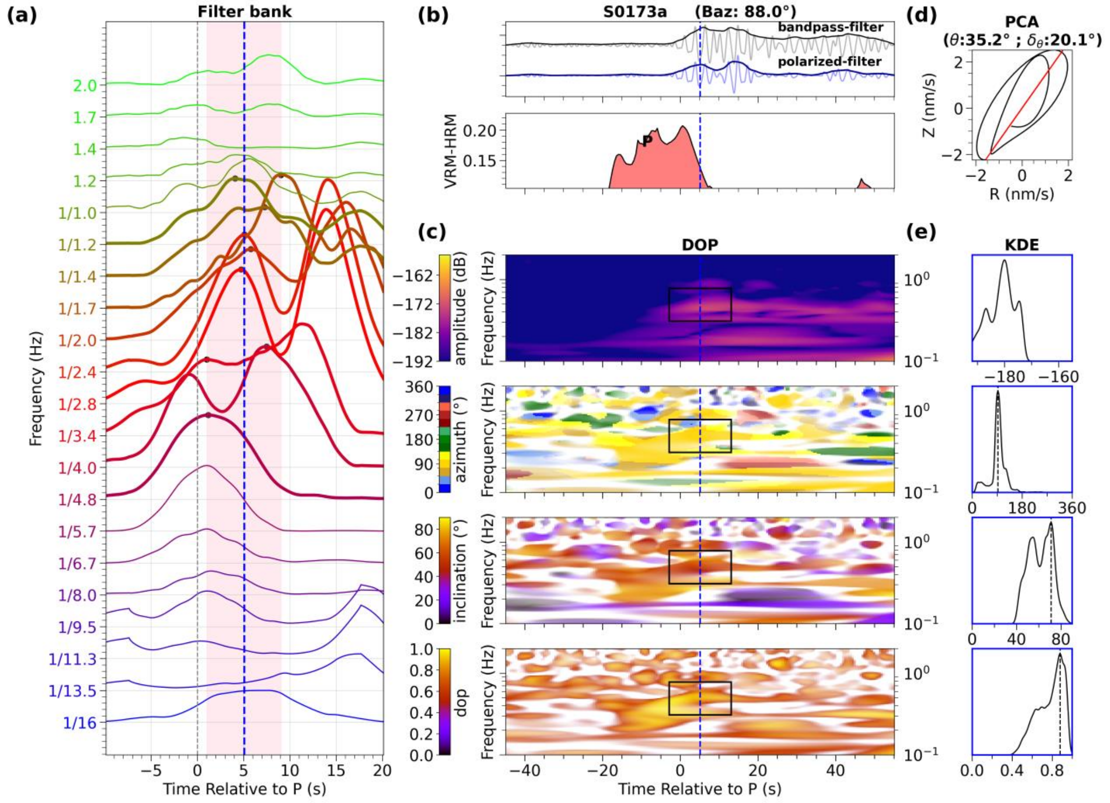

Fig. SS8. Identification of P phase on individual event S0173a, same as Fig. SS1.

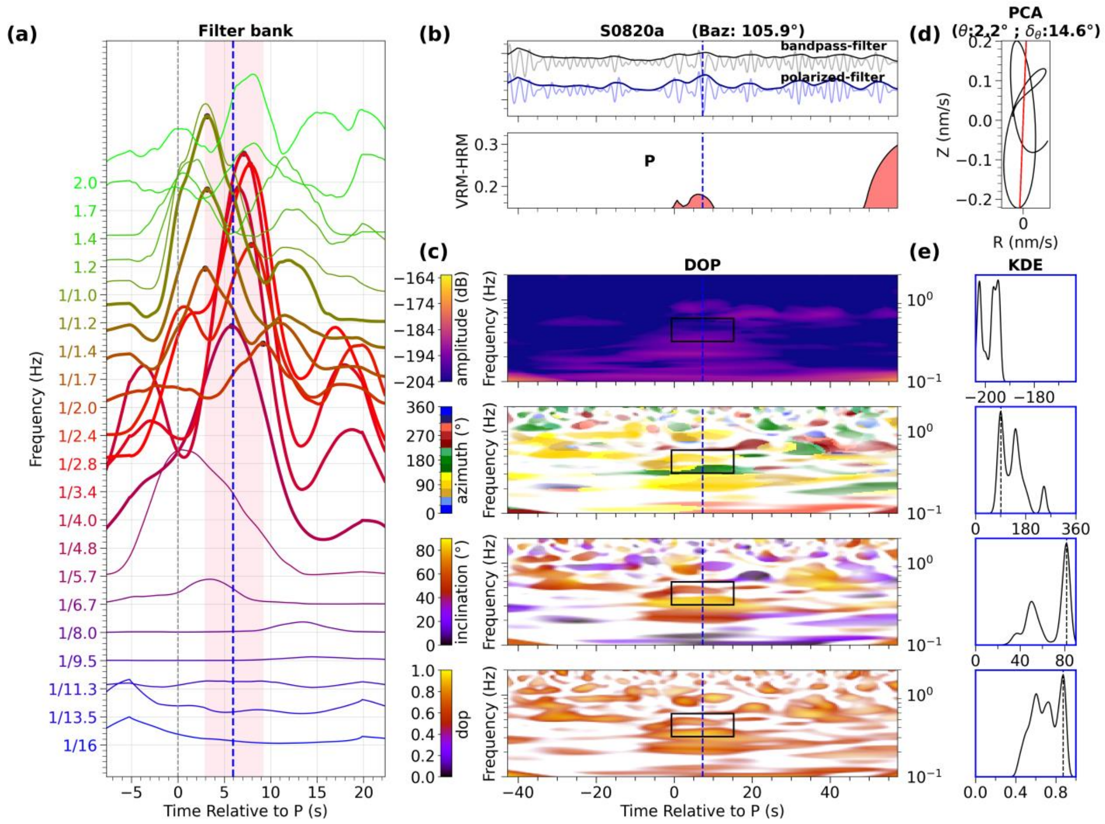

Fig. SS9. Identification of P phase on individual event S0820a, same as Fig. SS1.

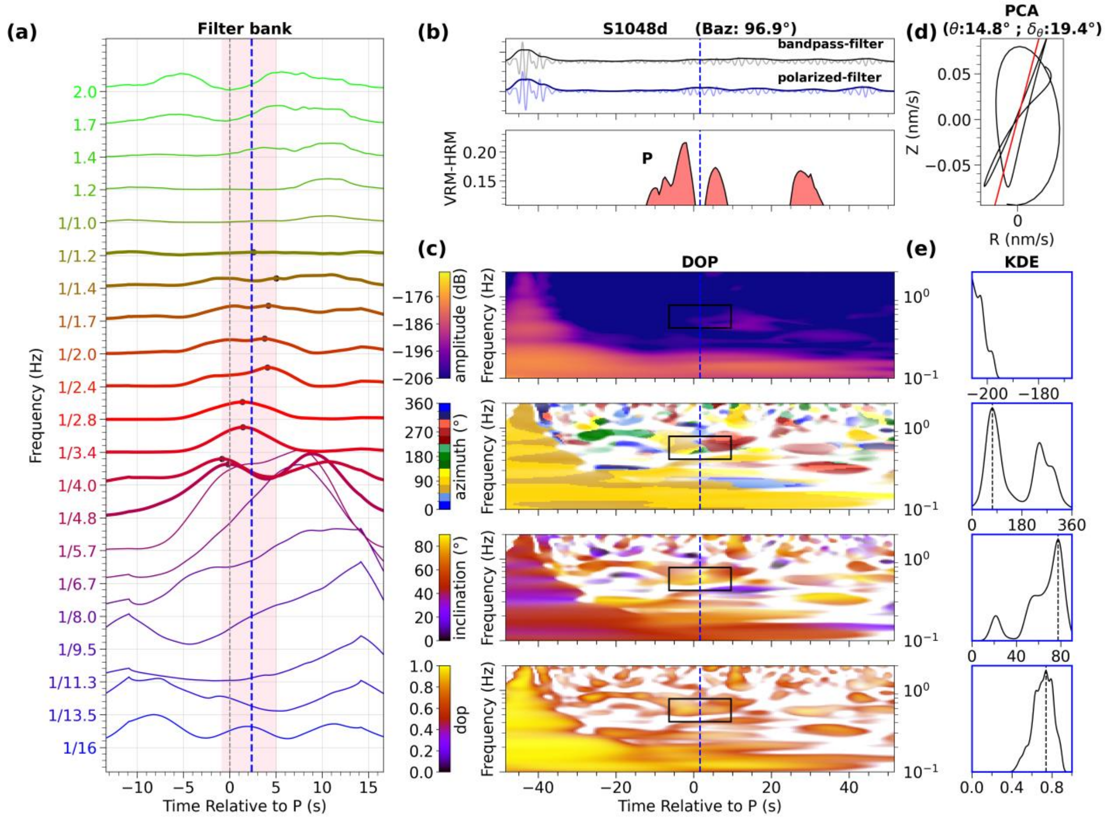

Fig. SS10. Identification of P phase on individual event S1048d, same as Fig. SS1.

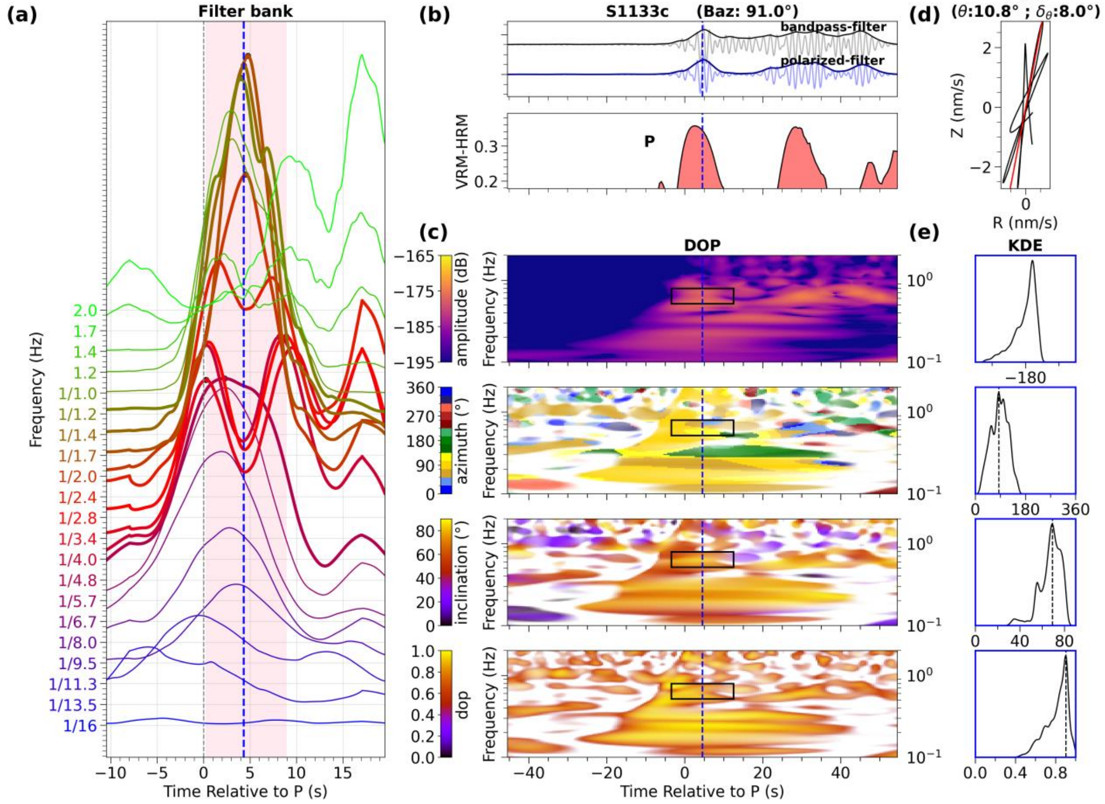

Fig. SS11. Identification of P phase on individual event S1133c, same as Fig. SS1.

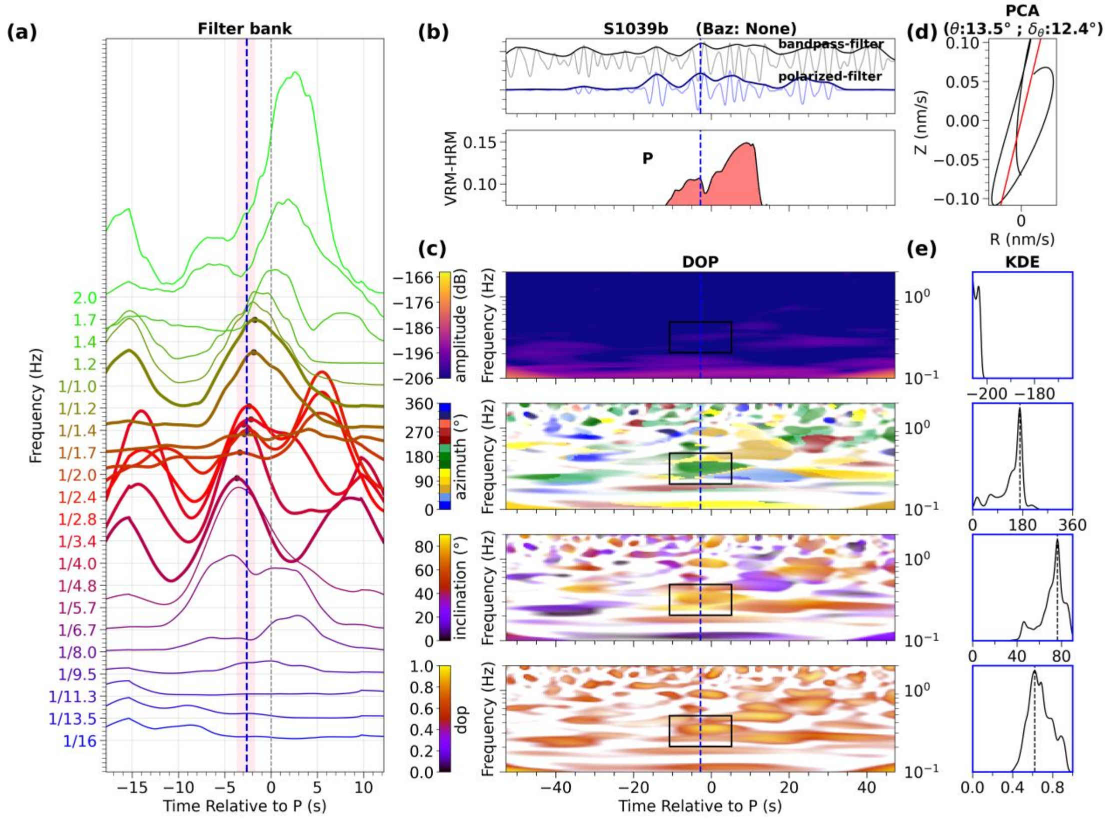

Fig. SS12. Identification of P phase on individual event S1039b, same as Fig. SS1.

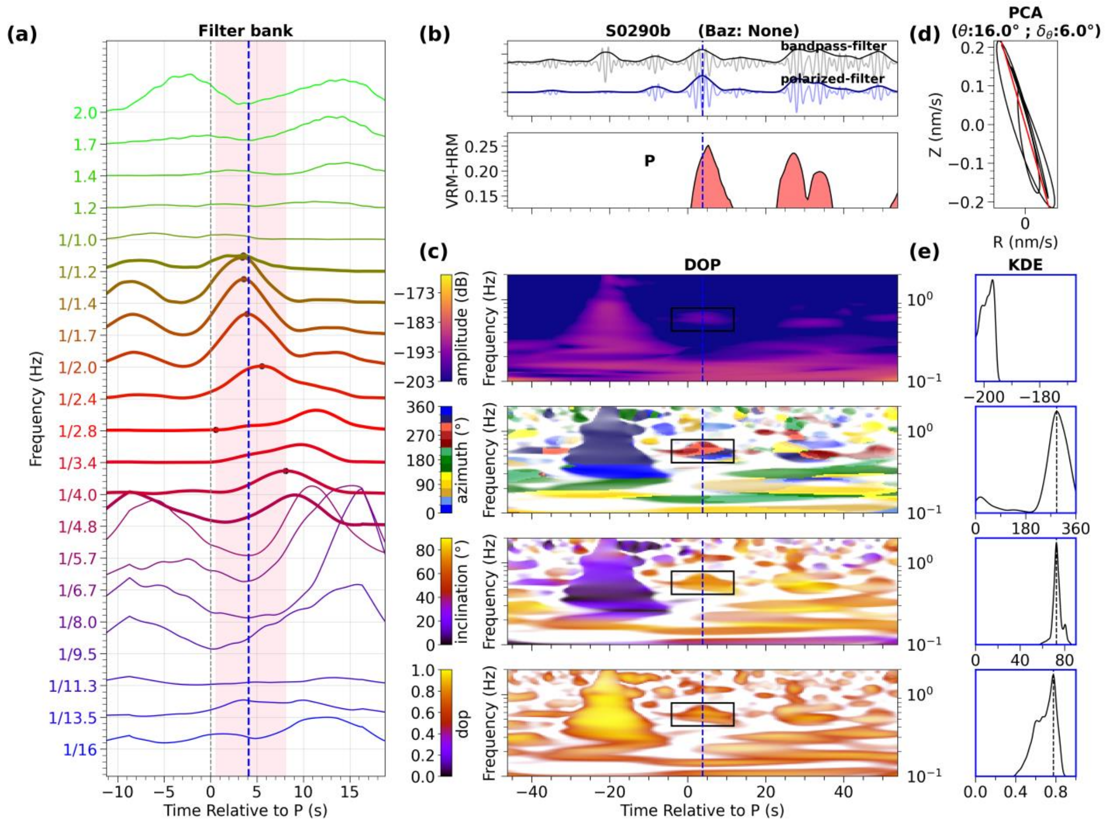

Fig. SS13. Identification of P phase on individual event S0290b, same as Fig. SS1.

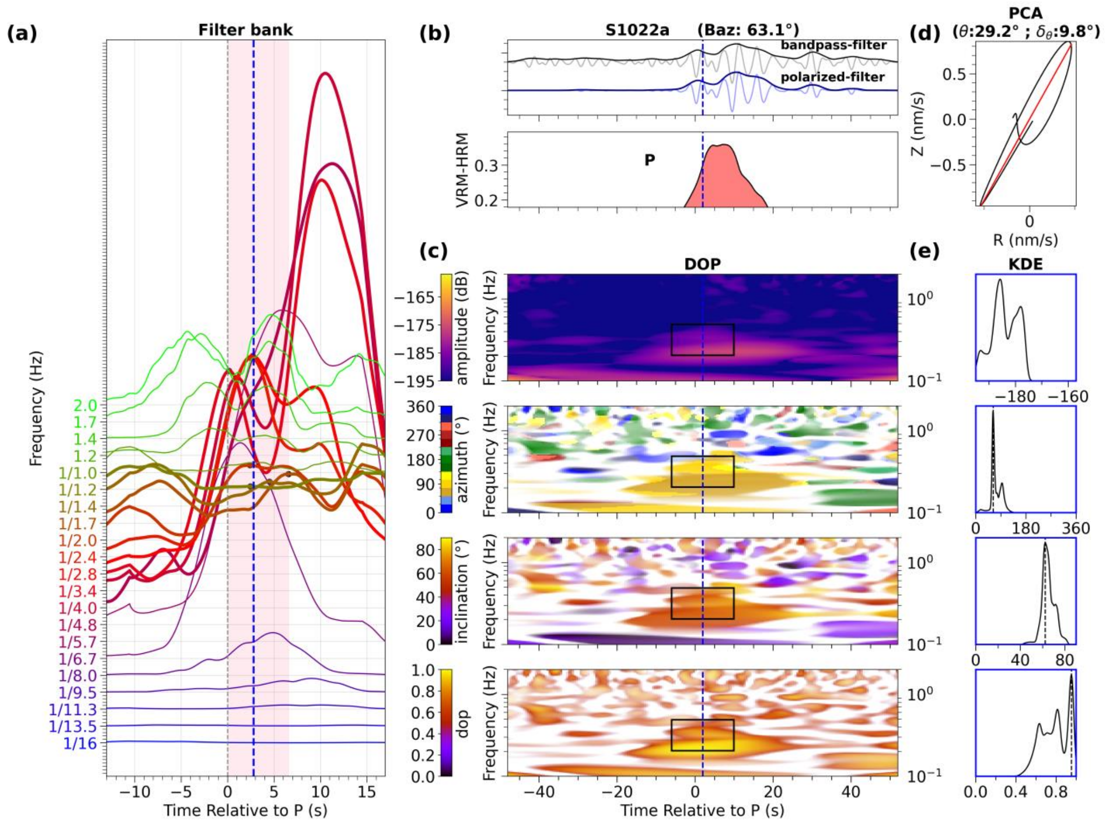

Fig. SS14. Identification of P phase on individual event S1022a, same as Fig. SS1.

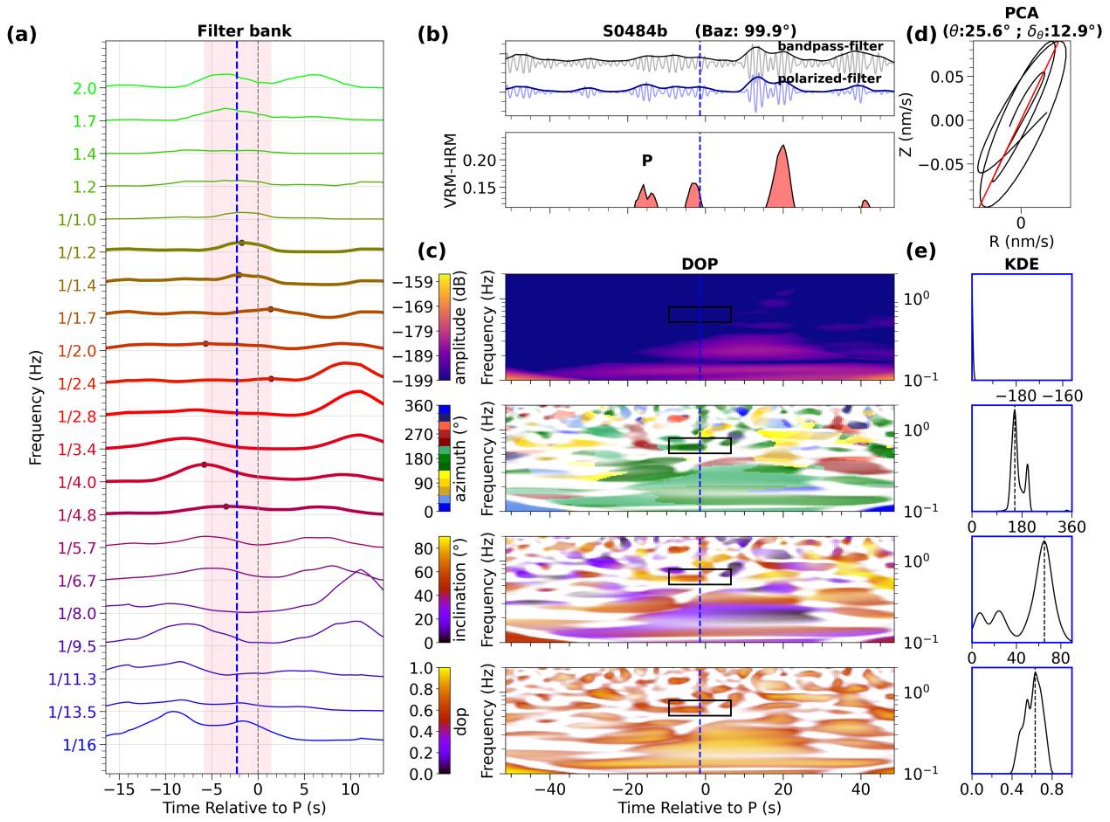

Fig. SS15. Identification of P phase on individual event S0484b, same as Fig. SS1.

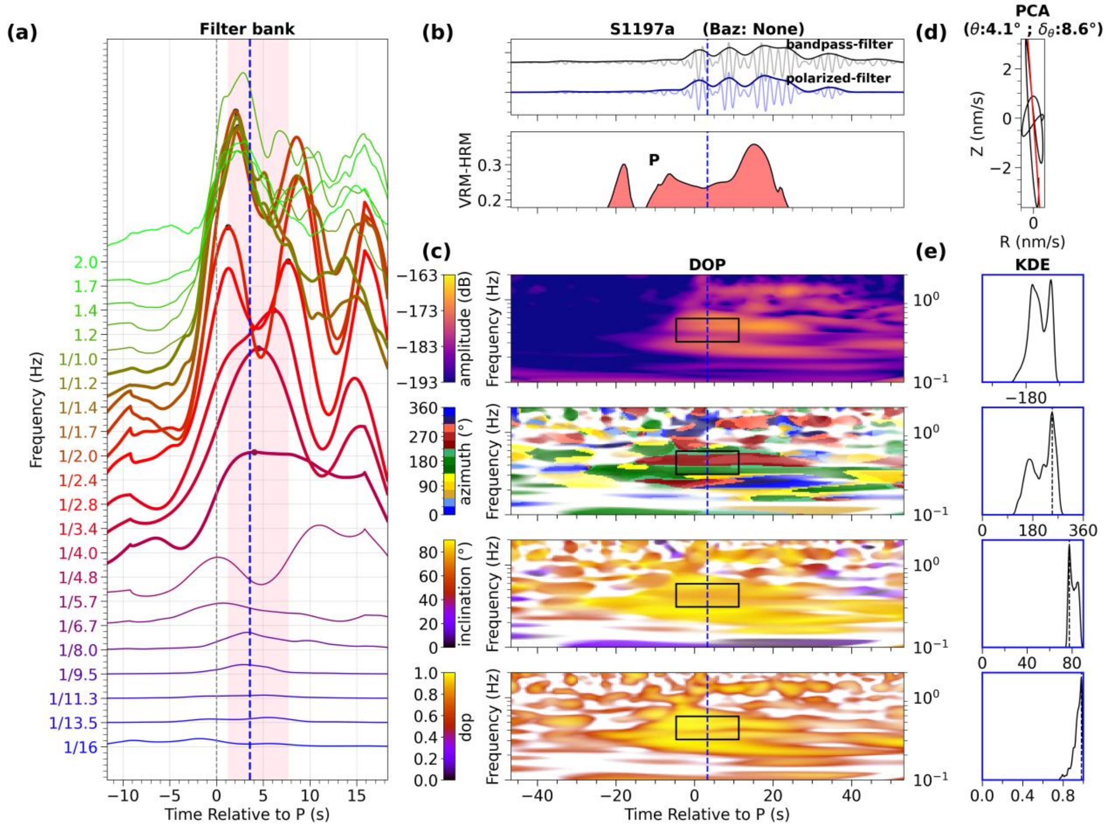

Fig. SS16. Identification of P phase on individual event S1197a, same as Fig. SS1.

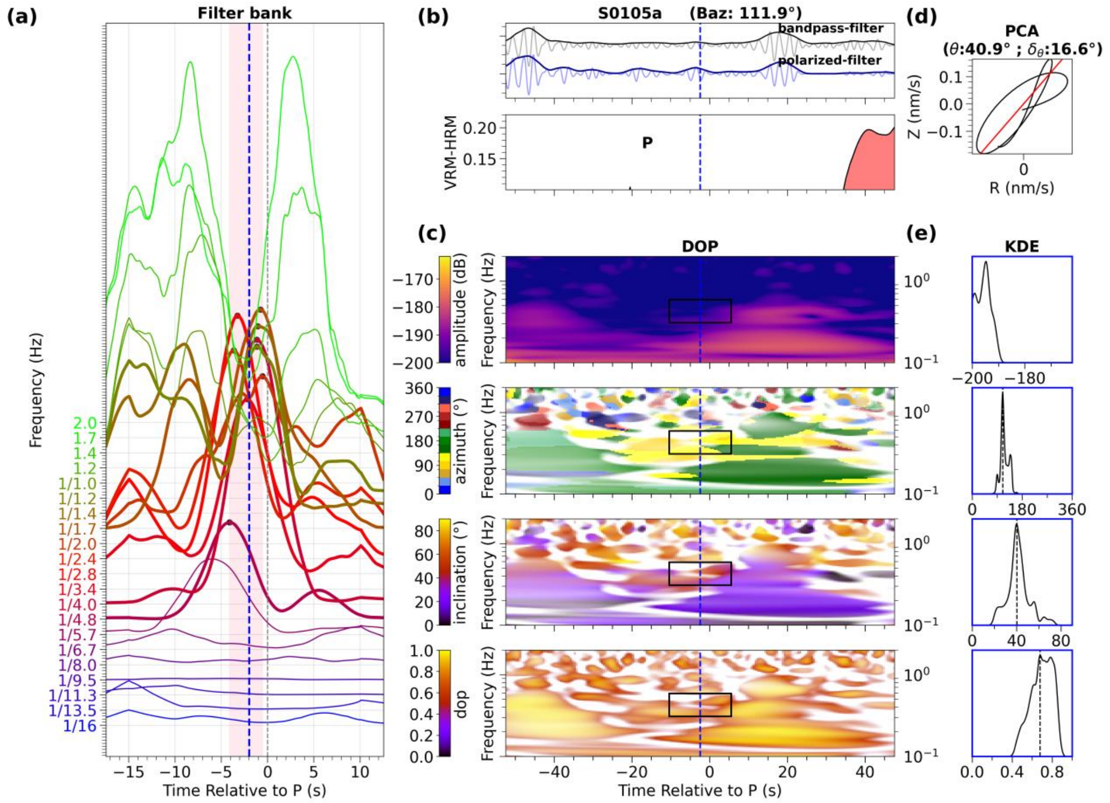

Fig. SS17. Identification of P phase on individual event S0105a, same as Fig. SS1.

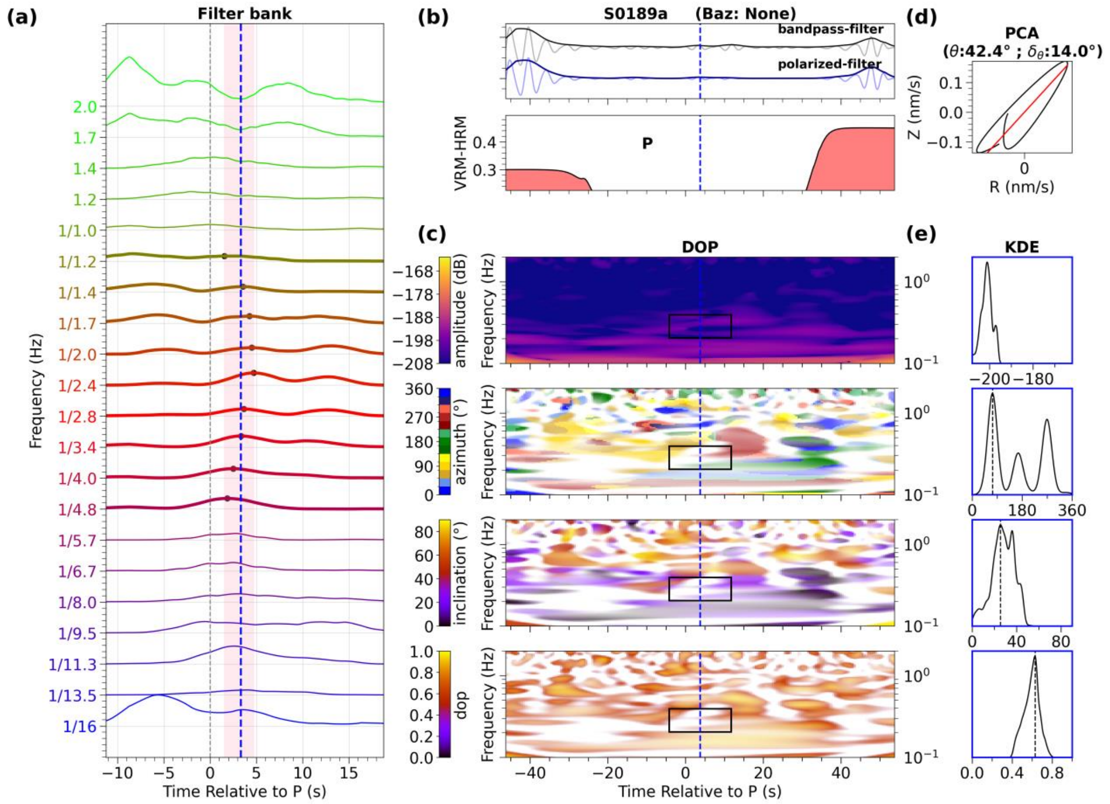

Fig. SS18. Identification of P phase on individual event S0189a, same as Fig. SS1.

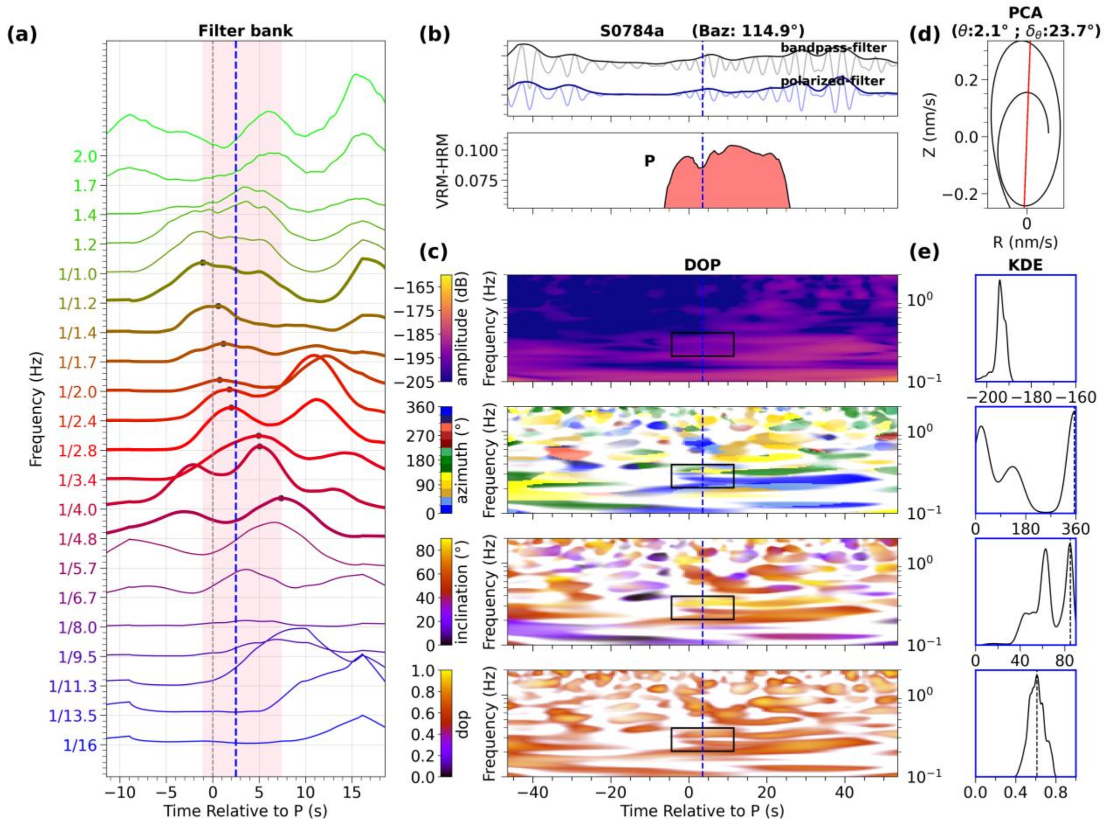

Fig. SS19. Identification of P phase on individual event S0784a, same as Fig. SS1.

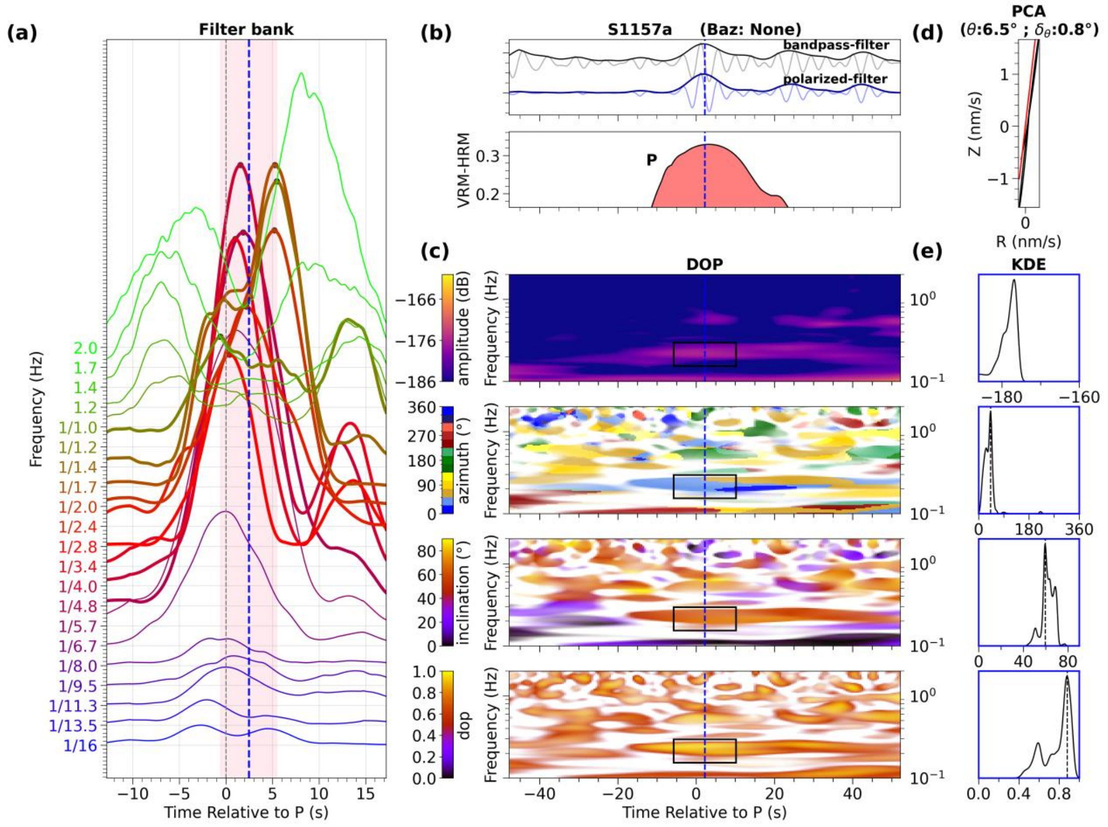

Fig. SS20. Identification of P phase on individual event S1157a, same as Fig. SS1.

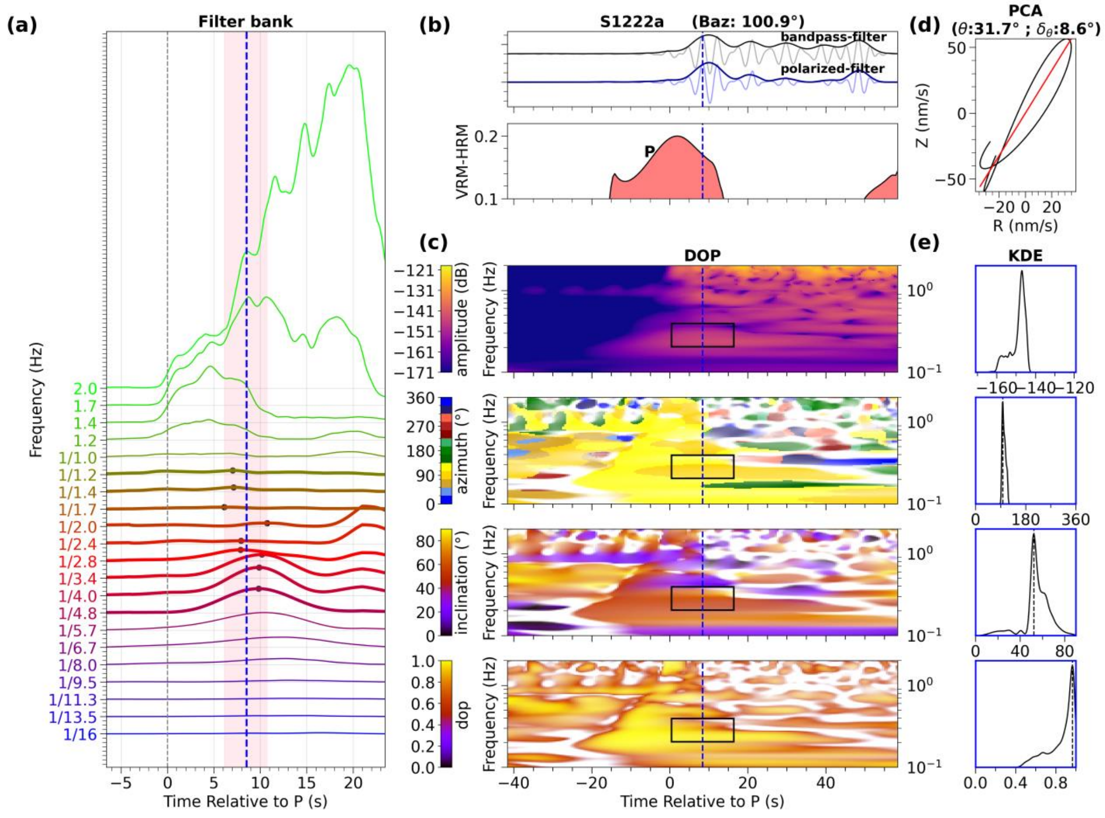

Fig. SS21. Identification of P phase on individual event S1222a, same as Fig. SS1.

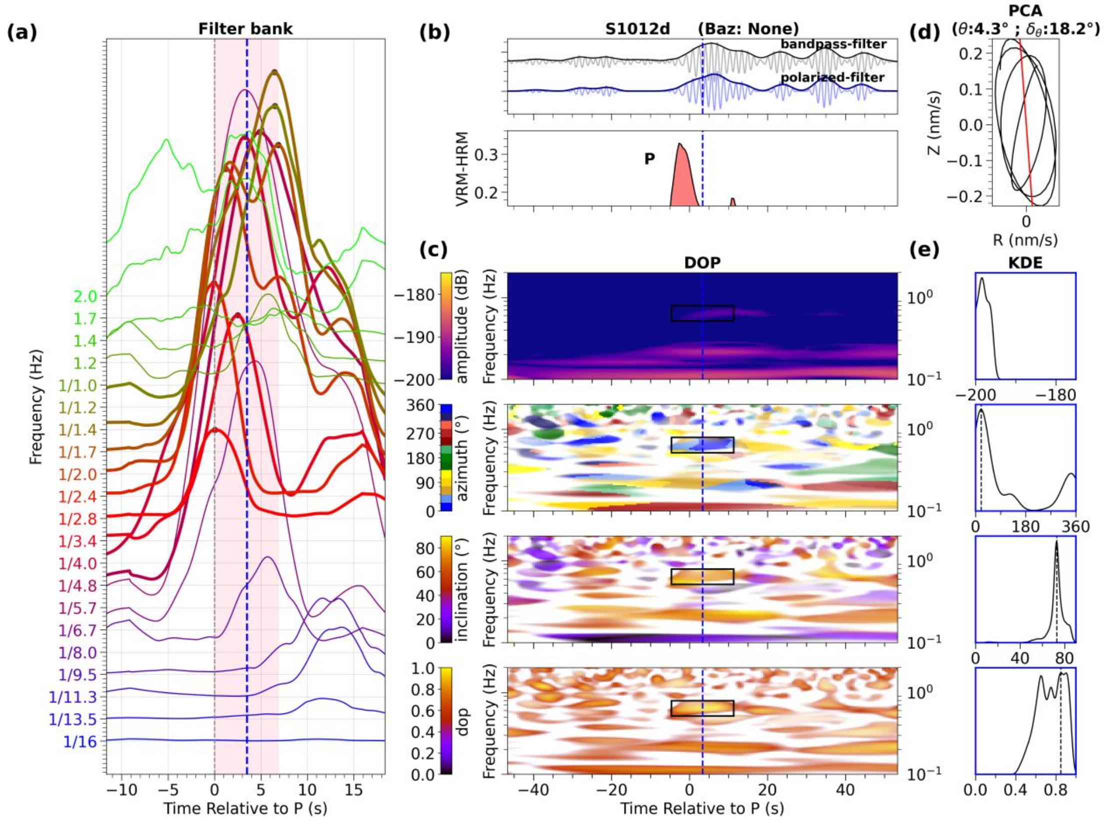

Fig. SS22. Identification of P phase on individual event S1012d, same as Fig. SS1.

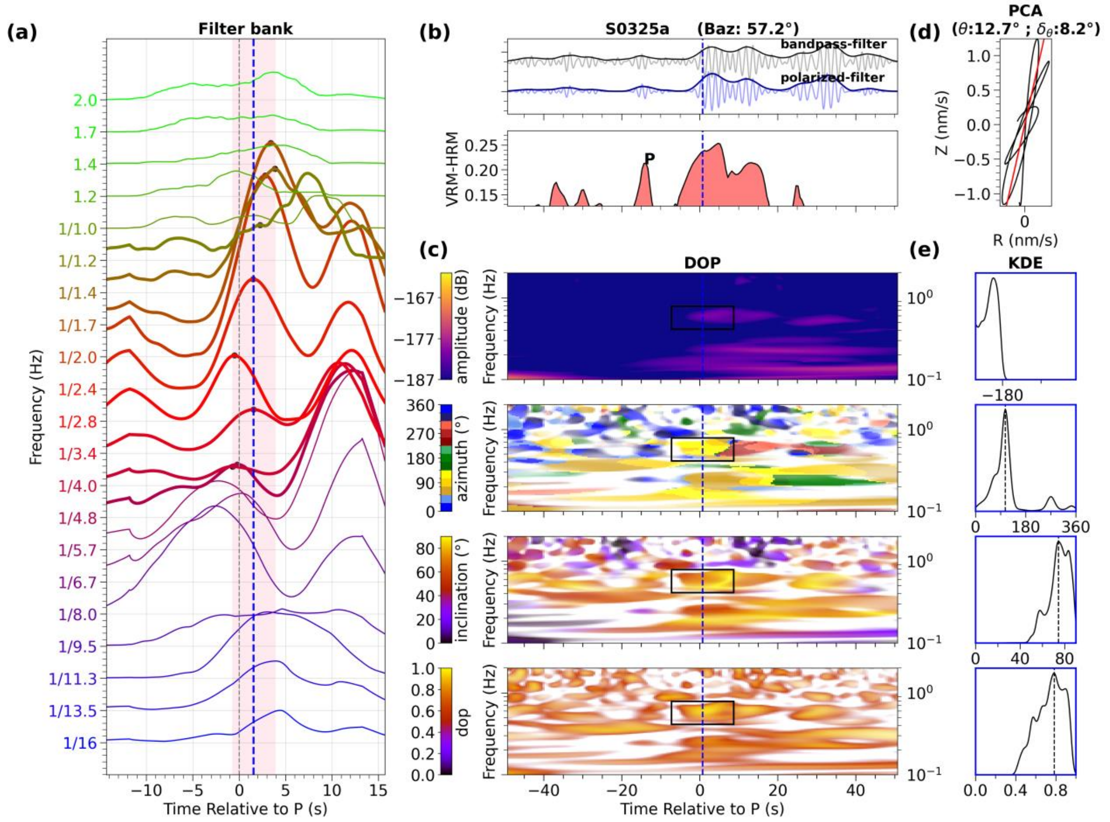

Fig. SS23. Identification of P phase on individual event S0325a, same as Fig. SS1.

2.2 P′P′r_ab

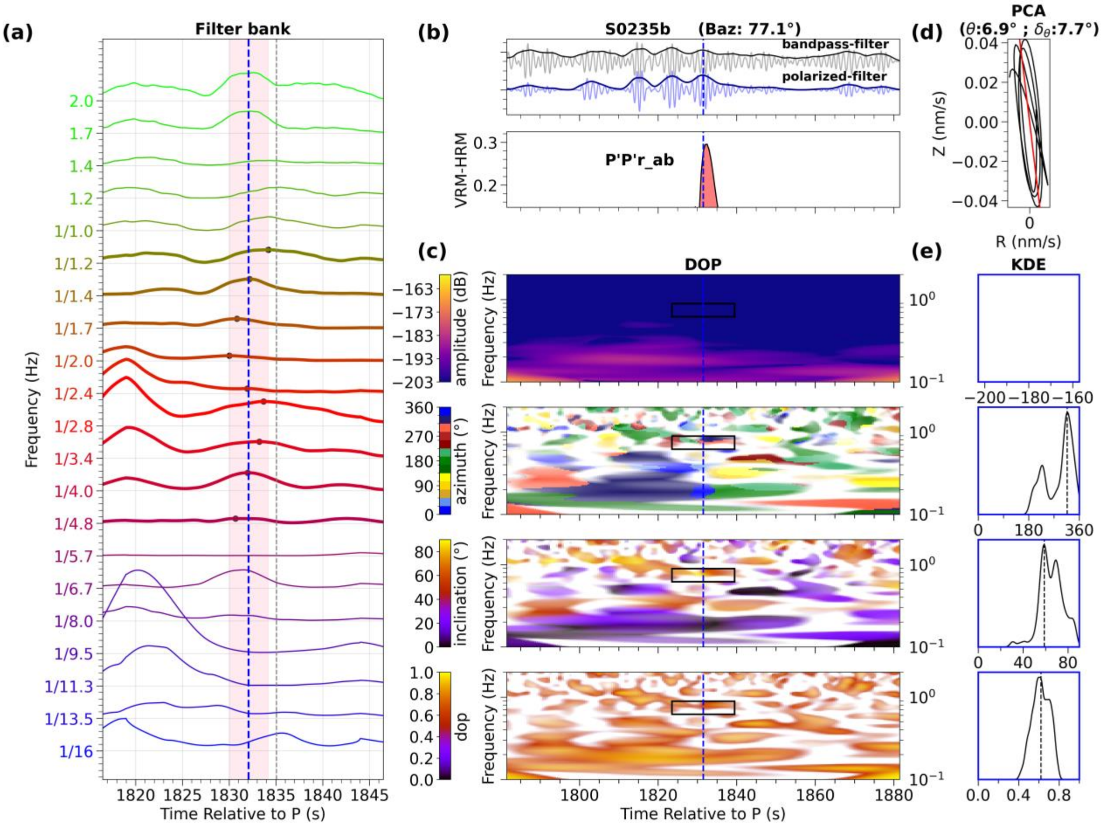

Fig. SS24. Identification of P′P′r_ab phase on individual event S0235b, same as Fig. SS1.

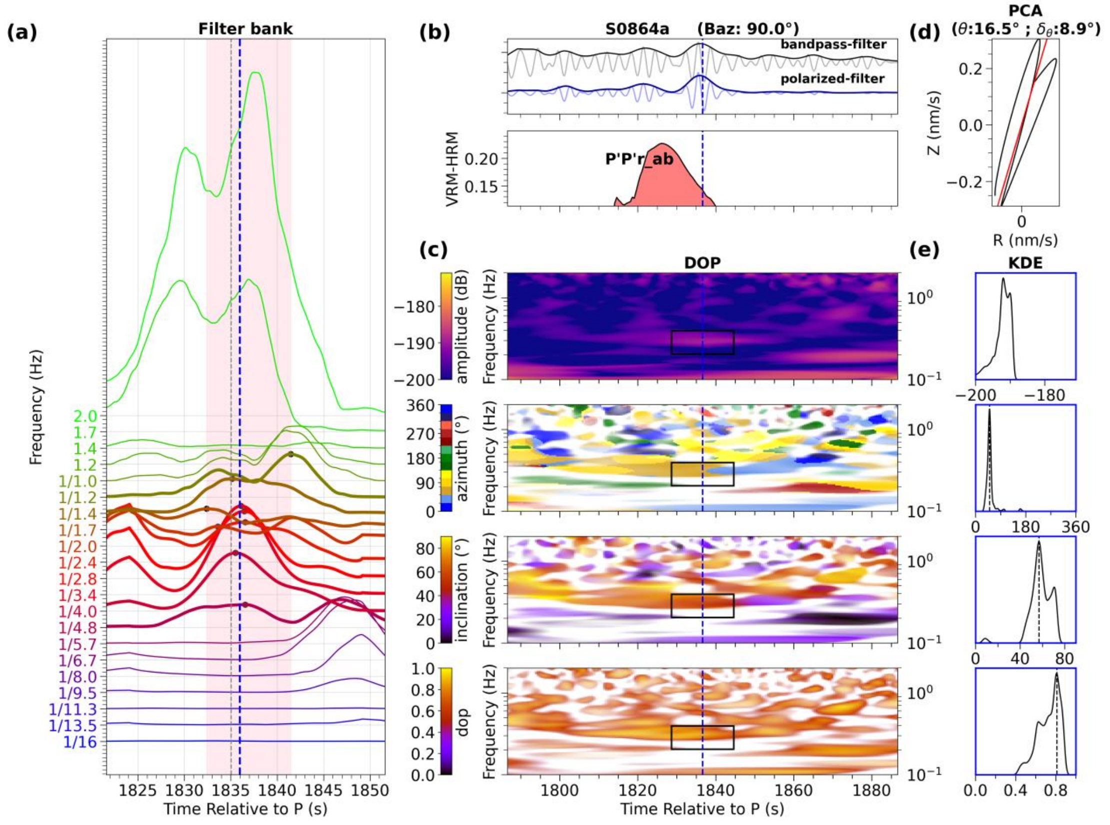

Fig. SS25. Identification of P′P′r_ab phase on individual event S0864a, same as Fig. SS1.

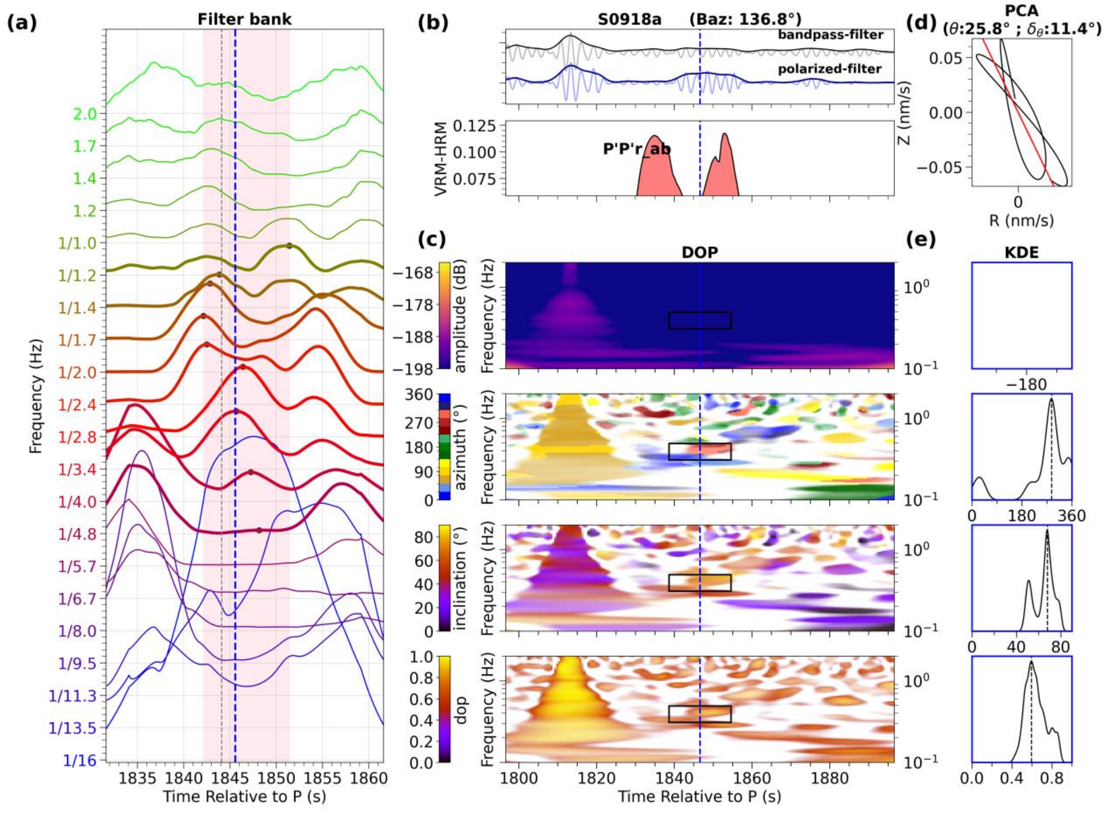

Fig. SS26. Identification of P′P′r_ab phase on individual event S0918a, same as Fig. SS1.

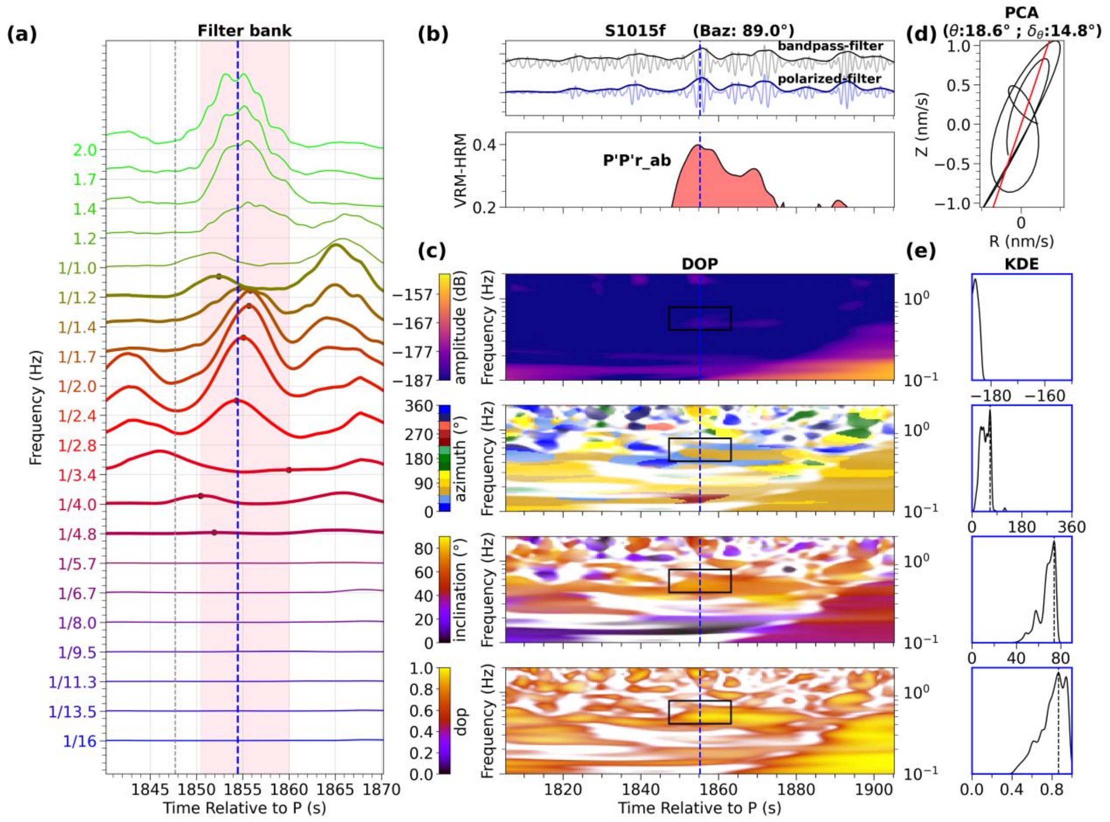

Fig. SS27. Identification of P′P′r_ab phase on individual event S1015f, same as Fig. SS1.

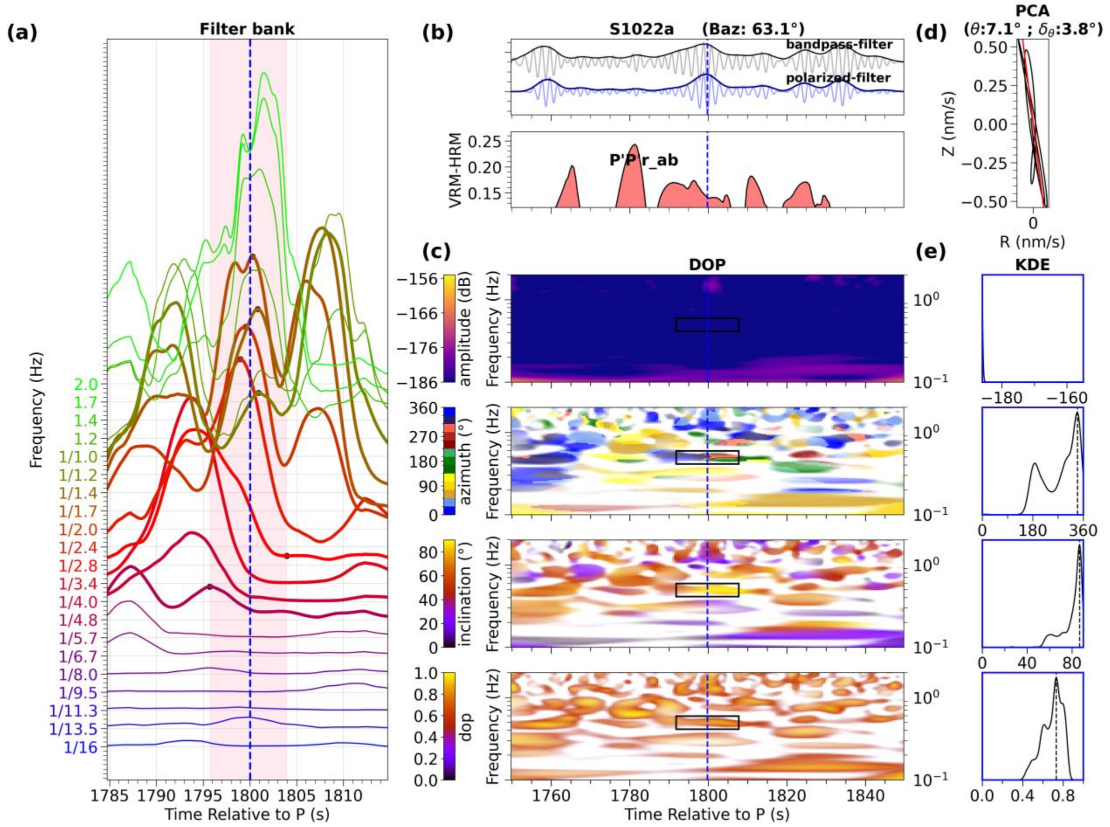

Fig. SS28. Identification of P′P′r_ab phase on individual event S1022a, same as Fig. SS1.

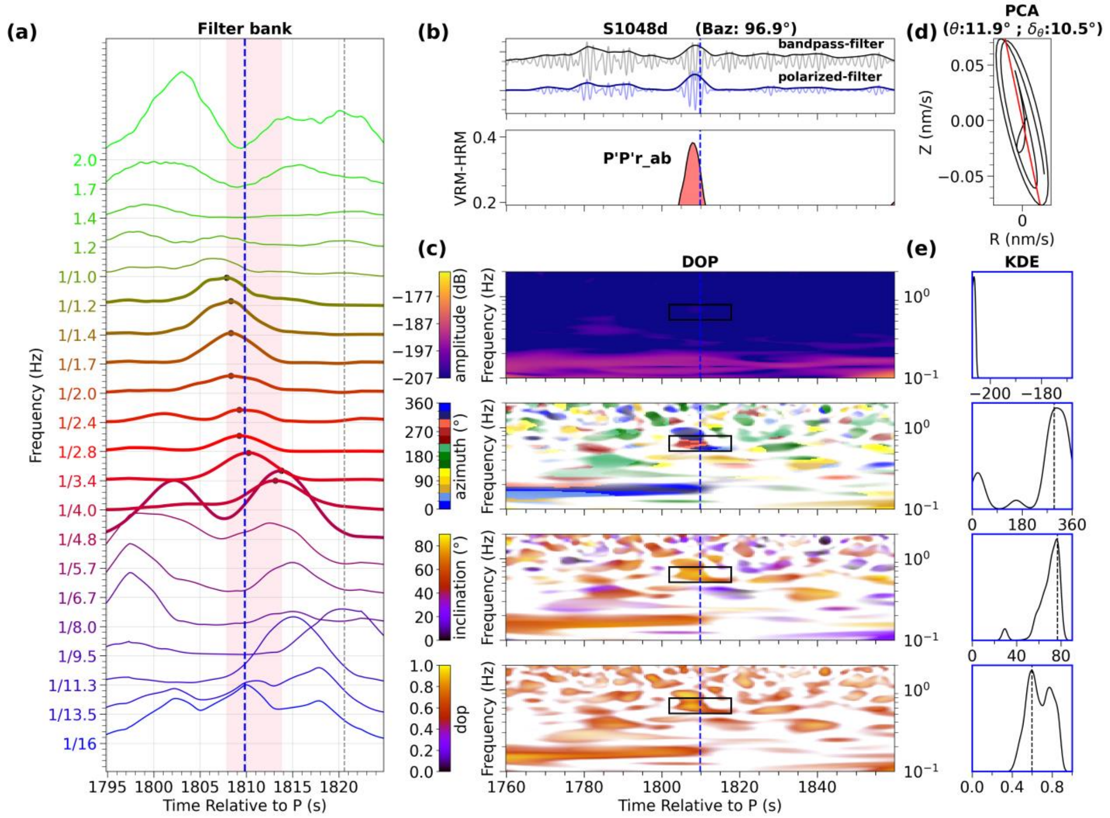

Fig. SS29. Identification of P′P′r_ab phase on individual event S1048d, same as Fig. SS1.

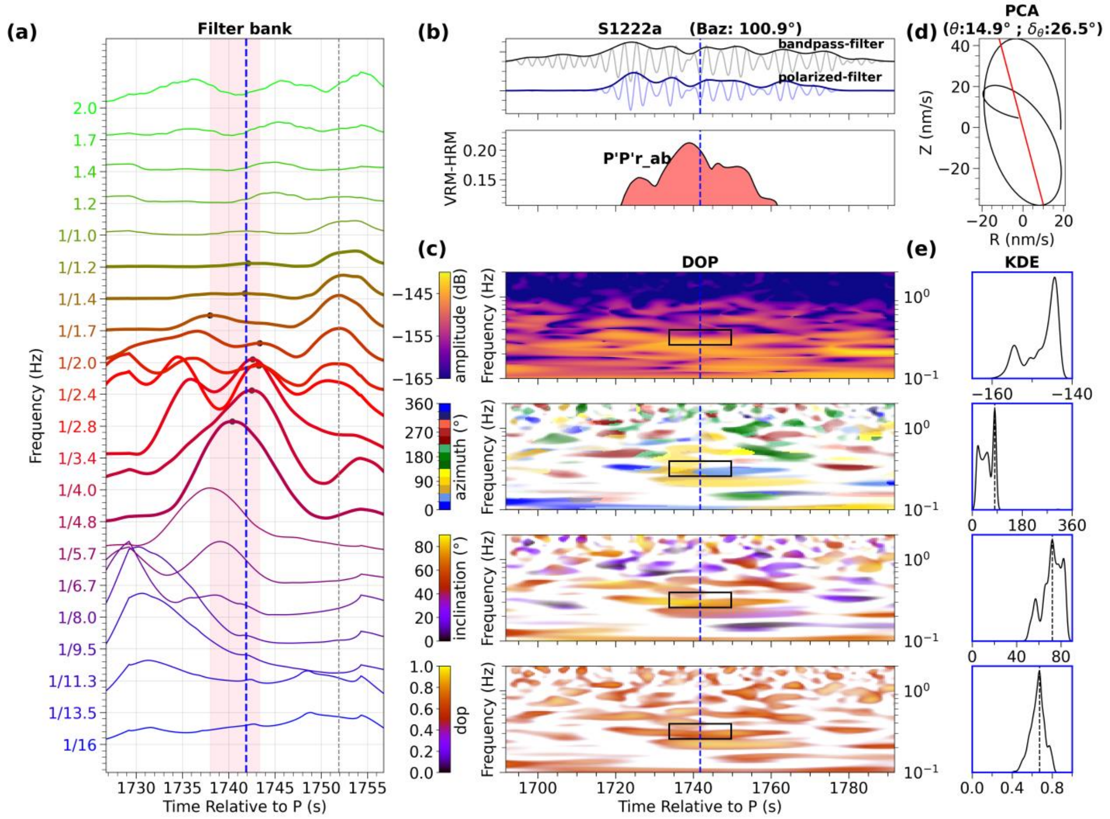

Fig. SS30. Identification of P′P′r_ab phase on individual event S1222a, same as Fig. SS1.

2.3 P′P′n

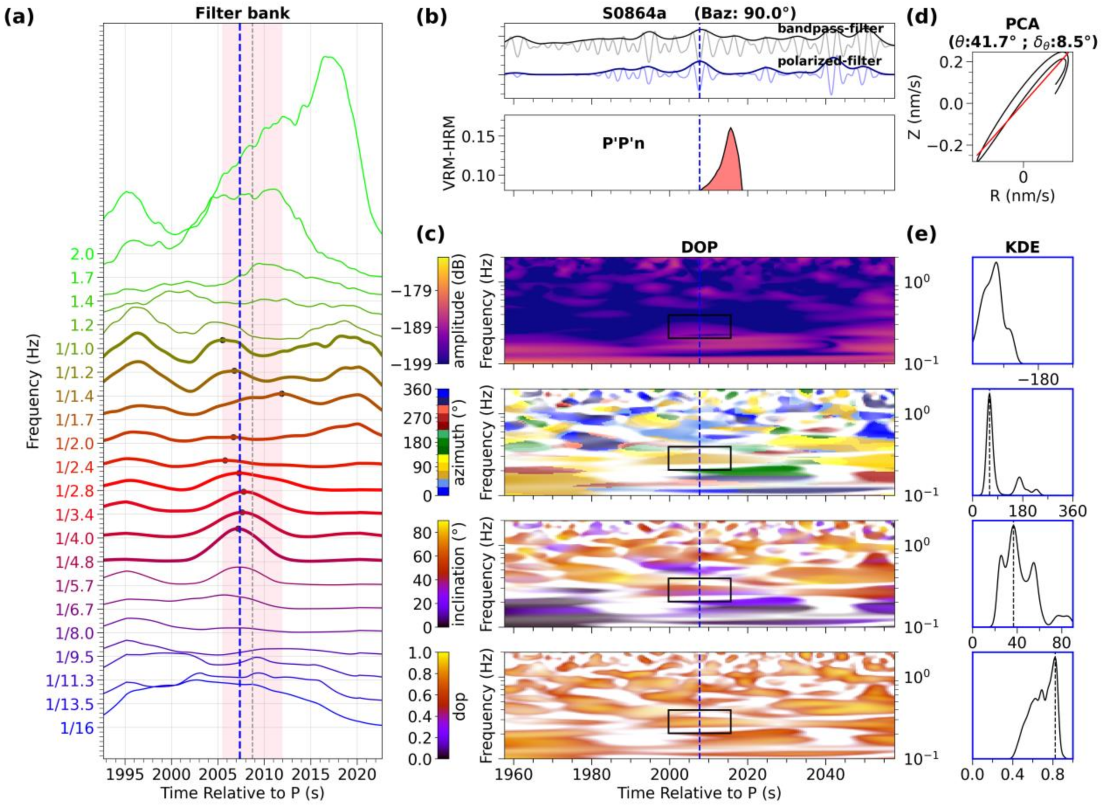

Fig. SS31. Identification of P′P′n phase on individual event S0864a, same as Fig. SS1.

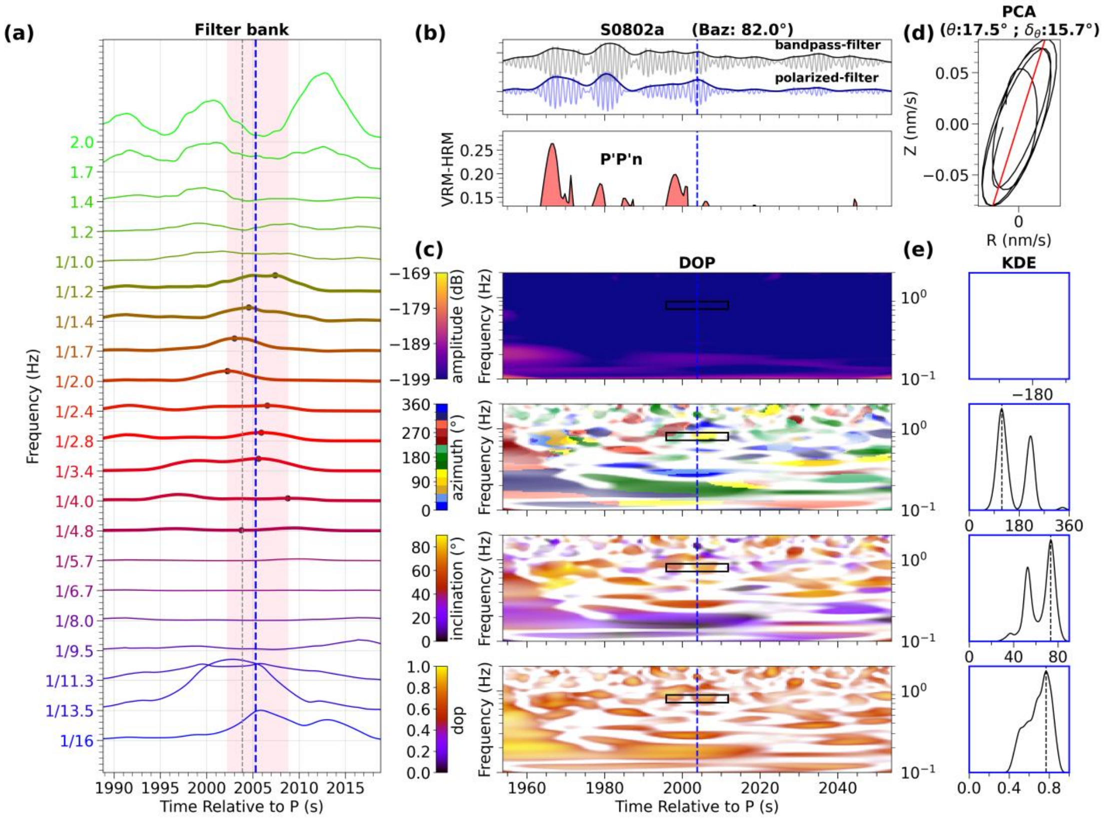

Fig. SS32. Identification of P′P′n phase on individual event S0802a, same as Fig. SS1.

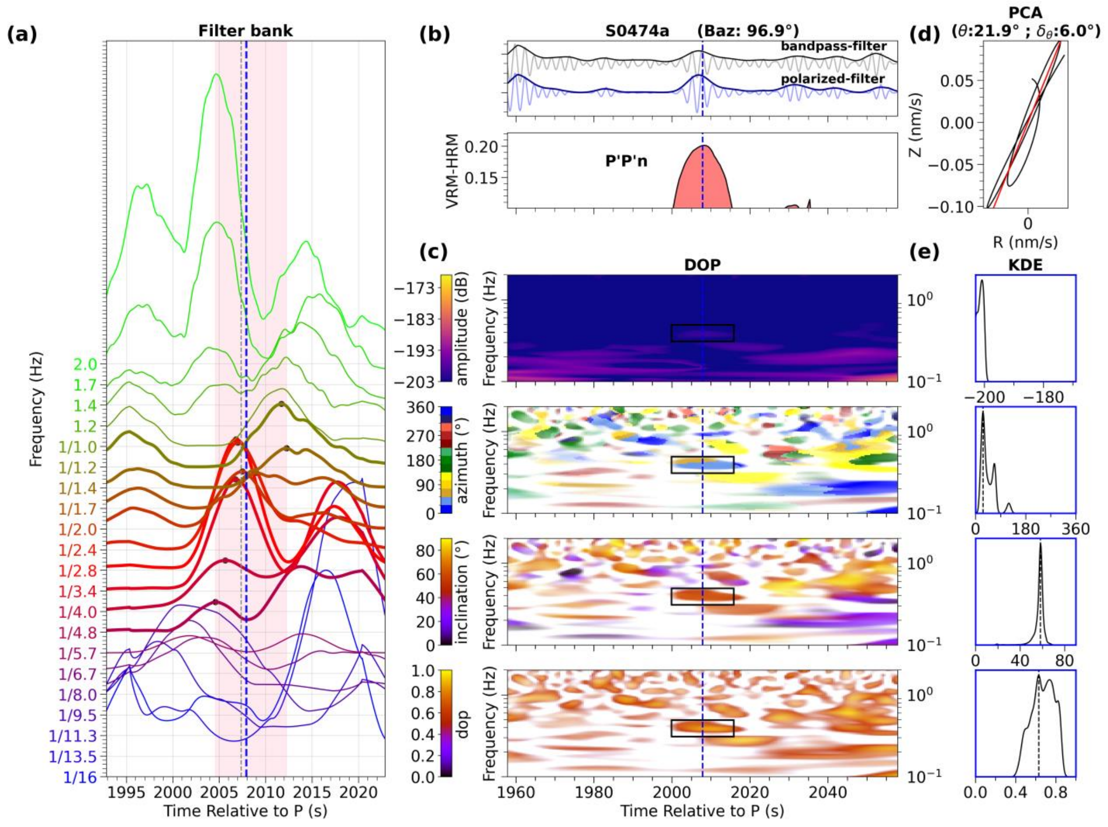

Fig. SS33. Identification of P′P′n phase on individual event S0474a, same as Fig. SS1.

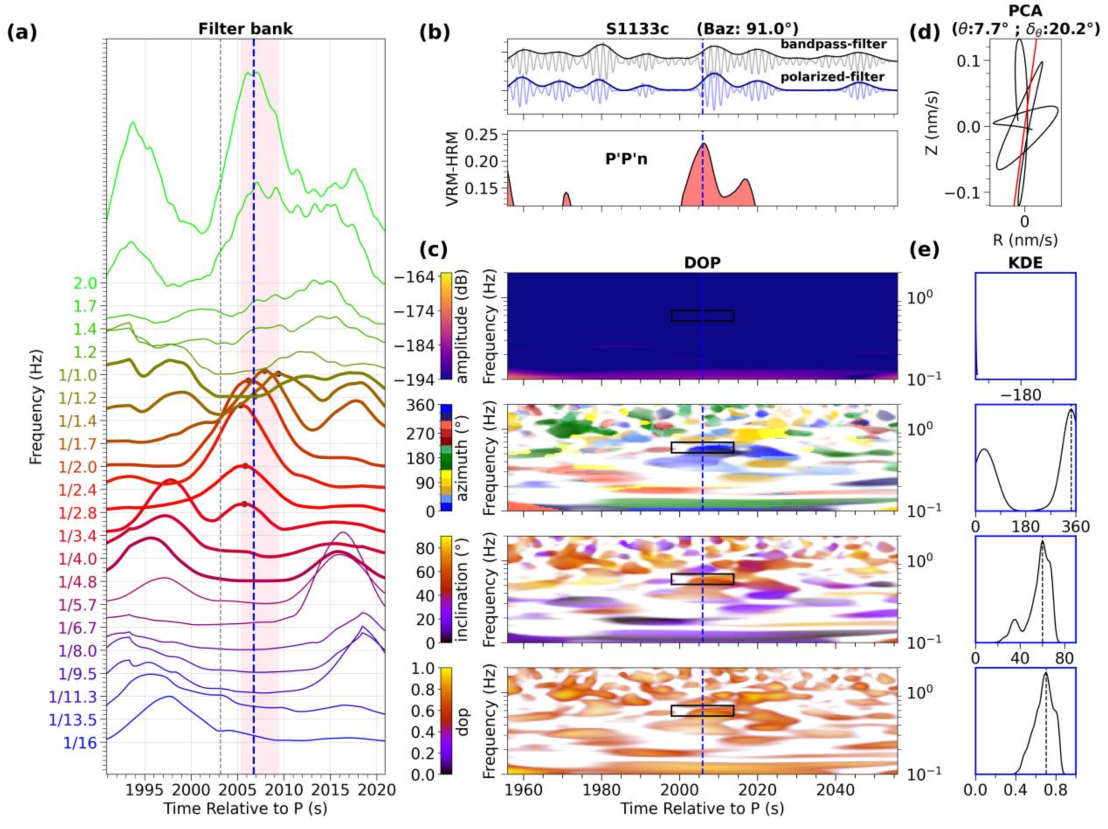

Fig. SS34. Identification of P′P′n phase on individual event S1133c, same as Fig. SS1.

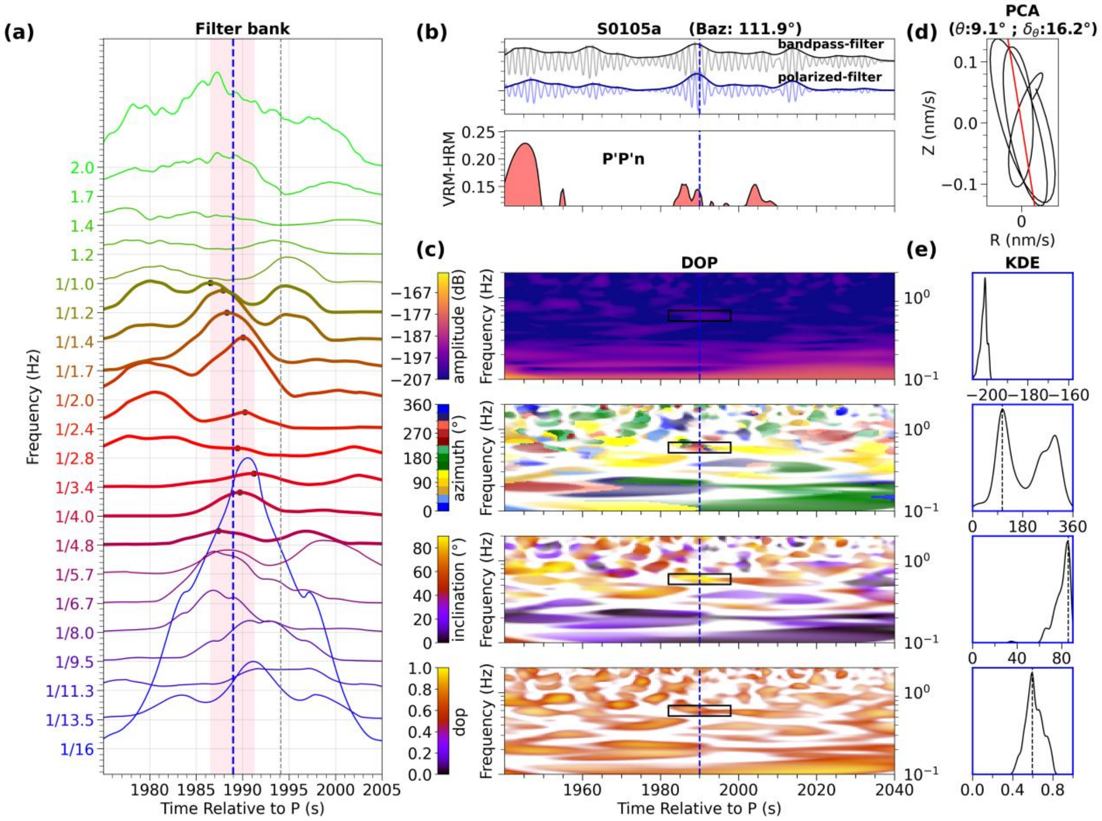

Fig. SS35. Identification of P′P′n phase on individual event S0105a, same as Fig. SS1.

2.4 PKKP

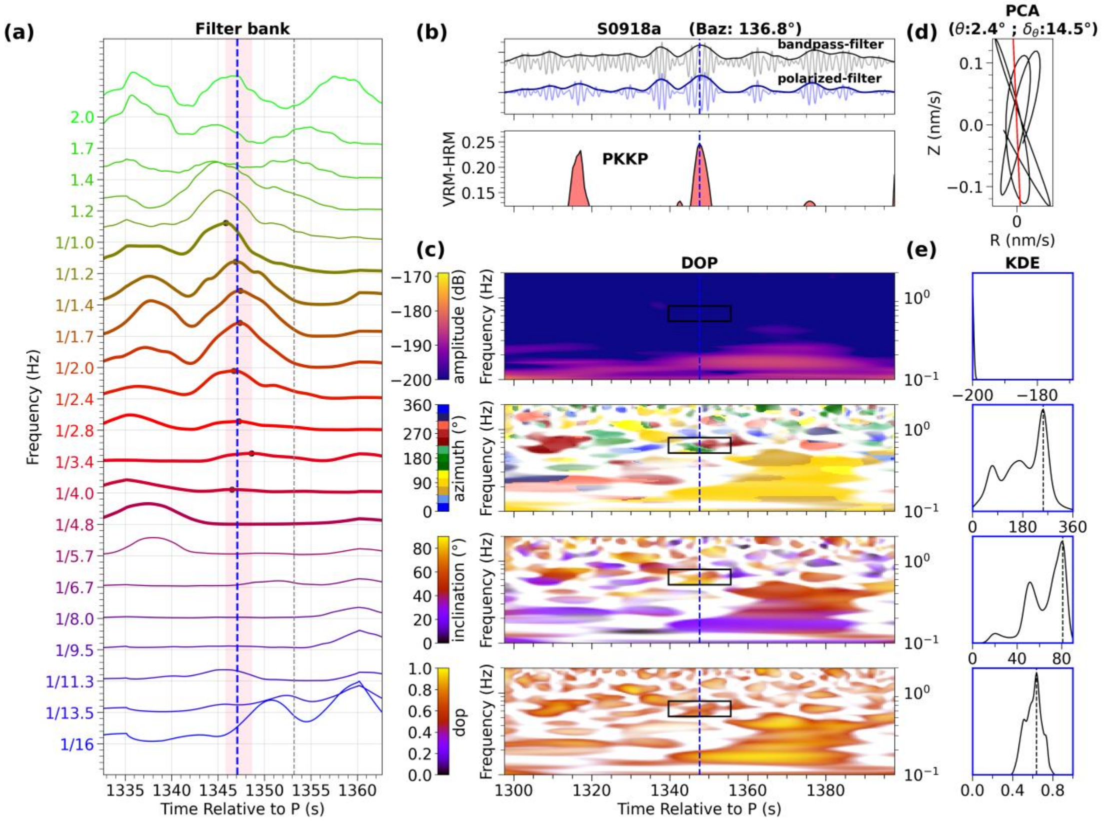

Fig. SS36. Identification of PKKP phase on individual event S0918a, same as Fig. SS1.

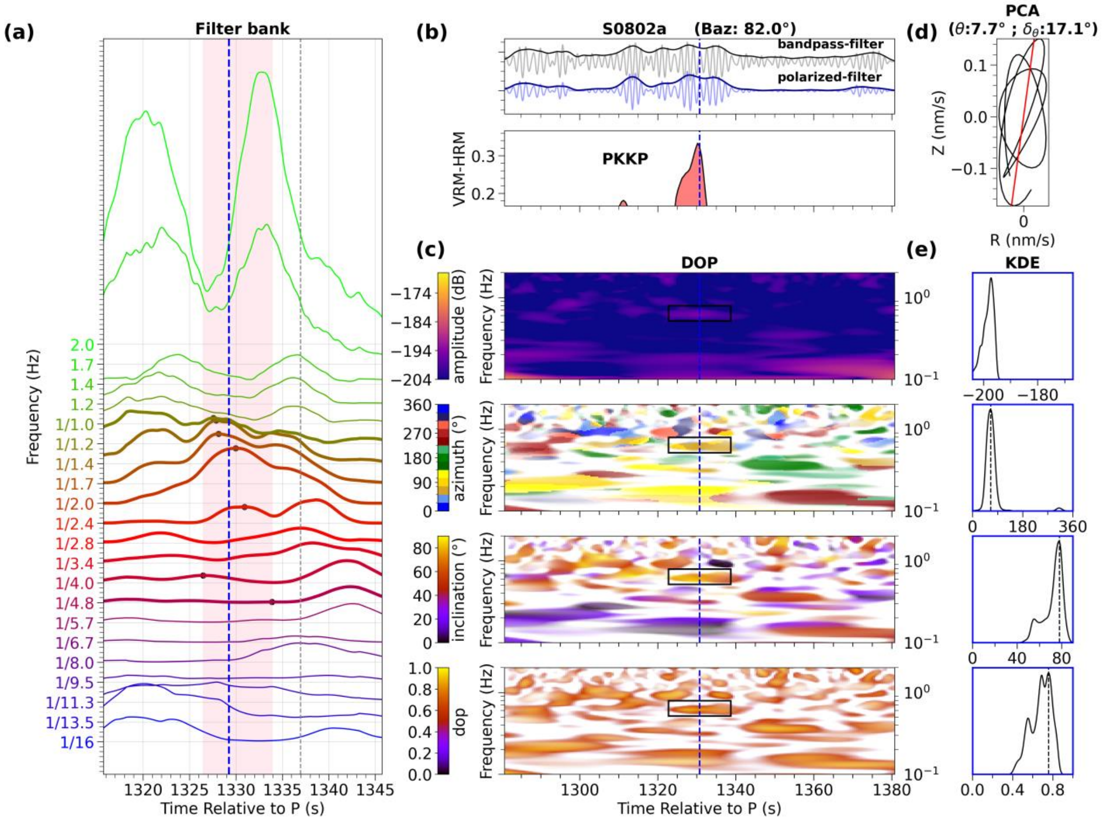

Fig. SS37. Identification of PKKP phase on individual event S0802a, same as Fig. SS1.

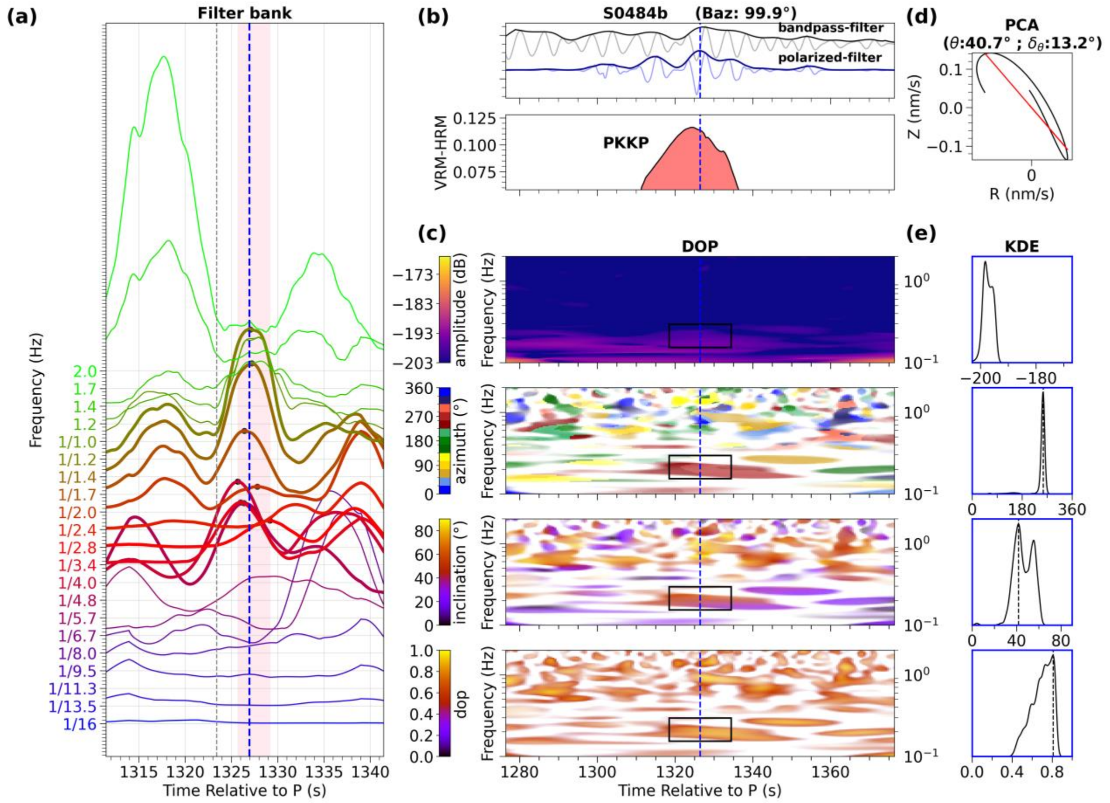

Fig. SS38. Identification of PKKP phase on individual event S0484b, same as Fig. SS1.

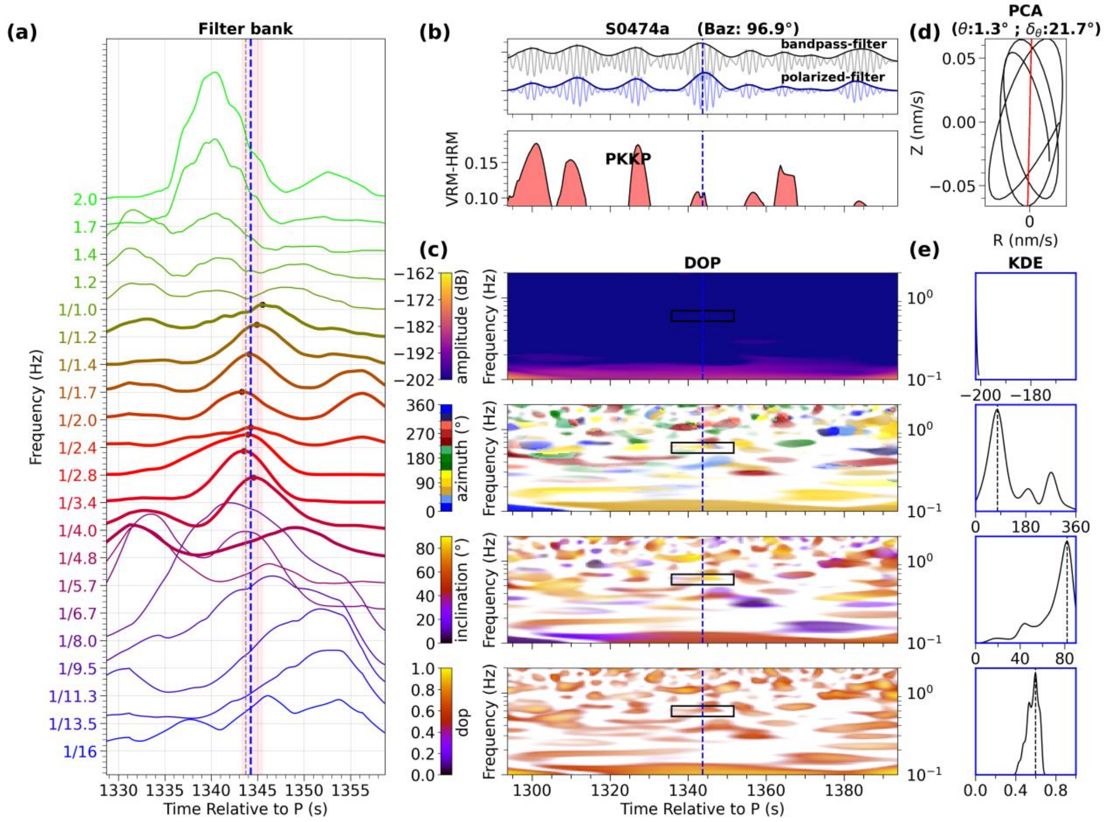

Fig. SS39. Identification of PKKP phase on individual event S0474a, same as Fig. SS1.

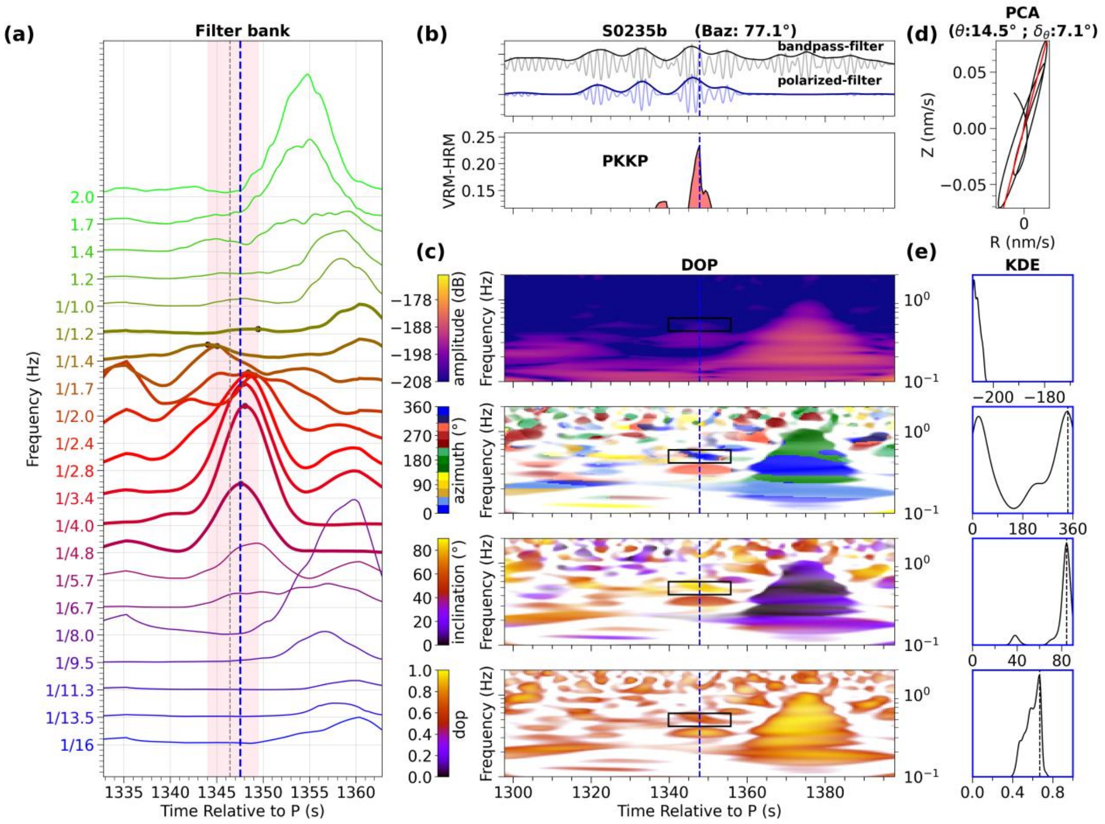

Fig. SS40. Identification of PKKP phase on individual event S0235b, same as Fig. SS1.

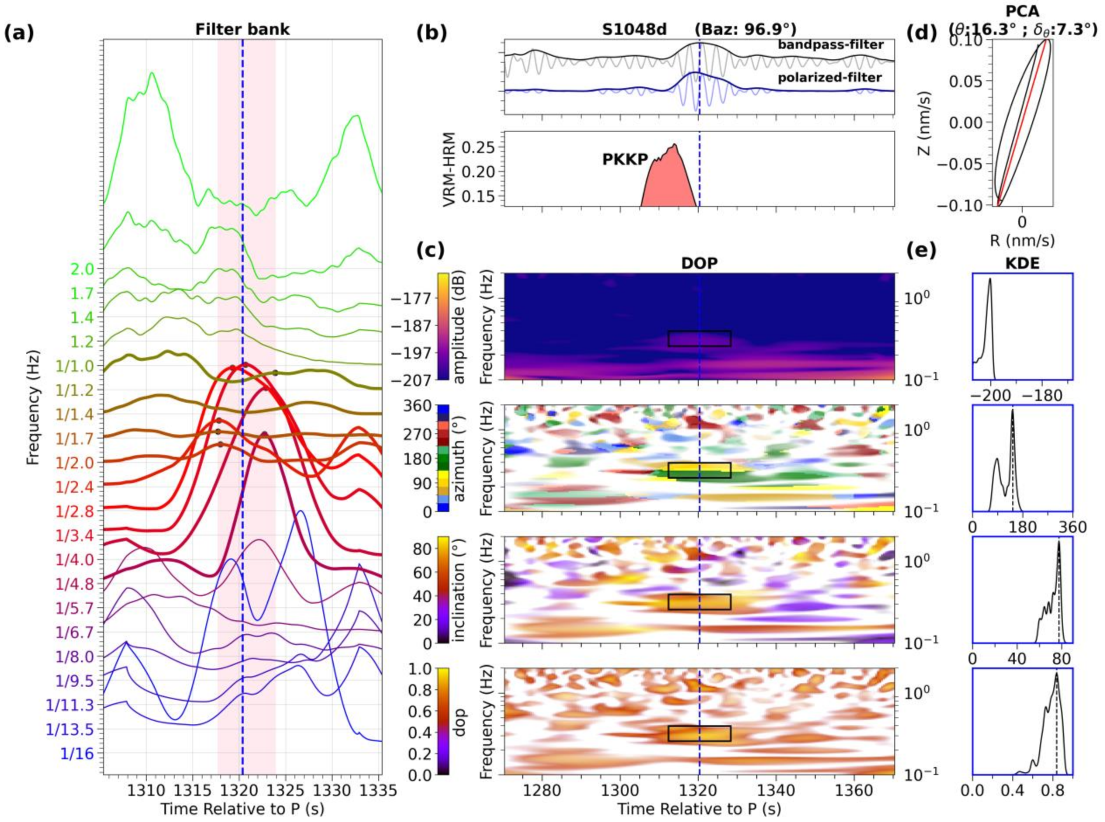

Fig. SS41. Identification of PKKP phase on individual event S1048d, same as Fig. SS1.

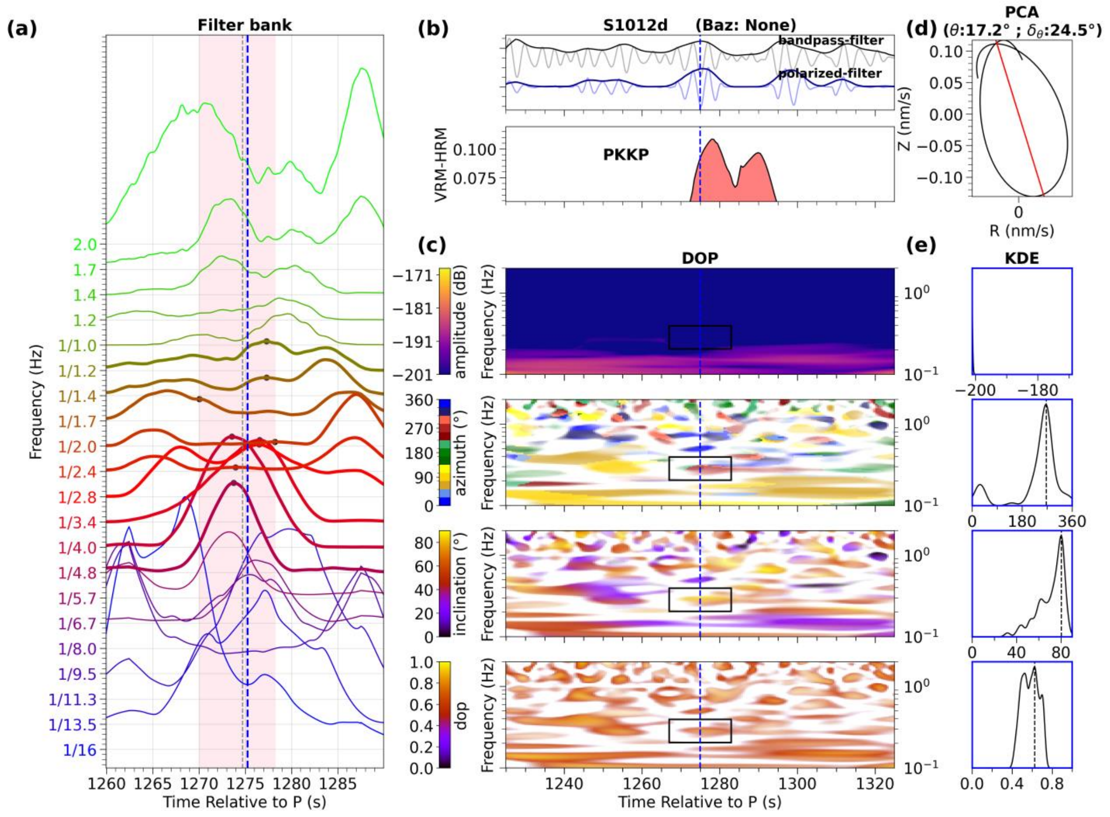

Fig. SS42. Identification of PKKP phase on individual event S1012d, same as Fig. SS1.

2.5 PKiKP

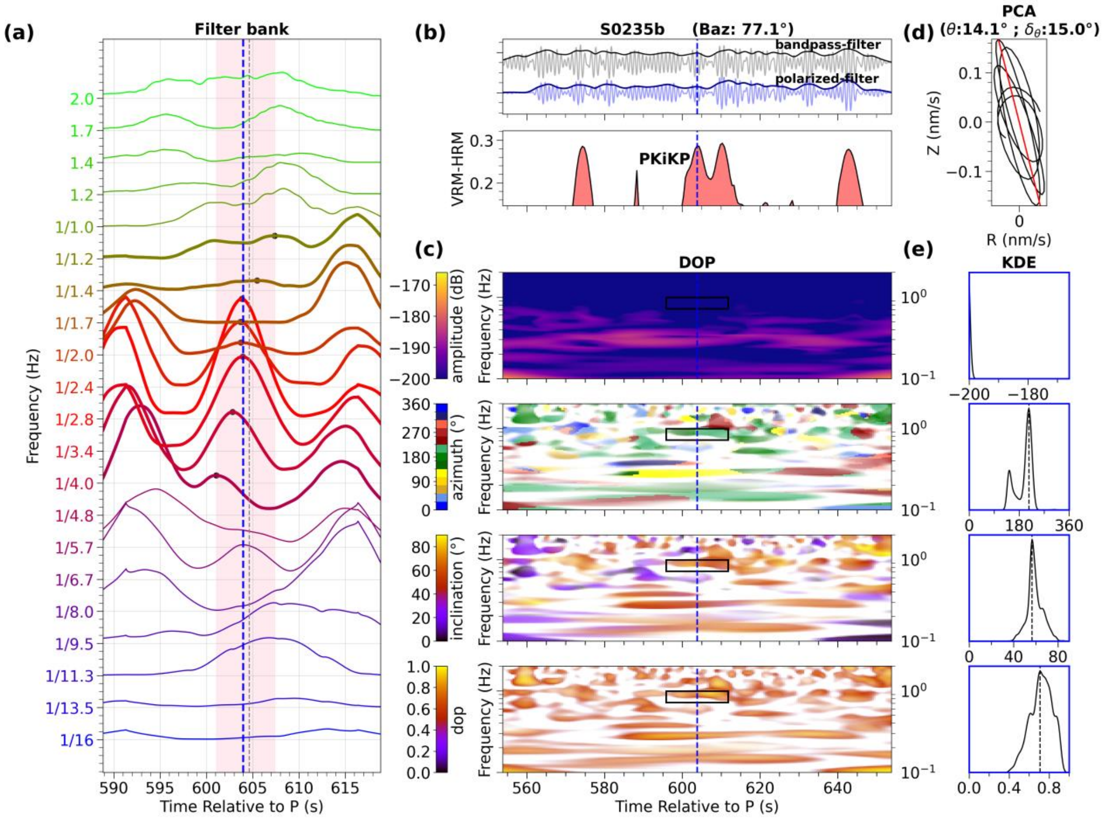

Fig. SS43. Identification of the PKiKP phase for event S0235b.

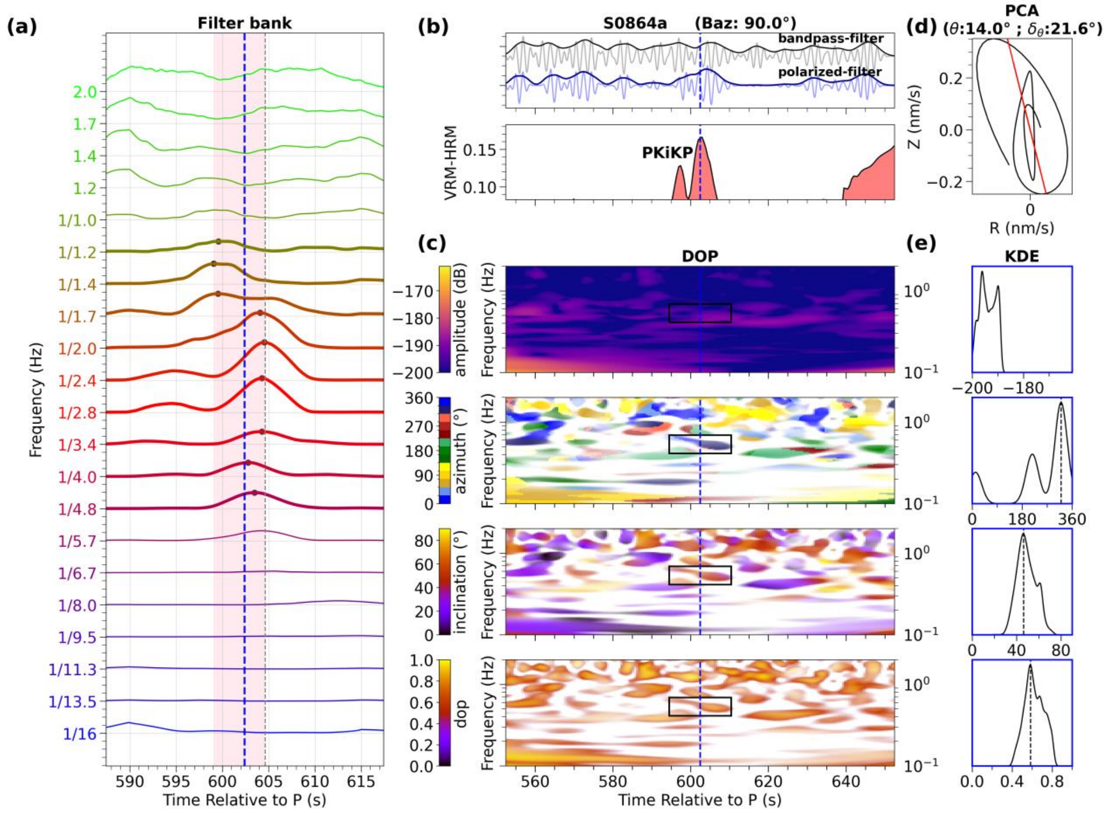

Fig. SS44. Identification of the PKiKP phase for event S0864a.

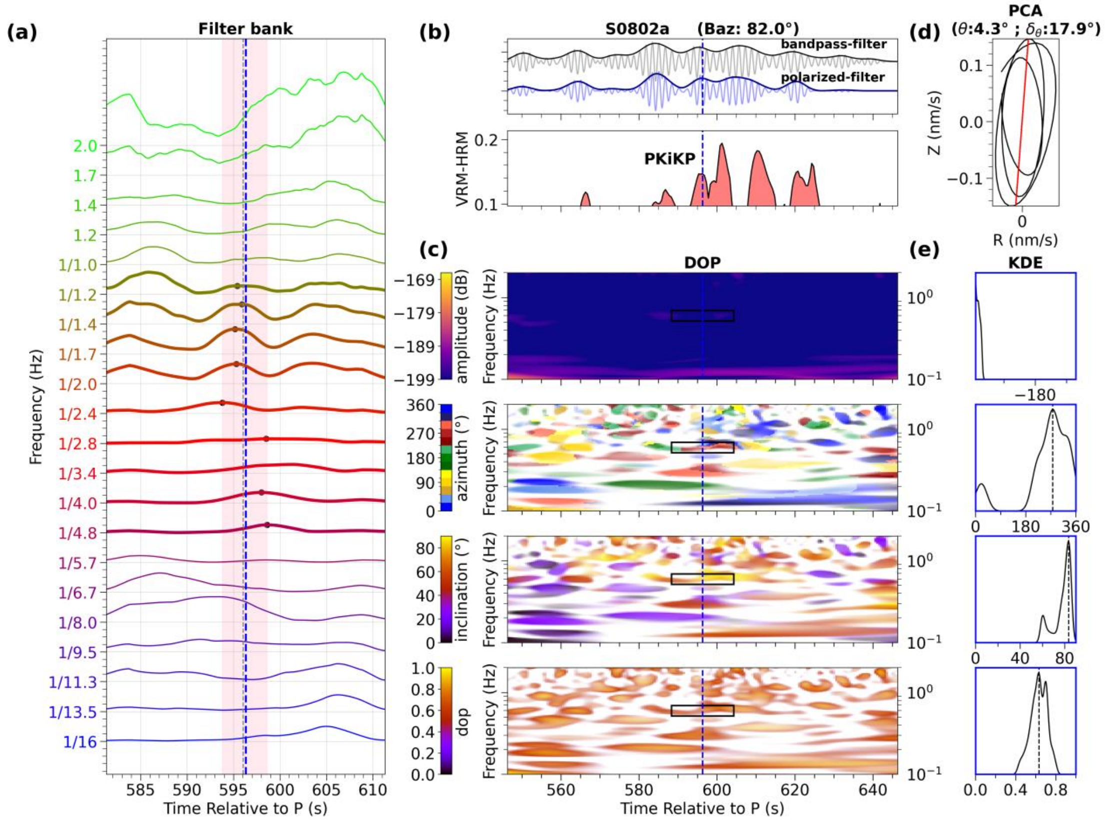

Fig. SS45. Identification of the PKiKP phase for event S0802a.

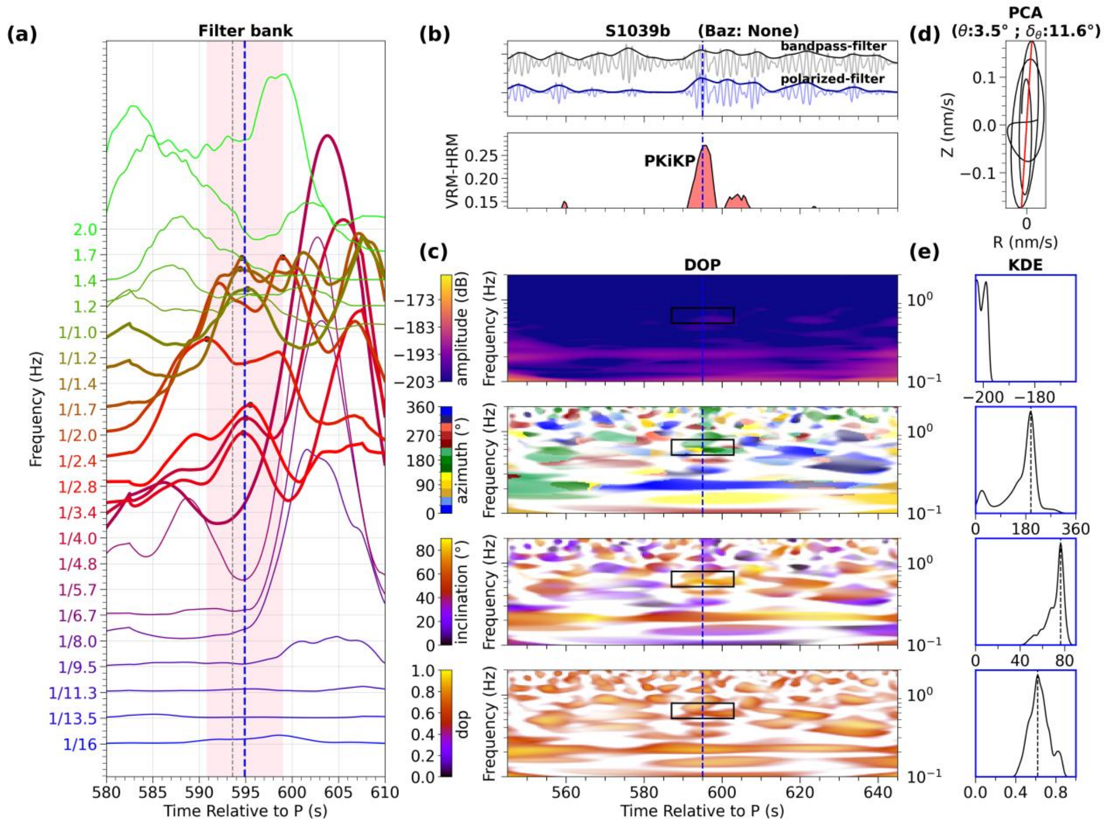

Fig. SS46. Identification of the PKiKP phase for event S1039b.

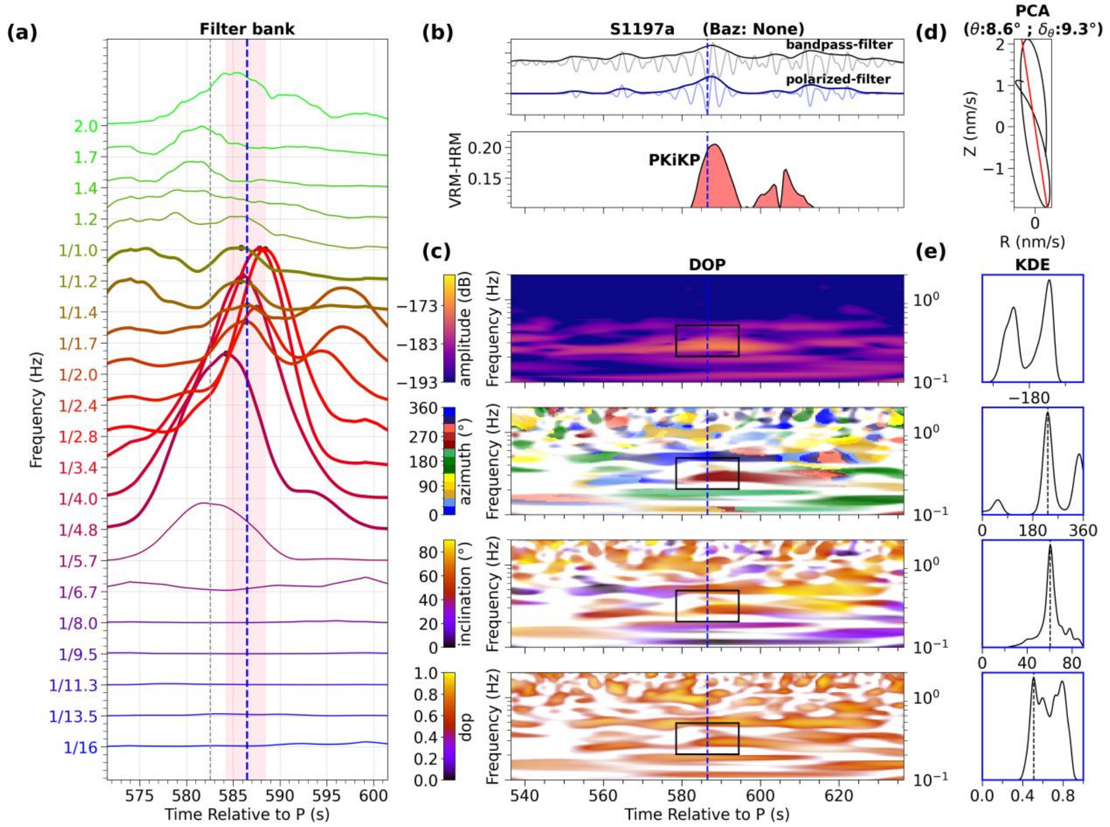

Fig. SS47. Identification of PKiKP phase on individual event S1197a.

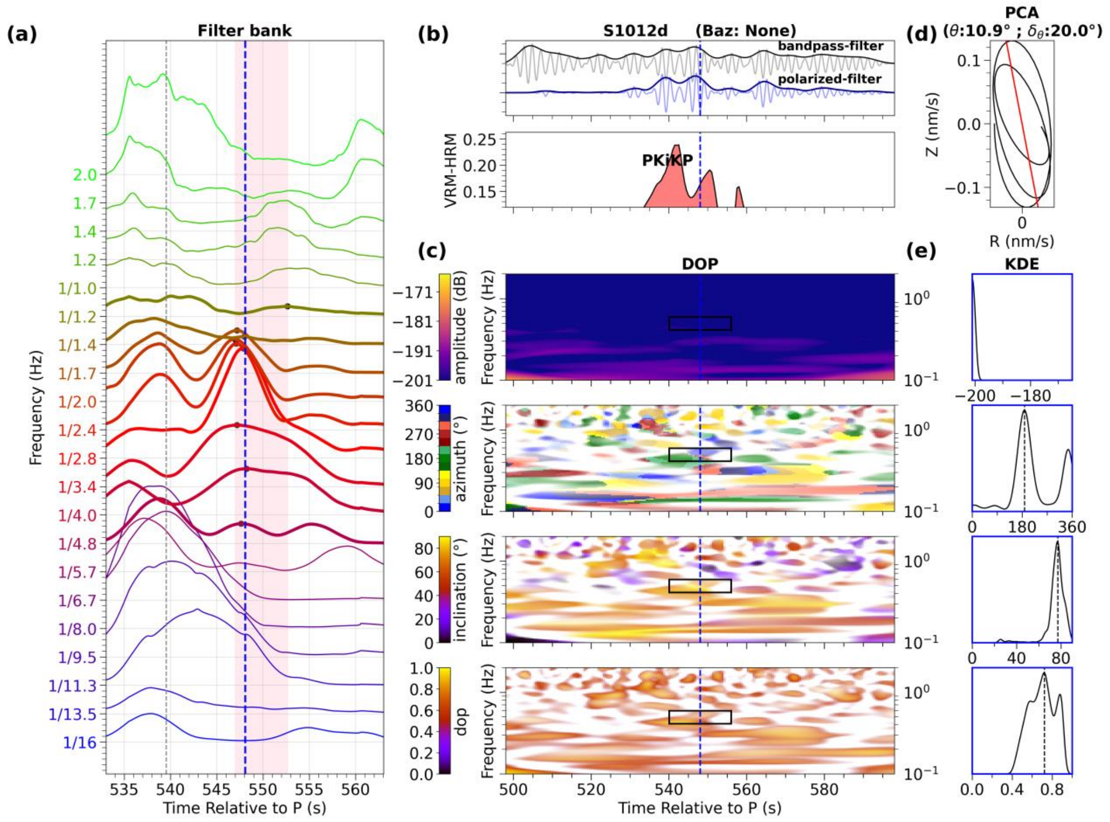

Fig. SS48. Identification of the PKiKP phase for event S1012d.

3 Inversion results from M_pick method

In this section, we display the inversion results obtained from M_pick method.

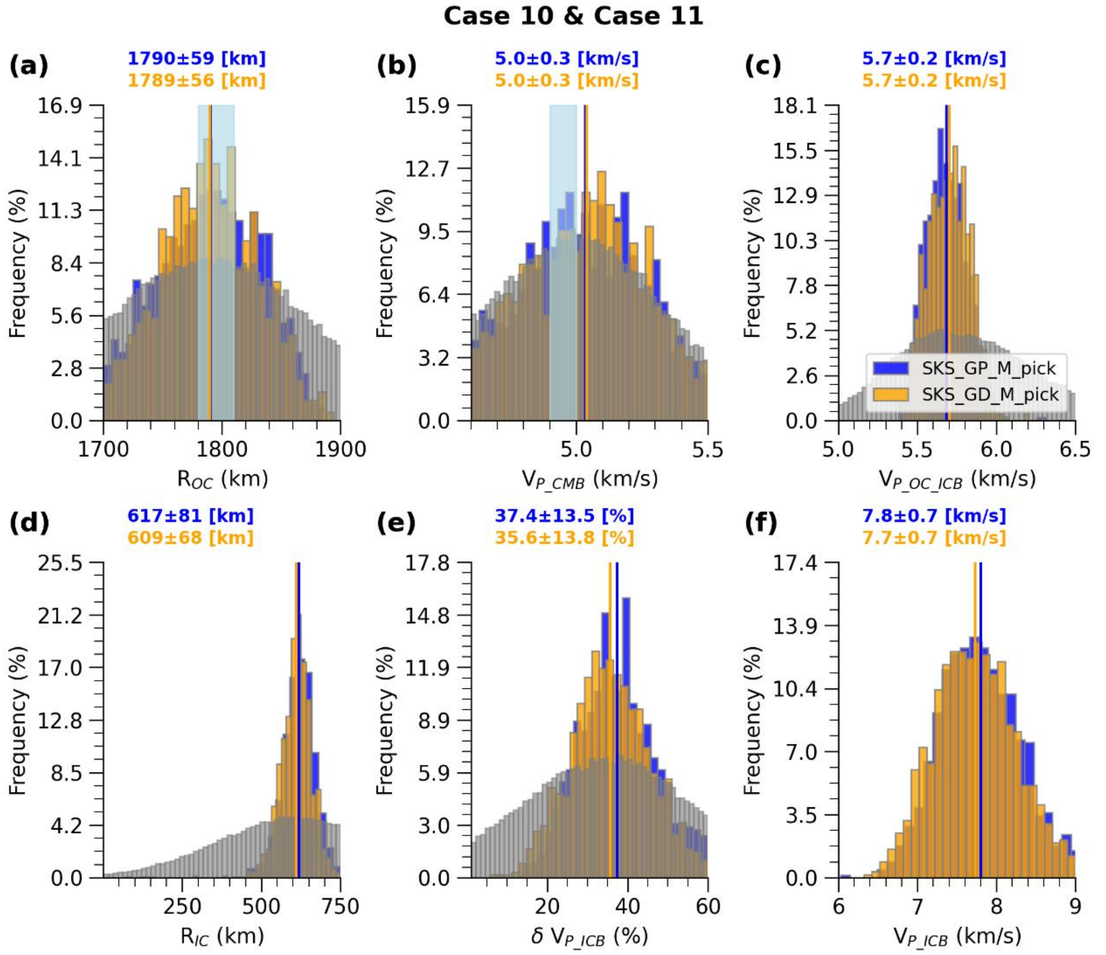

Fig. SS49. Marginal distributions of inverted core parameters using the M_pick method. (a) Outer-core radius (R_{OC}), (b) P velocity at the CMB (V_{P_CMB}), P velocity of the outer-core side at the ICB ($V_{P\_OC\_ICB}$), (d) inner-core radius (R_{IC}), (e) P velocity jump at the ICB (δV_{P_ICB}), and (f) P velocity of the IC side at the ICB (V_{P_ICB}). Note that V_{P_ICB} is not an inverted parameter and is directly calculated from $V_{P\_OC\_ICB}$ and δV_{P_ICB}. The blue and orange histograms correspond to the posteriori distributions of two different mantle velocity models, namely SKS_GP and SKS_GD, with their mean values depicted by solid lines of corresponding colors. Mean values and 85% confidence intervals are indicated with colored text at the top. The gray histograms represent the priori distribution using the SKS_GD mantle velocity model.

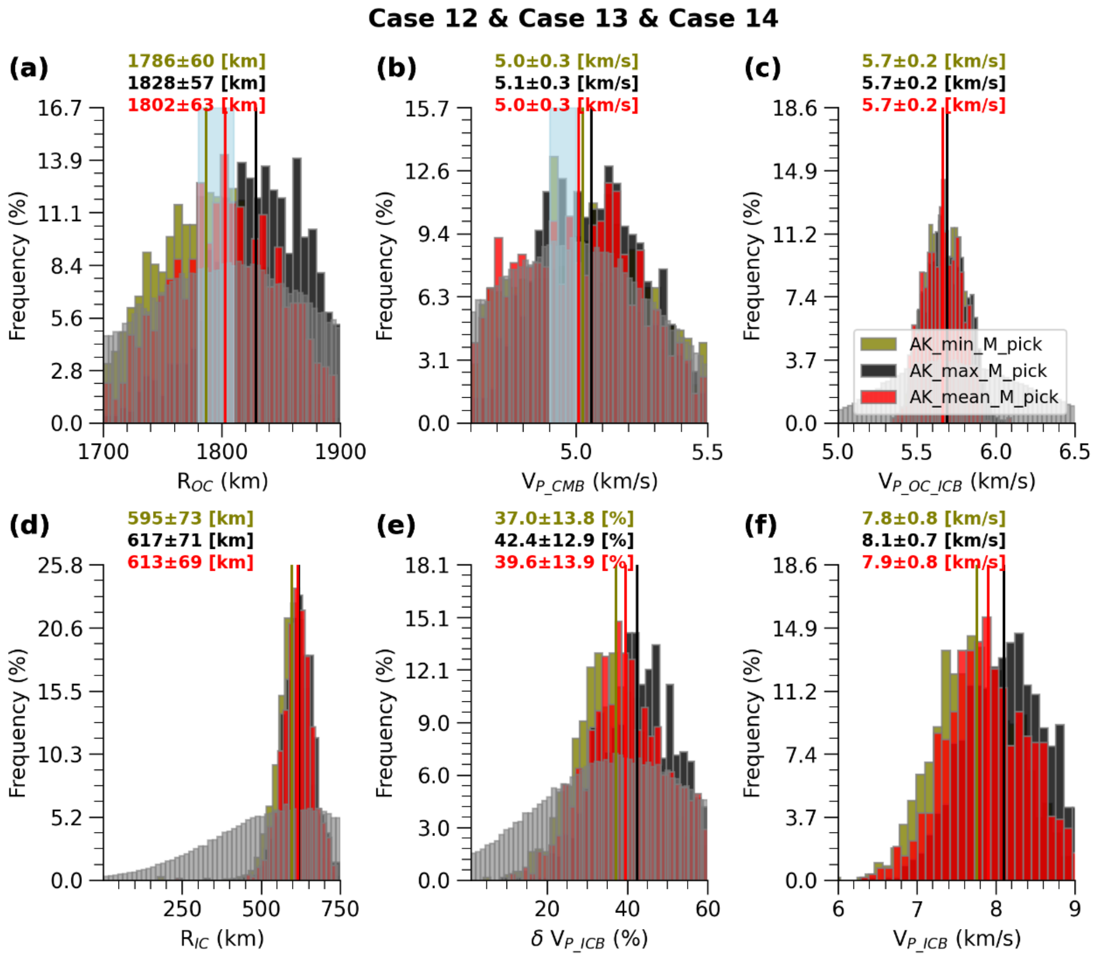

Fig. SS50. Marginal distributions of inverted core parameters based on AK_min (olive), AK_max (black), and AK_mean (red) models. The gray histograms represent the priori distribution using the AK_mean model.

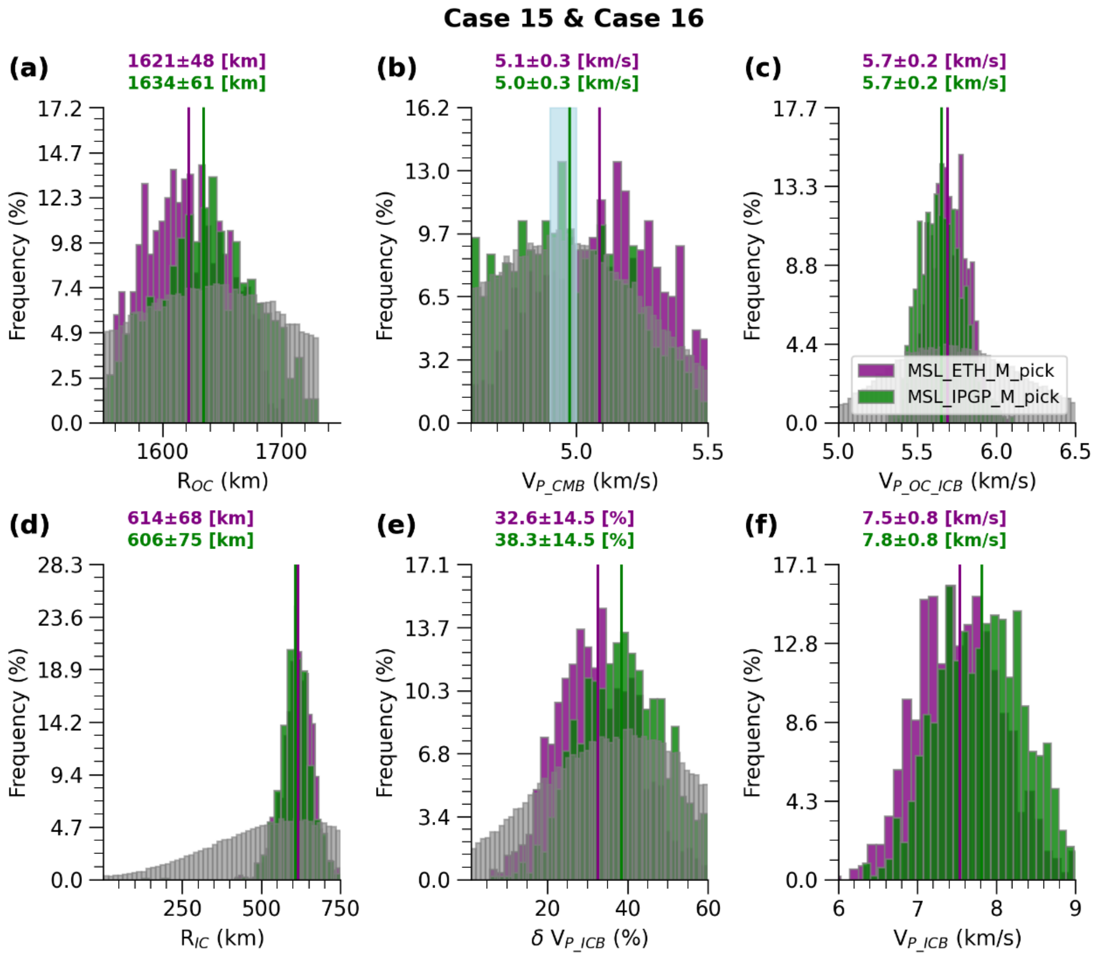

Fig. SS51. Marginal distributions of inverted core parameters based on MSL_IPGP (green) and MSL_ETH (purple) model. The gray histograms represent the priori distribution using the MSL_ETH model.

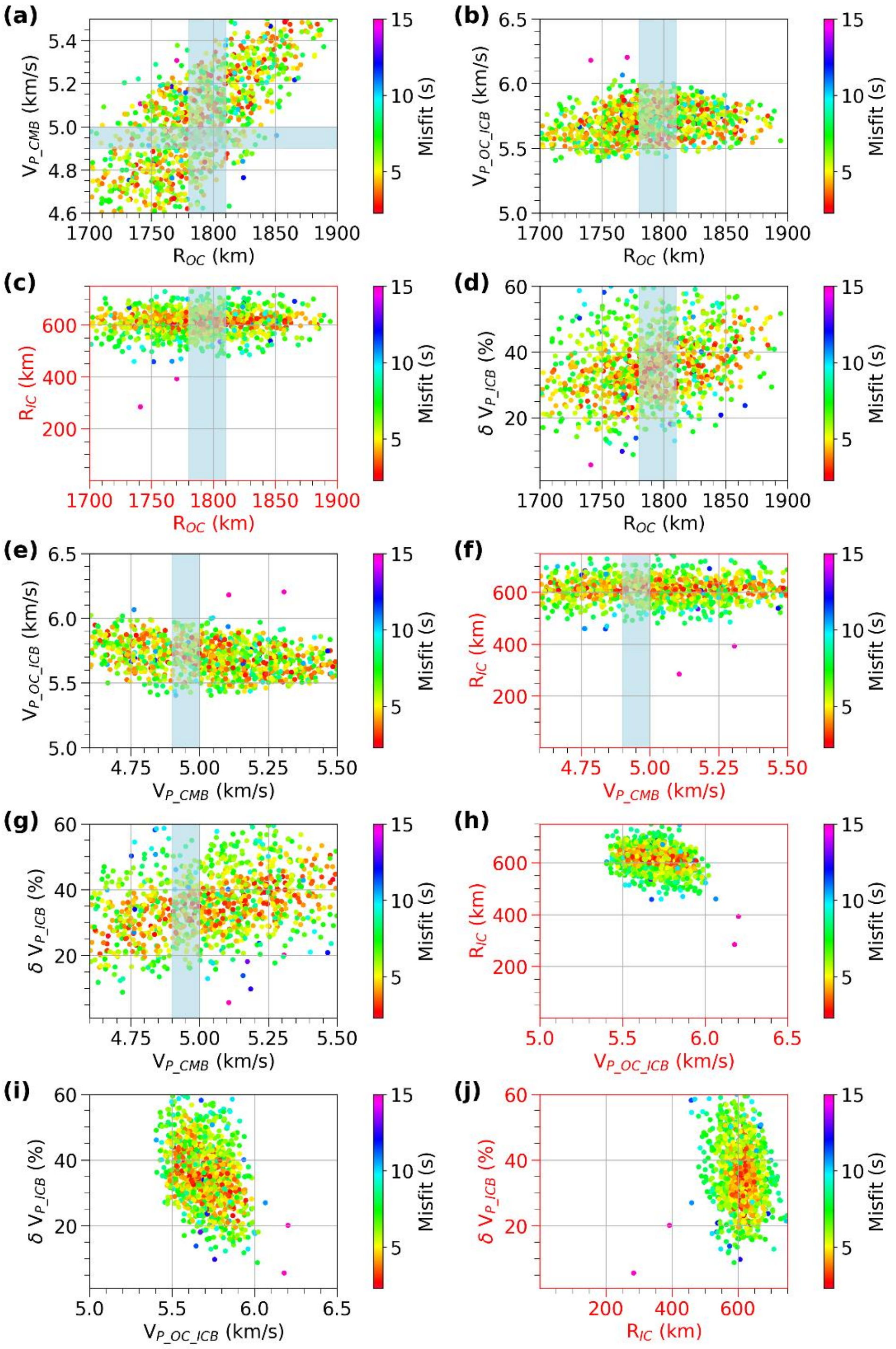

Fig. SS52. Trade-offs among the five inverted core parameters based on the SKS_GD model. The four subfigures (c, f, h, and i) with red borders highlight the trade-offs between the inner-core radius (R_{IC}) and the other four parameters.

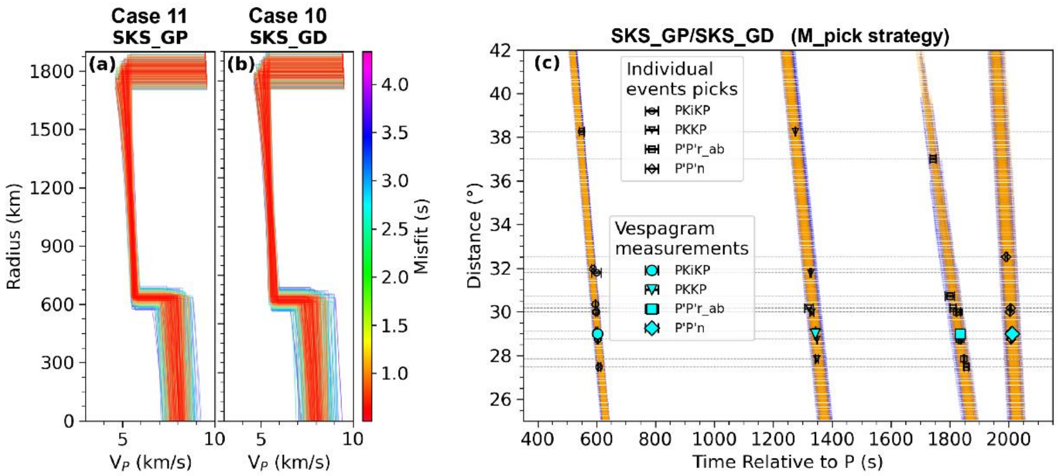

Fig. SS53. P velocity profiles of the Martian core based on the inverted parameters for two mantle models of (a) SKS_GP and (b) SKS_GD. The plots display results using the M_pick strategy, in which only four travel times (cyan solid symbols in (c)) from the vespagram measurements are used for the inversion here. The colored lines in (a)-(b) represent differential travel time misfits between the data and predictions from the inverted models. (c) Predicted differential travel times for all models in (a)-(b). Blue and orange lines denote the predictions from the inverted core models based on the SKS_GP and SKS_GD models, respectively. The open and cyan solid symbols denote the individual events picks and vespagram measurements, respectively.

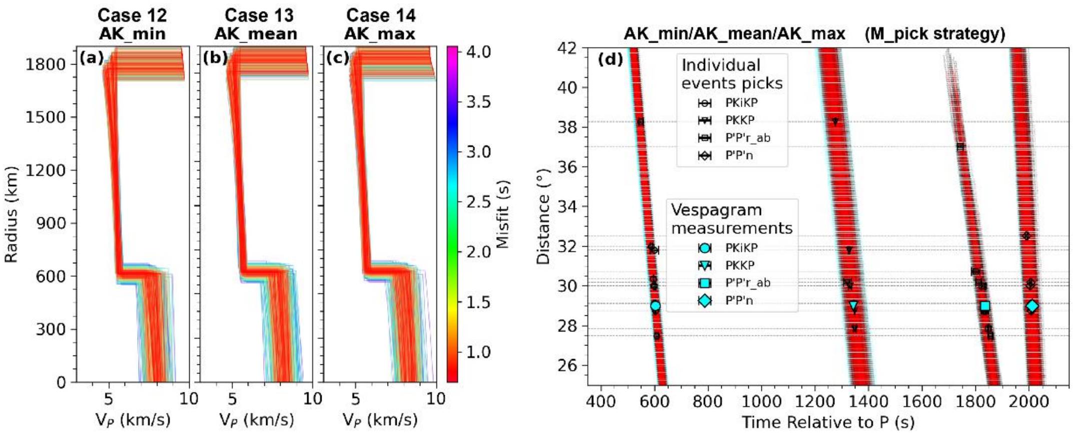

Fig. SS54. P velocity profiles of the Martian core based on the inverted parameters for three mantle models from the AK_subset models. (a) AK_min, (b) AK_mean, and (c) AK_max model. In (d), cyan, red, and black lines denote the predictions from the inverted core models based on the AK_min, AK_mean, and AK_max models, respectively.

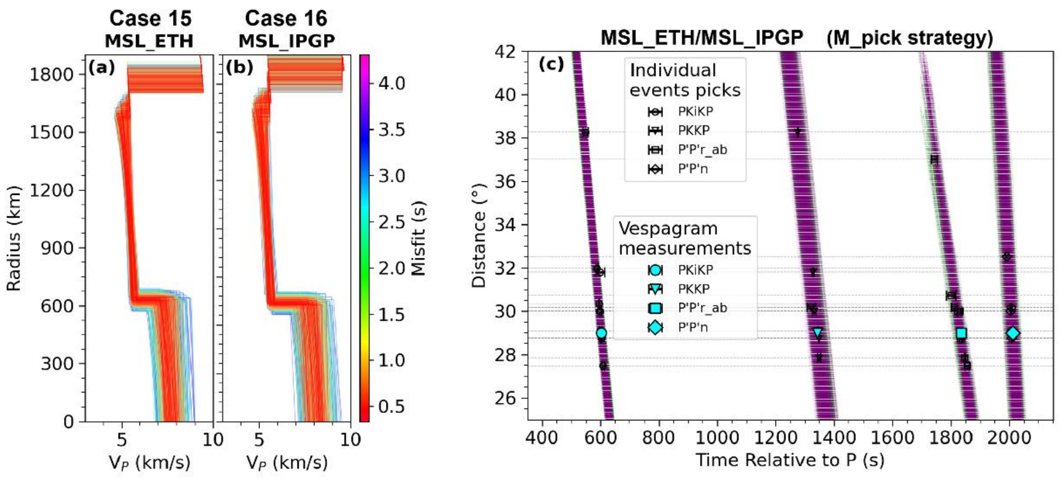

Fig. SS55. P velocity profiles of the Martian core based on the inverted parameters for two mantle models of (a) MSL_IPGP and (b) MSL_ETH. In (c), the green and purple lines denote predictions from the inverted core models based on MSL_IPGP and MSL_ETH models, respectively.
